# Supplementary figures and images for: A novel targeting domain directs essential components of the cytosolic iron–sulfur cluster assembly pathway to the mitochondrion of Toxoplasma parasites
Source: PLoS Biol. 2025 Nov 25;23(11):e3003520. doi: 10.1371/journal.pbio.3003520 (PMC12674569; doi:10.1371/journal.pbio.3003520)

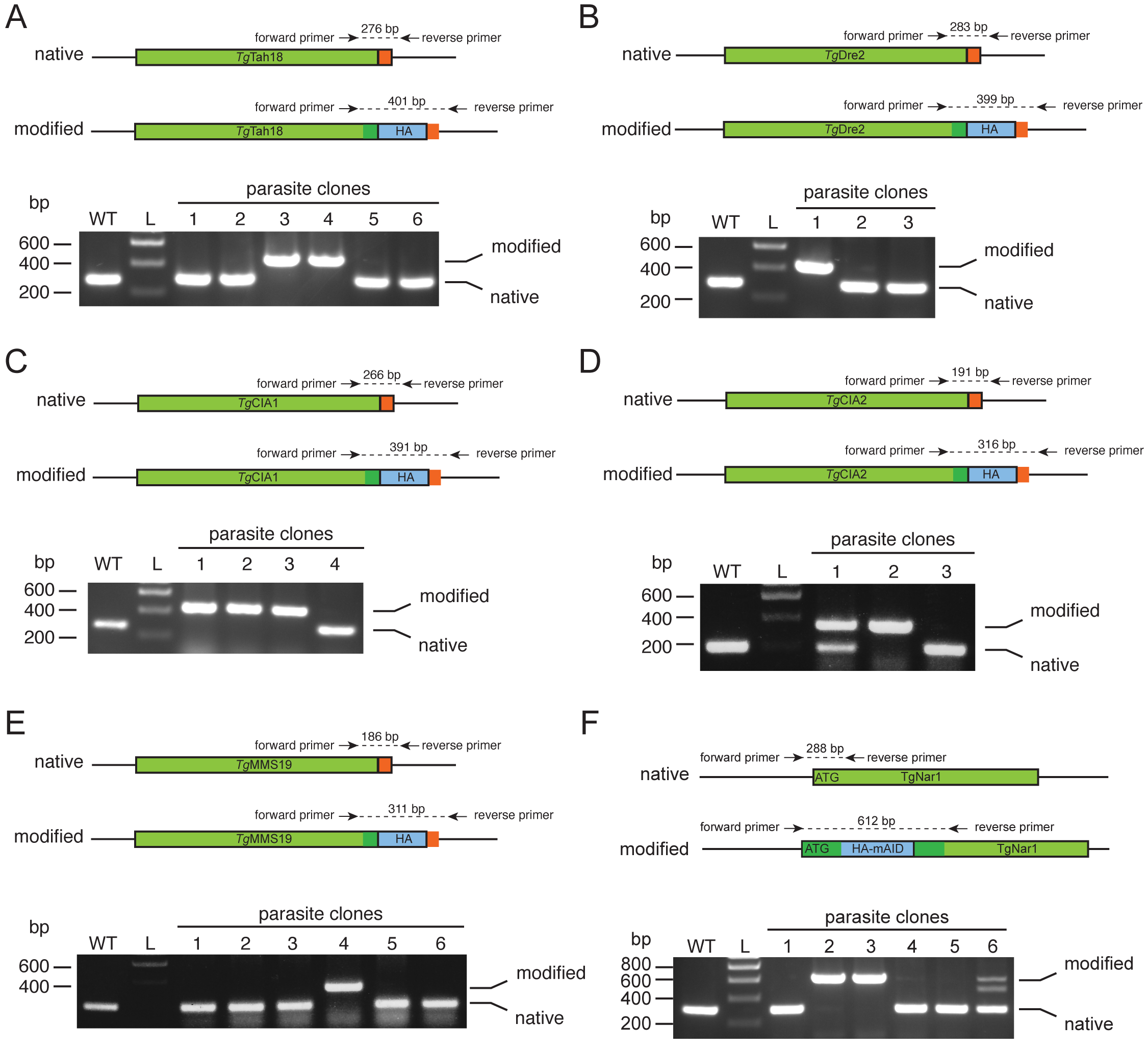

Supplement: S1 Fig — (A–E) 3× hemagglutinin epitope tags (HA) were integrated into the 3′ regions of the open reading frames of the genes encoding (A) TgTah18, (B) TgDre2, (C) TgCIA1, (D) TgCIA2, or (E) TgMMS19 in ATc-regulatable rTgNBP35-cMyc parasites. (F) A 3× hemagglutinin-mini-auxin inducible degron (HA-mAID) epitope tag was integrated into the 5′ region of the TgNar1 open reading frame in RH∆ku80/Tir1-FLAG/tdTomato parasites, generating the rHA-mAID-TgNar1 parasite line. A schematic depicting the target locus before and after modification, the approximate position of the forward and reverse primers used in the PCR analysis, and the expected sizes of the PCR products in the native and modified genomic loci, are shown at the top of each panel. The PCR screens testing for genetic modifications are shown at the bottom of each panel. PCRs were performed using forward and reverse primers specific to the target site of each gene, and using genomic DNA extracted from clonal parasite lines. Genomic DNA from a wild type (WT) parasite line was used as a control for the expected size of the native locus in each screen. (TIF) [file pbio.3003520.s001.tif]

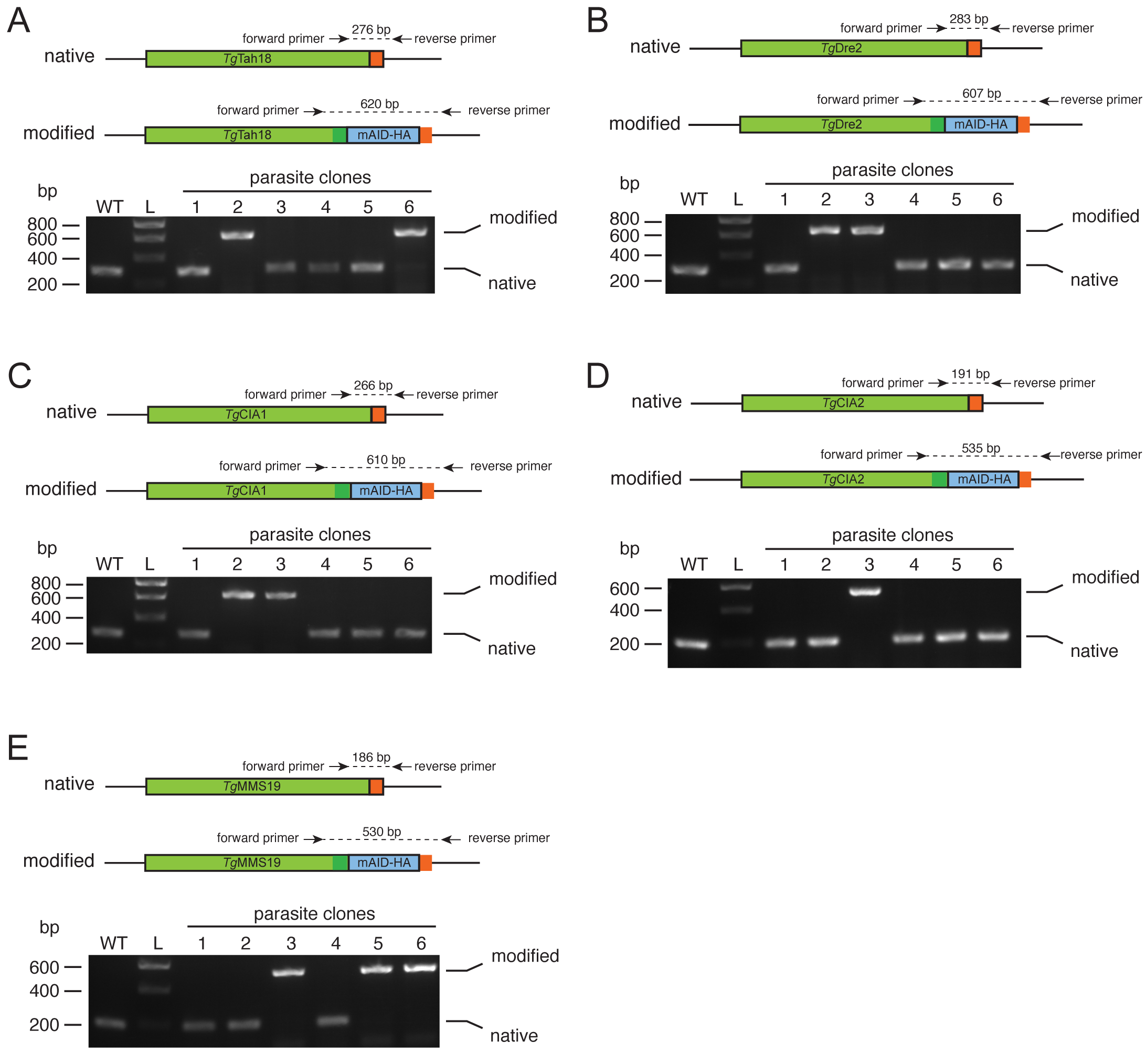

Supplement: S2 Fig — (A–E) Mini-auxin inducible degron-3× hemagglutinin (mAID-HA) epitope tags were integrated into the 3′ regions of open reading frames of the genes encoding (A) TgTah18, (B) TgDre2, (C) TgCIA1, (D) TgCIA2, or (E) TgMMS19 in RH∆ku80/Tir1-FLAG/tdTomato parasites generating the rTgTah18-mAID-HA, rTgDre2-mAID-HA, rTgCIA1-mAID-HA, rTgCIA2-mAID-HA, and rTgMMS19-mAID-HA parasite lines. A schematic depicting the target locus before and after modification, the approximate position of the forward and reverse primers used in the PCR analysis, and the expected sizes of the PCR products in the native and modified genomic loci, are shown at the top of each panel. The PCR screens testing for genetic modifications are shown at the bottom of each panel. PCRs were performed using forward and reverse primers specific to the target site of each gene, and using genomic DNA extracted from clonal parasite lines. Genomic DNA from a WT parasite line was used as a control for the expected size of the native locus in each screen. (TIF) [file pbio.3003520.s002.tif]

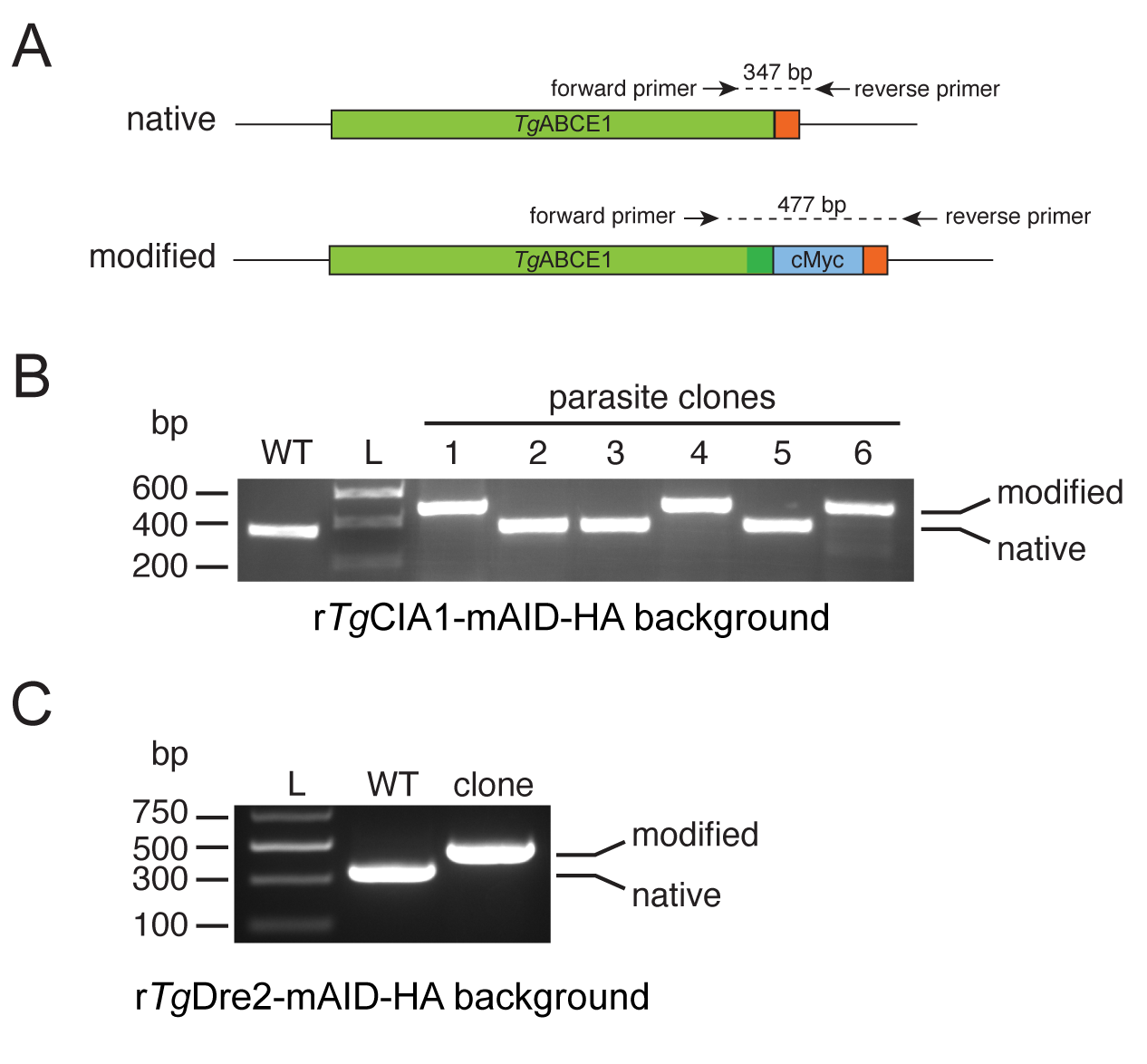

Supplement: S3 Fig — (A) A schematic depicting the TgABCE1 genomic locus before and after introduction of a 3× cMyc epitope tag into the 3′ region of the open reading frame of the gene, the approximate position of the forward and reverse primers used in the PCR analyses, and the expected sizes of the PCR products in the native and modified TgABCE1 loci. (B, C) PCR analyses using the forward and reverse primers and template genomic DNA extracted from (B) rTgCIA1-mAID-HA/TgABCE1-cMyc parasite clones and (C) a rTgDre2-mAID-HA/TgABCE1-cMyc parasite clone. Genomic DNA from a WT parasite line was used as a control for the expected size of the native locus. (TIF) [file pbio.3003520.s003.tif]

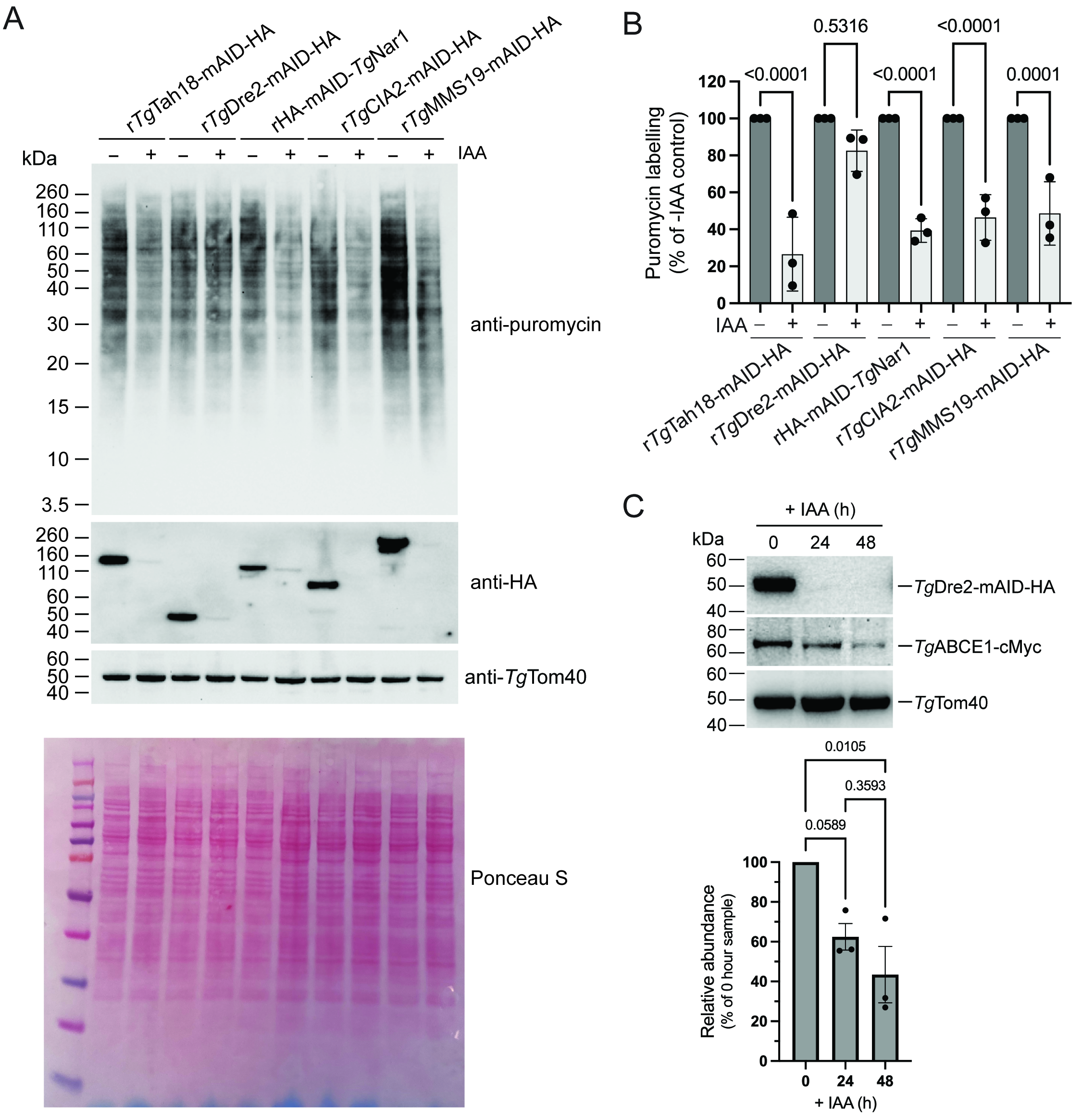

Supplement: S4 Fig — (A) Western blots measuring the incorporation of puromycin into proteins from rTgTah18-mAID-HA, rTgDre2-mAID-HA, rHA-mAID-TgNar1, rTgCIA2-mAID-HA, and rTgMMS19-mAID-HA parasites cultured in the absence or presence of IAA for 24 h, probed with anti-puromycin, anti-HA or anti-TgTom40 antibodies. The membrane was also stained following transfer with the protein-binding dye Ponceau S. (B) Relative abundance of puromycin incorporation into each parasite line was determined as a percentage of the -IAA control, with abundances normalized using the TgTom40 loading control. Data points represent the mean ± SD of three independent experiments. Data were analyzed using a one-way ANOVA followed by Tukey’s multiple comparisons test with relevant p values shown. (C) Western blots of proteins extracted from rTgDre2-mAID-HA/TgABCE1-cMyc parasites cultured for 0, 24, or 48 h in IAA and separated by SDS-PAGE. Samples were probed with anti-HA, anti-cMyc, and anti-TgTom40 antibodies. The relative abundance of the TgABCE1-cMyc protein in the western blot was determined as a percentage of the 0 h control, with abundances normalized using the TgTom40 loading control. Data points represent the mean ± SD of three independent experiments. Data were analyzed using a one-way ANOVA followed by Tukey’s multiple comparisons test, with p values shown. The numerical data underlying this Figure can be found in S1 Data. (TIF) [file pbio.3003520.s004.tif]

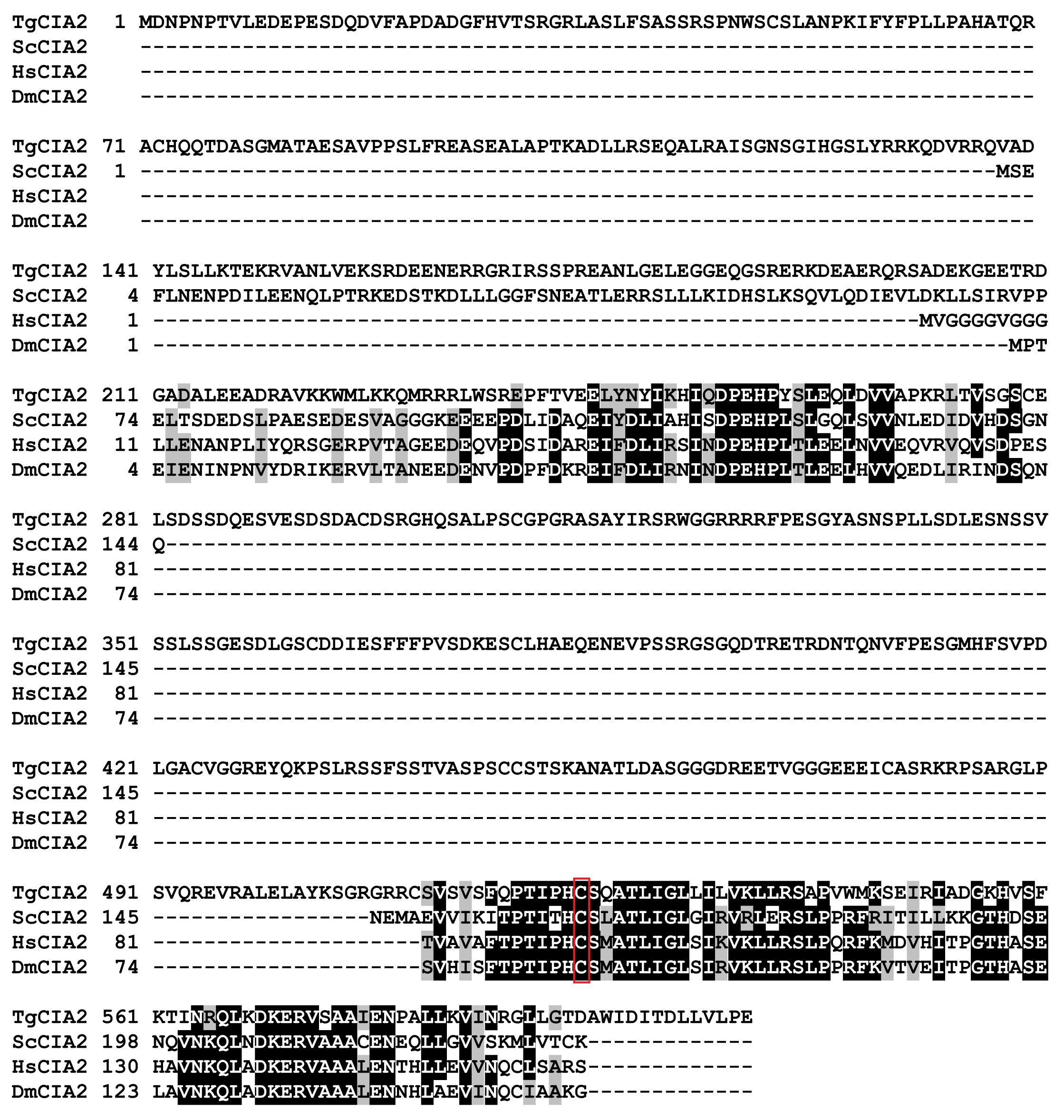

Supplement: S5 Fig — A multiple sequence alignment of the TgCIA2 protein with homologs from the yeast Saccharomyces cerevisiae (ScCIA2; UniProt accession number P38829), Homo sapiens (HsCIA2; UniProt Q9Y3D0), and the fruit fly Drosophila melanogaster (DmCIA2; UniProt Q9VTC4). The reactive cysteine residue of CIA2 that is proposed to function in FeS cluster binding is highlighted by a red box. (TIF) [file pbio.3003520.s005.tif]

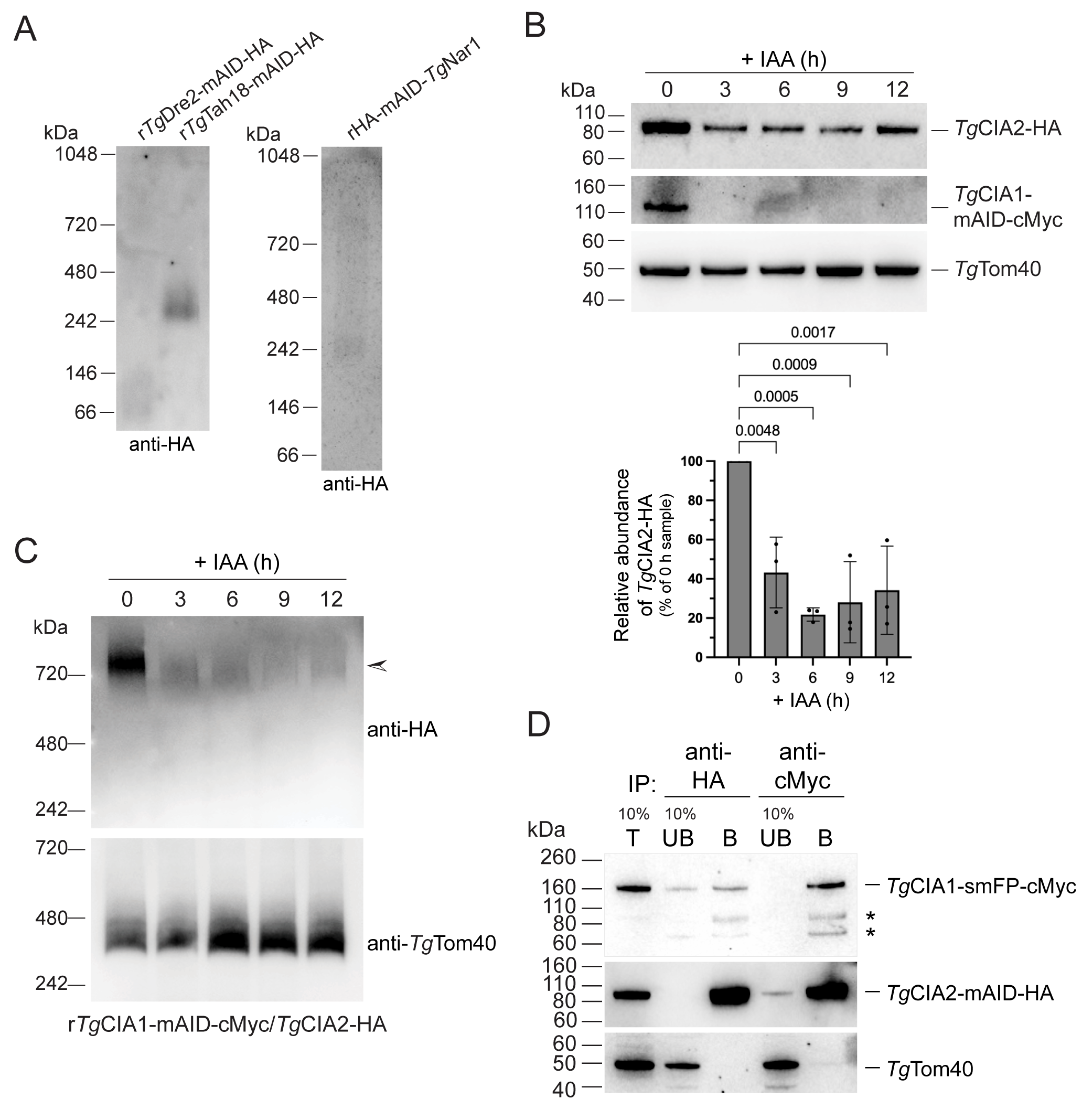

Supplement: S6 Fig — (A) Western blot of proteins extracted from rTgDre2-mAID-HA, rTgTah18-mAID-HA, and rHA-mAID-TgNar1/TgCIA1-smFP-cMyc parasites, separated by BN-PAGE and probed with anti-HA antibodies to detect the TgDre2-mAID-HA, TgTah18-mAID-HA, and HA-mAID-TgNar1 proteins. (B, C) Western blots of proteins extracted from rTgCIA1-mAID-cMyc/TgCIA2-HA parasites cultured for 0–12 h in IAA, separated by (B) SDS-PAGE or (C) BN-PAGE and probed with anti-HA antibodies to detect the TgCIA2-HA protein, anti-cMyc antibodies to detect the TgCIA1-mAID-cMyc protein, and anti-TgTom40 antibodies as a loading control. For the SDS-PAGE western blots (B), the relative abundance of the TgCIA2-HA protein was determined as a percentage of the 0 h IAA control, with abundances normalized using the TgTom40 loading control. Data points represent the mean ± SD of three independent experiments. Data were analyzed using a one-way ANOVA followed by Tukey’s multiple comparisons test with relevant p values shown. The numerical data underlying this Figure can be found in S1 Data. In the BN-PAGE western blot (C), the black arrowhead indicates the >720 kDa candidate CIA Targeting Complex. (D) Western blot of proteins extracted from rTgCIA2-mAID-HA/TgCIA1-smFP-cMyc parasites and immunoprecipitated using anti-HA- or anti-cMyc-conjugated agarose beads. Extracted fractions include total protein prior to immunoprecipitation (T), unbound proteins (U), and antibody-bound proteins (B) in the indicated proportions. Protein fractions were separated by SDS-PAGE and probed with anti-cMyc, anti-HA, or anti-TgTom40 antibodies. Data are representative of three independent experiments for the anti-HA immunoprecipitation and two independent experiments for the anti-cMyc immunoprecipitation. Asterisks depict likely degradation products of the TgCIA1-smFP-cMyc protein. (TIF) [file pbio.3003520.s006.tif]

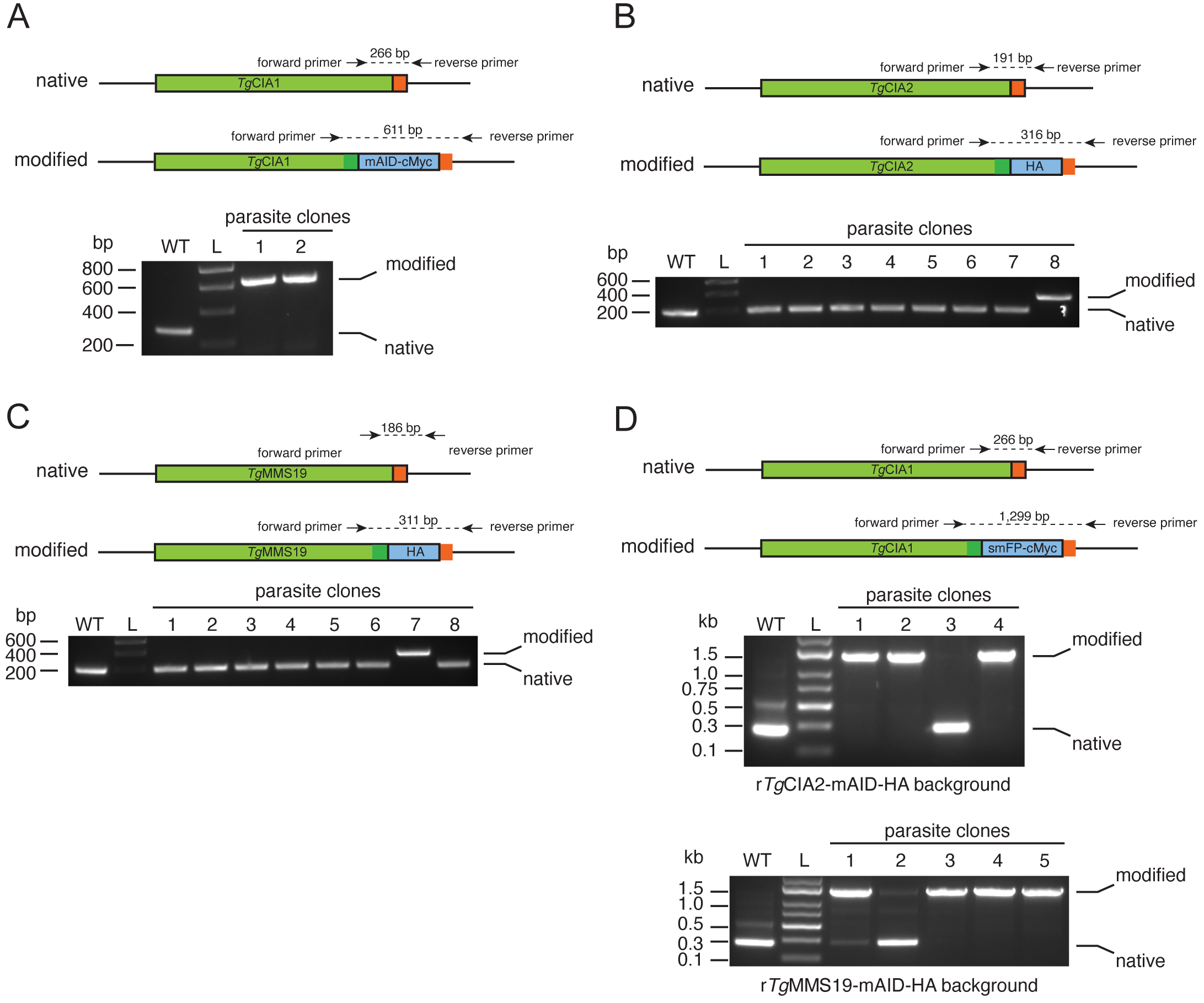

Supplement: S7 Fig — (A) A mAID-cMyc epitope tag was integrated into the 3′ region of the open reading frames of TgCIA1 in RH∆ku80/Tir1-FLAG/tdTomato parasites, generating the rTgCIA1-mAID-cMyc parasite line. (B, C) HA epitope tags were integrated into the (B) TgCIA2 or (C) TgMMS19 loci of the rTgCIA1-mAID-cMyc parasite line, generating the rTgCIA1-mAID-cMyc/TgCIA2-HA and rTgCIA1-mAID-cMyc/TgMMS19-HA parasite lines. (D) A spaghetti monster fluorescent protein-cMyc (smFP-cMyc) epitope tag was integrated into the 3′ region of the open reading frame of TgCIA1 in IAA-regulatable rTgCIA2-mAID-HA (top) or rTgMMS19-mAID-HA (bottom) parasites, generating the rTgCIA2-mAID-HA/TgCIA1-smFP-cMyc and rTgMMS19-mAID-HA/TgCIA1-smFP-cMyc parasite lines. A schematic depicting the target locus before and after modification, the approximate position of the forward and reverse primers used in the PCR analysis, and the expected sizes of the PCR products in the native and modified genomic loci, are shown at the top of each panel. The PCR analyses testing for genomic modifications are shown at the bottom of each panel. PCRs were performed using forward and reverse primers specific to the target site of each gene, and using genomic DNA extracted from clonal parasite lines. Genomic DNA from a WT parasite line was used as a control for the expected size of the native locus in each screen. (TIF) [file pbio.3003520.s007.tif]

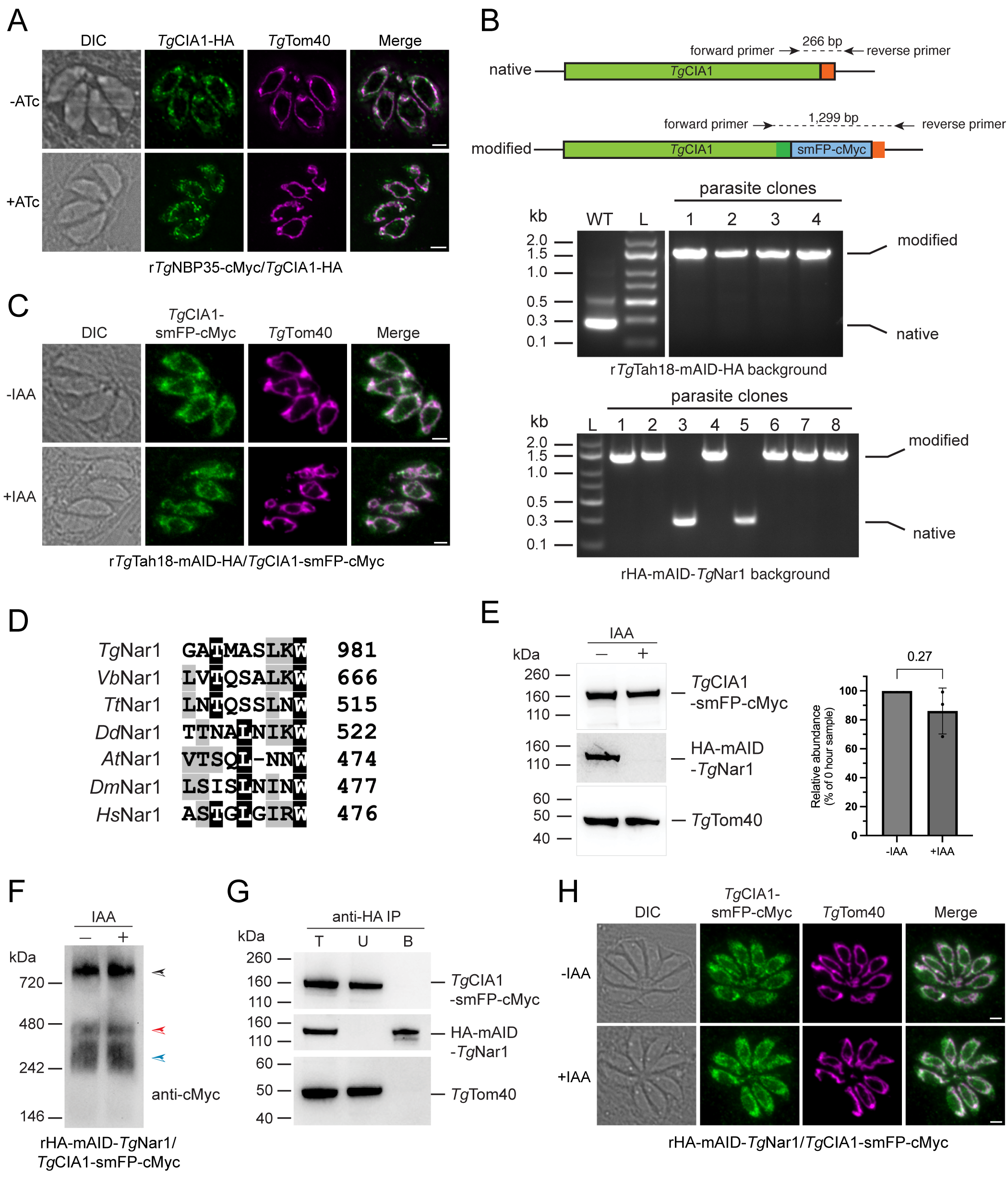

Supplement: S8 Fig — (A) Immunofluorescence assays of rTgNBP35-cMyc/TgCIA1-HA parasites, cultured in the absence (top) or presence (bottom) of ATc for two days. Samples were probed with anti-HA to detect TgCIA1-HA (green) and anti-TgTom40 antibodies to detect the mitochondrion (magenta). (B) A spaghetti monster fluorescent protein-cMyc (smFP-cMyc) epitope tag was integrated into the 3′ region of the open reading frame of TgCIA1 in IAA-regulatable rTgTah18-mAID-HA (top) or rHA-mAID-TgNar1 (bottom) parasites, generating the rTgTah18-mAID-HA/TgCIA1-smFP-cMyc and rHA-mAID-TgNar1/TgCIA1-smFP-cMyc parasite lines. A schematic depicting the target locus before and after modification, the approximate position of the forward and reverse primers used in the PCR analysis, and the expected sizes of the PCR products in the native and modified genomic loci, are shown at the top of the panel. The PCR analyses testing for genomic modifications are shown at the bottom of the panel. Note that the PCR screens for the candidate rTgTah18-mAID-HA/TgCIA1-smFP-cMyc clones was performed on the same gel as the rTgCIA2-mAID-HA/TgCIA1-smFP-cMyc clone (S7D Fig) and therefore has the same ladder and WT control. (C) Immunofluorescence assays of rTgTah18-mAID-HA/TgCIA1-smFP-cMyc parasites cultured in the absence (top) or presence (bottom) of IAA for 24 h. Samples were probed with anti-cMyc to detect TgCIA1-smFP-cMyc (green) and anti-TgTom40 antibodies to detect the mitochondrion (magenta). (D) Multiple sequence alignment of the C-terminal region of the Toxoplasma gondii Nar1 protein (TgNar1) with Nar1 homologs from the chrompodellid Vitrella brassicaformis (VbNar1; www.veupathdb.org accession number Vbra_21454; [60]), the ciliate Tetrahymena thermophila (TtNar1; UniProt accession number Q22NP0), the amoebozoan Dictyostelium discoideum (DdNar1; UniProt Q54F30), the plant Arabidopsis thaliana (AtNar1; UniProt Q94CL6), and the animals D. melanogaster (DmNar1; UniProt Q8SYS7) and H. sapiens (HsNar1; NCBI accession number N [file pbio.3003520.s008.tif]

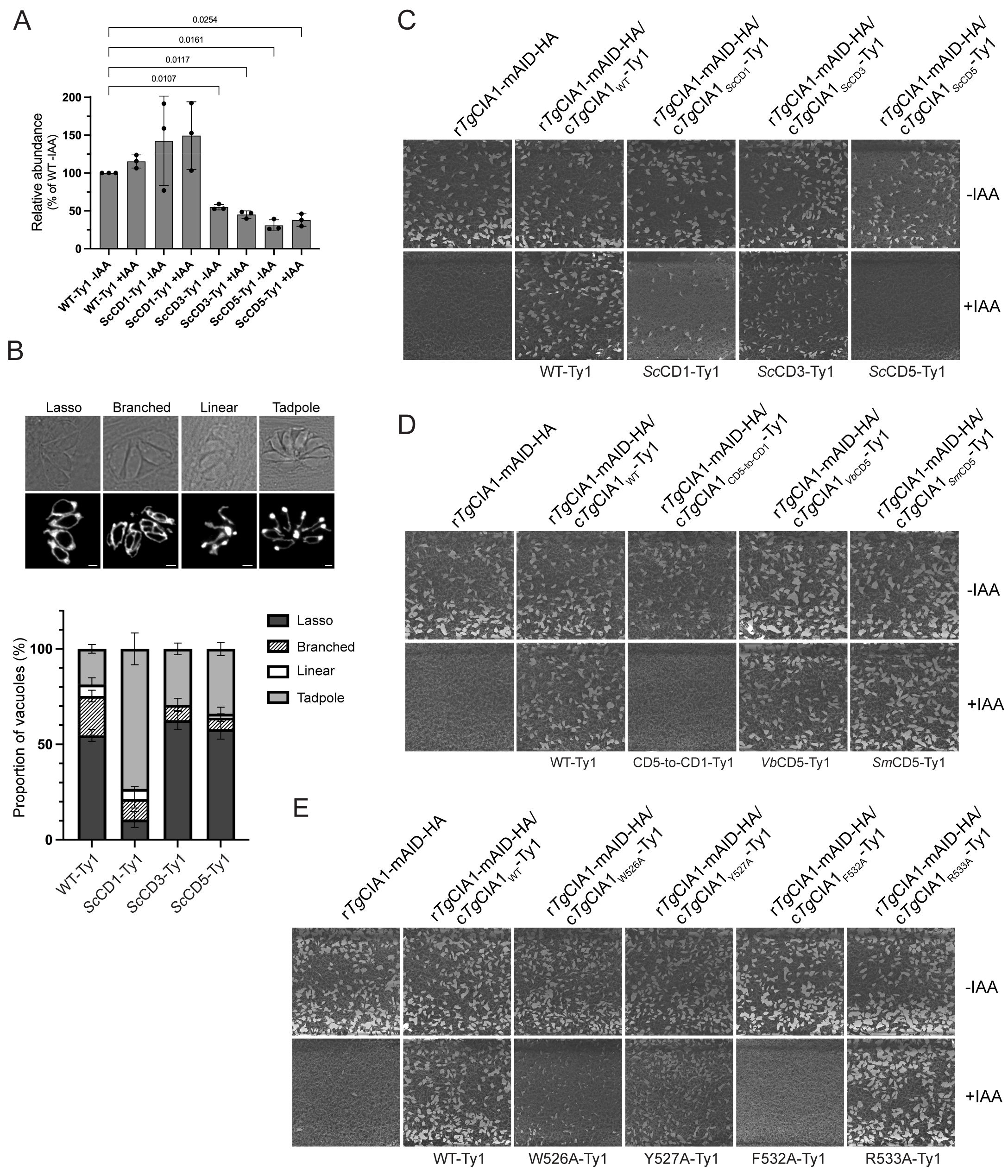

Supplement: S10 Fig — (A) Relative abundances of proteins depicted in the Fig 6B western blots were determined as a percentage of the -IAA condition for the cTgCIA1WT-Ty1 protein and normalized using the TgTom40 loading control. Data points represent the mean ± SD of three independent experiments. Data were analyzed using a one-way ANOVA followed by Tukey’s multiple comparisons test, with significant p values (<0.05) with respect to the -IAA condition of the cTgCIA1WT-Ty1-IAA protein shown. (B) Quantification of mitochondrial morphology in rTgCIA1-mAID-HA/cTgCIA1WT-Ty1 (WT-Ty1), rTgCIA1-mAID-HA/cTgCIA1ScCD1-Ty1 (ScCD1-Ty1), rTgCIA1-mAID-HA/cTgCIA1ScCD3-Ty1 (ScCD3-Ty1), and rTgCIA1-mAID-HA/cTgCIA1ScCD5-Ty1 (ScCD5-Ty1) parasites. Mitochondria were observed by immunofluorescence assays using anti-TgTom40 antibodies, and were classified as lasso, branched, linear, or tadpole shaped, with representative images of each category shown above. The morphologies of mitochondria in 150 vacuoles containing 4–16 intracellular parasites were determined in each parasite line across three independent experiments, with the observer blinded to the identities of the samples being examined. (C–E) Plaque assays of rTgCIA1-mAID-HA parasites and rTgCIA1-mAID-HA parasites constitutively expressing TgCIA1 variants, including (C) cTgCIA1WT-Ty1 (WT-Ty1), cTgCIA1ScCD1-Ty1 (ScCD1-Ty1), cTgCIA1ScCD3-Ty1 (ScCD3-Ty1) and cTgCIA1ScCD5-Ty1 (ScCD5-Ty1), (D) cTgCIA1WT-Ty1 (WT-Ty1), cTgCIA1CD5-to-CD1-Ty1 (CD5-to-CD1-Ty1), cTgCIA1VbCD5-Ty1 (VbCD5-Ty1), and cTgCIA1SmCD5-Ty1 (SmCD5-Ty1), and (E) cTgCIA1WT-Ty1 (WT-Ty1), cTgCIA1W526A-Ty1 (W526A-Ty1) cTgCIA1Y527A-Ty1 (Y527A-Ty1), cTgCIA1F532A-Ty1 (F532A-Ty1), and cTgCIA1R533A-Ty1 (R533A). Parasites were cultured in the absence (top) or presence (bottom) of IAA for six days and are representative of three independent experiments. Each plaque assay was set up simultaneously with the fluorescence proliferation assays depicted in Figs 6F, 7C, 8I, 9D, and S12G. The numerical data under [file pbio.3003520.s010.tif]

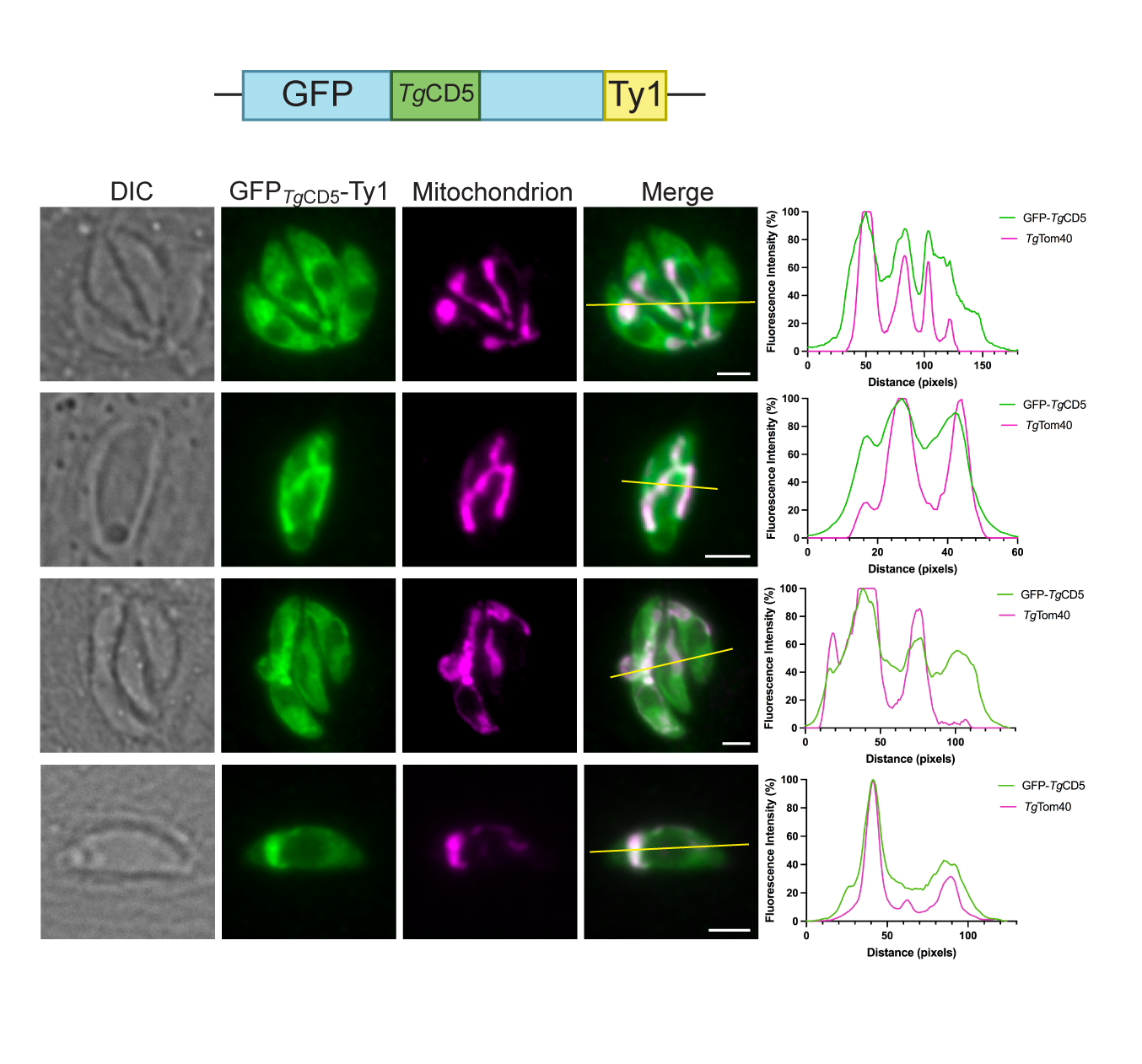

Supplement: S11 Fig — Immunofluorescence assay of parasites constitutively expressing a GFP-Ty1 variant containing the CD5 loop of TgCIA1 between the eighth and ninth β-strands of GFP (GFPTgCD5-Ty1), probed with anti-Ty1 antibodies to detect the GFPTgCD5-Ty1 protein (green) and anti-TgTom40 antibodies to detect the mitochondrion (magenta). Scale bars are 2 µm. DIC, differential interference contrast. Right, corresponding fluorescence plots depicting the intensity of anti-Ty1 (green) and anti-TgTom40 (magenta) labeling along the yellow line in merged images. The numerical data underlying this Figure can be found in S1 Data. (TIF) [file pbio.3003520.s011.tif]

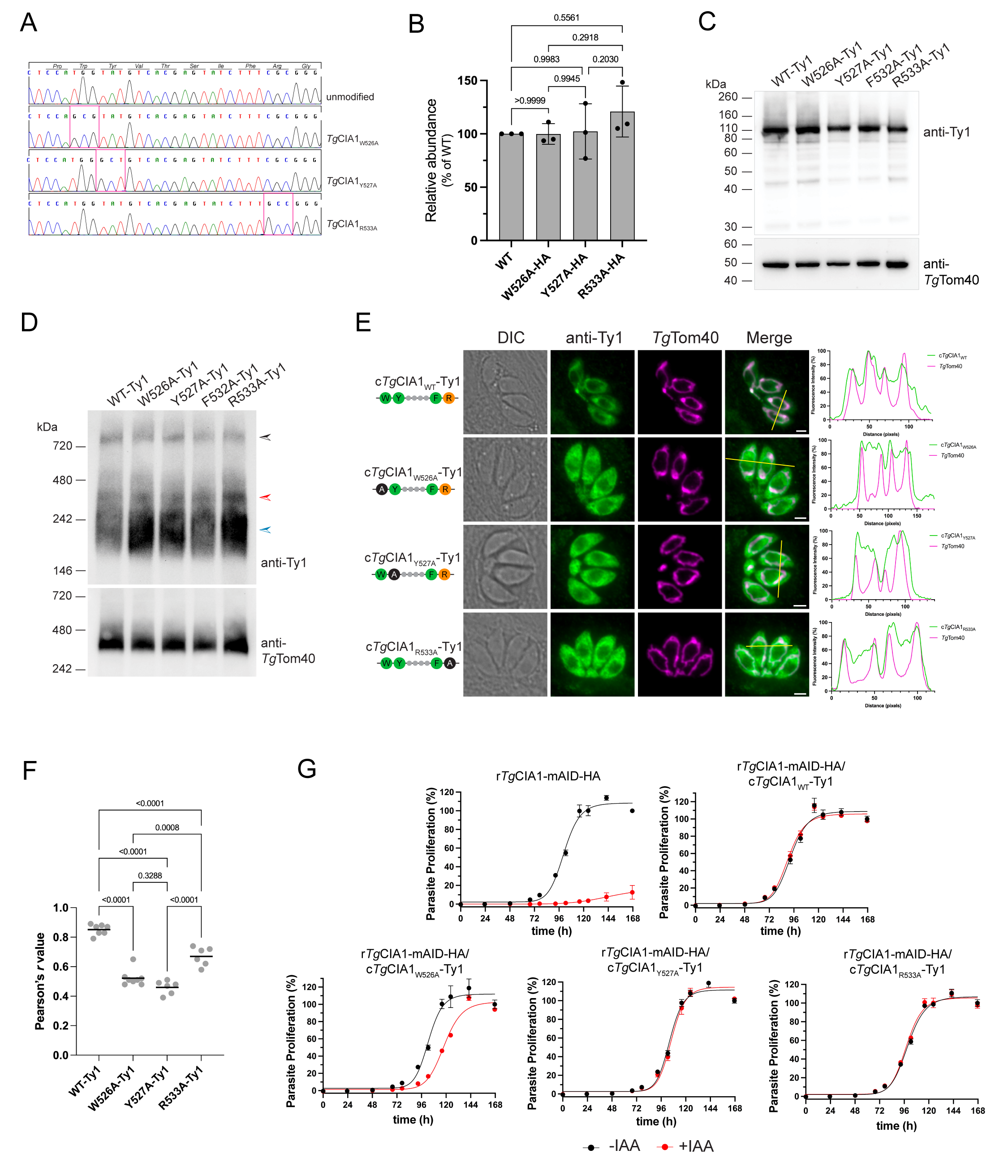

Supplement: S12 Fig — (A) Sanger DNA sequencing chromatograms depicting the nucleotides modified in the TgCIA1 gene to generate substitutions of the W526, Y527, and R533 residues of the protein to alanine. Mutated codons for each line are highlighted with a magenta box. (B) The relative abundance of proteins depicted in Fig 8B was determined as a percentage of the WT-HA protein and normalized using the TgTom40 loading control. Data points represent the mean ± SD of three independent experiments. Data were analyzed using a one-way ANOVA followed by Tukey’s multiple comparisons test with p values shown. (C, D) Western blots of proteins extracted from rTgCIA1-mAID-HA/cTgCIA1WT-Ty1 (WT-Ty1), rTgCIA1-mAID-HA/cTgCIA1W526A-Ty1 (W526A-Ty1), rTgCIA1-mAID-HA/cTgCIA1Y527A-Ty1 (Y527A-Ty1), rTgCIA1-mAID-HA/cTgCIA1F532A-Ty1 (F532A-Ty1), and rTgCIA1-mAID-HA/cTgCIA1R533A-Ty1 (R533A-Ty1) parasites, separated by (C) SDS-PAGE or (D) BN-PAGE, and probed with anti-Ty1 or anti-TgTom40 antibodies. The black arrowhead indicates the >720 kDa CIA Targeting Complex; red and blue arrowheads indicate the lower mass complexes containing the TgCIA1 protein. (E) Immunofluorescence assays of rTgCIA1-mAID-HA/cTgCIA1WT-Ty1 (WT-Ty1; also shown in Fig 8G), rTgCIA1-mAID-HA/cTgCIA1W526A-Ty1 (W526A-Ty1), rTgCIA1-mAID-HA/cTgCIA1Y527A-Ty1 (Y527A-Ty1), and rTgCIA1-mAID-HA/cTgCIA1R533A-Ty1 (R533A-Ty1) parasites. The complemented proteins of interest (green) and the mitochondrion (magenta) were labeled with anti-Ty1 and anti-TgTom40 antibodies, respectively. Schematics depicting the modified amino acid sequence in the CD5 motif of the proteins from each panel are included next to images (left). Scale bars are 2 µm. DIC, differential interference contrast. Right, corresponding fluorescence profile depicting intensity of anti-Ty1 (green) and anti-TgTom40 (magenta) labeling along the yellow lines of the merged images. (F) The correlation between Ty1-tagged proteins and TgTom40 was quantified using the Pearson correlation coefficient ( [file pbio.3003520.s012.tif]

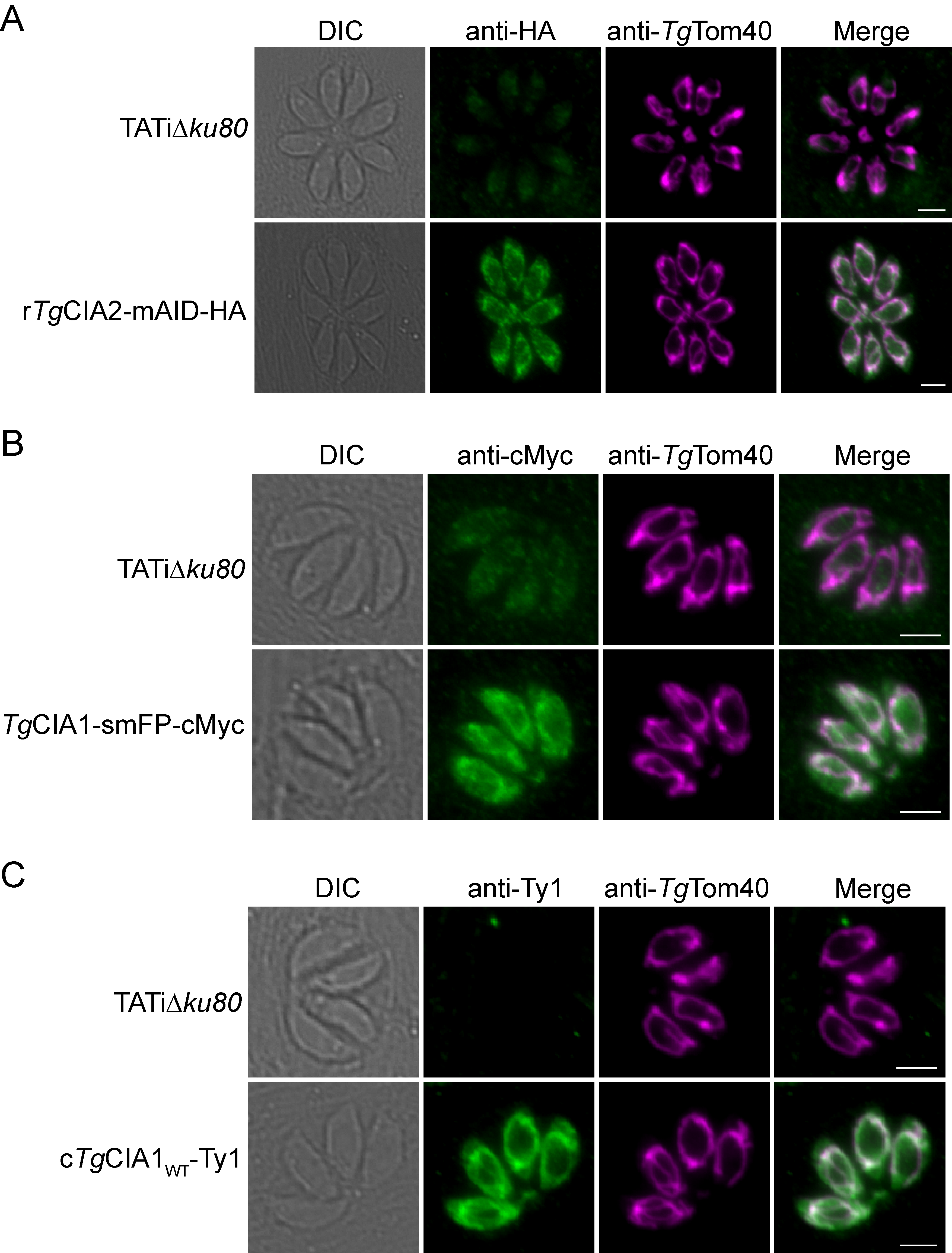

Supplement: S13 Fig — (A–C) Immunofluorescence assays of TATi∆ku80 parasites lacking epitope tags (A–C, top) and epitope-tagged positive control parasites (A, B bottom, TgCIA2-mAID-HA/TgCIA1-smFP-cMyc parasites; C bottom, TgCIA1-mAID-HA/cTgCIA1WT-Ty1 parasites) were performed to test for non-specific antibody labeling in untagged parasites. Parasite samples were probed on the same day with (A) anti-HA (green) and anti-TgTom40 (magenta) antibodies, (B) anti-cMyc (green), and anti-TgTom40 (magenta) antibodies, and (C) anti-Ty1 (green) and anti-TgTom40 (magenta) antibodies using the same antibody dilutions for the negative and positive control samples. For image processing, the contrast and brightness of a positive control image of each sample was adjusted linearly, and the same adjustments (i.e., the same minimum and maximum pixel intensities) were applied to TATi∆ku80 parasites. Scale bars are 2 µm. DIC, differential interference contrast transmission image. (TIF) [file pbio.3003520.s013.tif]
