# Supplementary material for: A novel targeting domain directs essential components of the cytosolic iron–sulfur cluster assembly pathway to the mitochondrion of Toxoplasma parasites
Source: PLoS Biol. 2025 Nov 25;23(11):e3003520. doi: 10.1371/journal.pbio.3003520 (PMC12674569; doi:10.1371/journal.pbio.3003520)
Supplement: S9 Fig — A multiple sequence alignment of the TgCIA1 protein with homologs from the dinozoans Perkinsus marinus (NCBI accession number: XP_002785992.1) and Symbiodinium microadriaticum (NCBI CAE7872903.1), the apicomplexans Plasmodium falciparum (www.veupathdb.org accession number PF3D7_1209400; [60]) and Cryptosporidium parvum (VEuPathDB cgd1_2230), the chrompodellids Vitrella brassicaformis (VEuPathDB Vbra_18342), and Chromera velia (VEuPathDB Cvel_5245), the yeast Saccharomyces cerevisiae (UniProt accession number Q05583), the ciliate Paramecium tetraurelia (UniProt A0BLF7), the plant Arabidopsis thaliana (UniProt F4JVW1), the amoebozoan Dictyostelium discoideum (UniProt Q55DA2), the animal Drosophila melanogaster (UniProt Q7K1Y4) and the oomycete Phytophthora infestans (UniProt D0NMX9). The positions of each of the seven blades of the β-propellor structure of CIA1 are indicated. Sequences corresponding to the CD loops of interest are highlighted in pink (CD1 loop), yellow (CD3 loop), and green (CD5 loop), with the remaining CD loops and the AB loop of blade 7 (which is slightly extended in TgCIA1) highlighted in blue. The alignment was generated in Clustal Omega and plotted using pyBoxshade. (PDF) [file pbio.3003520.s009.pdf]

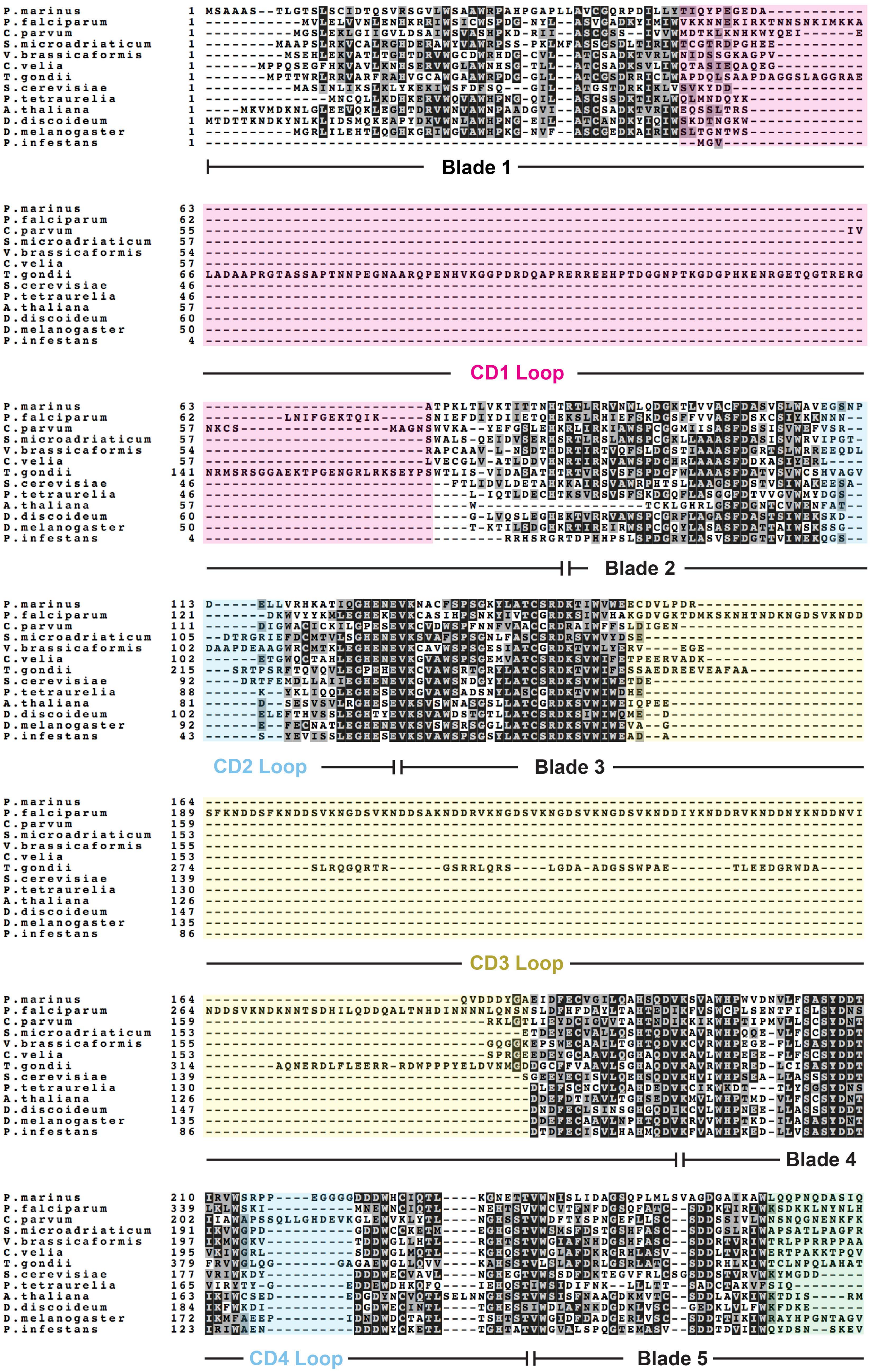
P.marinus
P.falciparum
C.parvum
S.microadriaticum
V.brassicaformis
C.velia
T.gondii
S.cerevisiae
P.tetraurelia
A.thaliana
D.discoideum
D.melanogaster
P.infestans
Blade 1
CD1 Loop
Blade 2
CD2 Loop
Blade 3
CD3 Loop
Blade 4
CD4 Loop
Blade 5

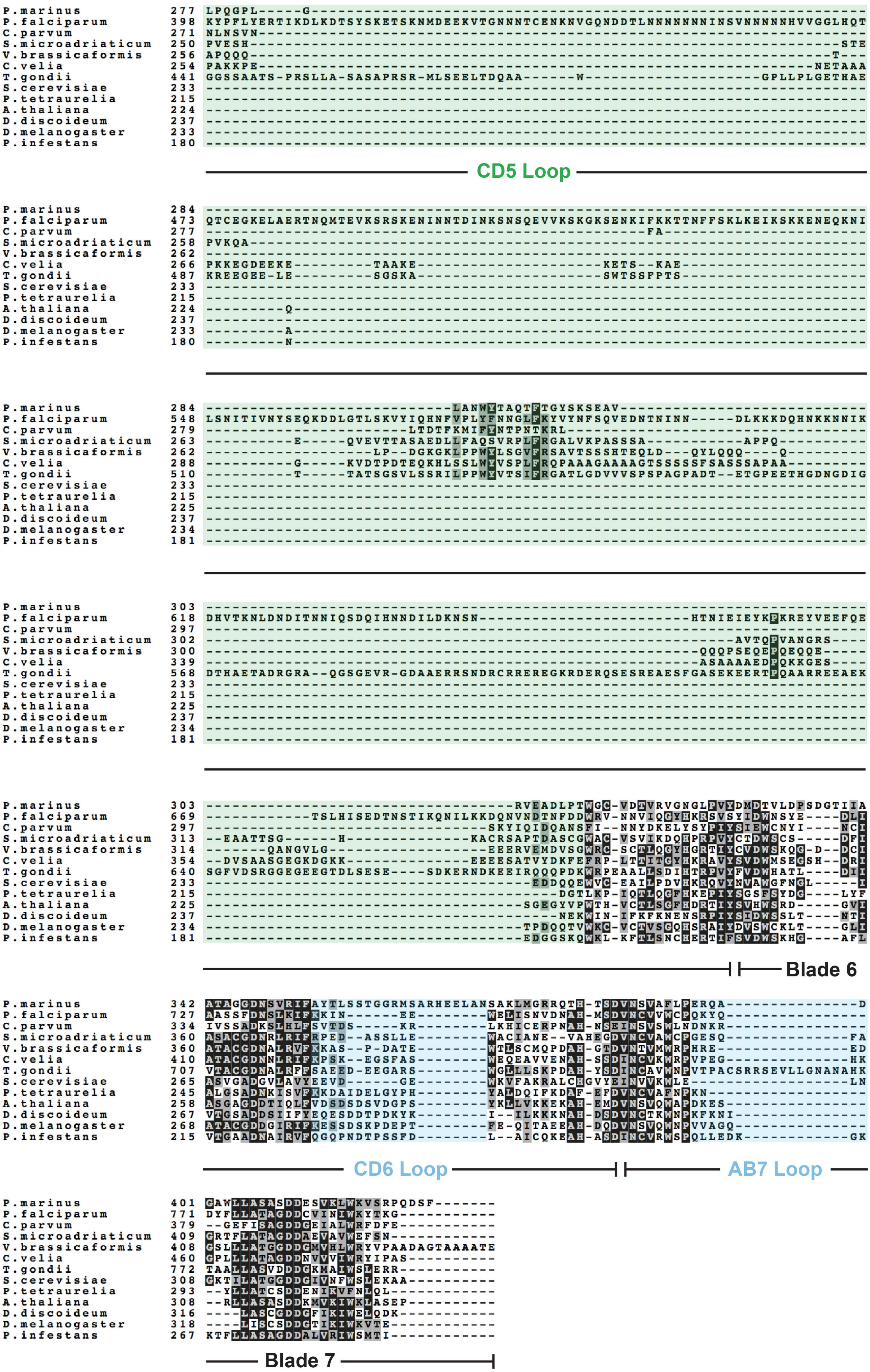
P.marinus
P.falciparum
C.parvum
S.microadriaticum
V.brassicaformis
C.velia
T.gondii
S.cerevisiae
P.tetraurelia
A.thaliana
D.discoideum
D.melanogaster
P.infestans
CD5 Loop
Blade 6
CD6 Loop
AB7 Loop
Blade 7
