# Supplementary material for: A novel targeting domain directs essential components of the cytosolic iron–sulfur cluster assembly pathway to the mitochondrion of Toxoplasma parasites
Source: PLoS Biol. 2025 Nov 25;23(11):e3003520. doi: 10.1371/journal.pbio.3003520 (PMC12674569; doi:10.1371/journal.pbio.3003520)
Supplement: S1 Table — (PDF) [file pbio.3003520.s014.pdf]

S1 Table. Oligonucleotides and gBlocks used in this study (all oligonucleotides are listed in a 5' to 3' orientation; sgRNA-coding sequences are underlined).

Tah18 3' CRISPR fwd	AGATACGTCTGTGATACTTGGTTTTAGAGCTAGAAATAGCAAG
Dre2 3' CRISPR fwd	GGTGCAGATTACAGATCTCGGTTTTAGAGCTAGAAATAGCAAG
CIA1 3' CRISPR fwd	GCGTCTCTCTAGACTCCATAGTTTTAGAGCTAGAAATAGCAAG
CIA2 3' CRISPR fwd	GTTCTCTCTTTTACTCAGGGGTTTTAGAGCTAGAAATAGCAAG
MMS19 3' CRISPR fwd	CAGCCGCAGTGTGCAAGAATGTTTTAGAGCTAGAAATAGCAAG
Nar1 5' CRISPR fwd	CCATCGACATCAGCAACCACGTTTTAGAGCTAGAAATAGCAAG
ABCE1 3' CRISPR fwd	GCTCGACGACGCTTGAAGCGGTTTTAGAGCTAGAAATAGCAAG
CIA1-W526/Y527 CRISPR fwd	ATACTCGTGACATACCATGGGTTTTAGAGCTAGAAATAGCAAG
CIA1-F532/R533 CRISPR fwd	TGTCACGAGTATCTTTCGCGGTTTTAGAGCTAGAAATAGCAAG
universal CRISPR rvs	AACTTGACATCCCCATTTAC
Tah18 tag fwd	GTTTTCTTGCCGACAAAAAACGCAGAAAAAGATACGTCTGTGATACTTGG agcGGTGGAGGTAGCGGTGGTGGAAG
Tah18 tag rvs	AGTATACGTCTCTGACTGTTGTGGCGAATTGCGTGTCGAAGCAGGTTTGAG CTTCTGTGGGCGGTTATCAGG
Dre2 tag fwd	GTGGCAACTGTAATGAGCAACAAGGTGCAGATTACAGATCTCGGaGACGAT ATGGGTGGAGGTAGCGGTGGTGGAAG
Dre2 tag rvs	TGTGTAGAACGAAAACAAAGCTGCTCCCCGACGAGAAGCGGCGCCTCGTG GCTTCTGTGGGCGGTTATCAGG
Nar1 N-term tag fwd	ATACGGAGACATAGGCATCGCATTATTAGCGCCCAGTaCTGTGGTTGCTGAA AATGGCATACCCATACGACG
Nar1 N-term tag fwd	GCCAGCTTGACAGCTGCGCTGAAGGACGGGTTAGCGACGCTTTCTCGCGC ACTTCCACCACTGCTACCGCTAC
CIA1 tag fwd	CTGCTGGCGTCGGTCGATGACGACGGGAAGATGGCaATATGGAGTCTAGAG AGACGCGGTGGAGGTAGCGGTGGTGGAAG
CIA1 tag rvs	ACTCTCTCCTTCTCTATGGCTTTCTGTTTCTTGGAAAACAACAAAGCTCAGCT TCTGTGGGCGGTTATCAGG
CIA2 tag fwd	TAGGAACGGACGCGTGGATTGACATCACCGAGCTTCTCGTCCTCCCTGAGG GTGGAGGTAGCGGTGGTGGAAG
CIA2 tag rvs	ACTTGGATGATACATGAACTGAGGAGTAATGCGCTATGGCGTTCTCTCTTGC TTCTGTGGGCGGTTATCAGG
MMS19 tag fwd	TCATCAGGAGAGCAGCCGCAGTGTGCAAGAATCGaTGGTCCGTCTTCGAG GGTGGAGGTAGCGGTGGTGGAAG
MMS19 tag rvs	AGCCTCTCTCGGTTCCTTCACGTTCCTTTCTCTCTGTCCTCGTCGCTTCGGC TTCTGTGGGCGGTTATCAGG
ABCE1 tag fwd	AGGATAAAGAGCAGAAACTTATGGGCAACTATTTCATGCTCGACGACGCTG GTGGAGGTAGCGGTGGTGGAAG
ABCE1 tag rvs	CCCTGAGCGACCGGTCGGAAAAAAATTTGTCTTTCCCGGAAACAACCCTG GCTTCTGTGGGCGGTTATCAGG
Tah18 3' scrn fwd	TTCCTTTCCCGTGACTCTACTC
Tah18 3' scrn rvs	ATACGTCTCTGACTGTTGTGGC
Dre2 3' scrn fwd	GTCTGGAGTTTTGTGTGTGGAA
Dre2 3' scrn rvs	CATTTCCCATACTGACGAAACA

Nar1 5' scrn fwd	TAATTCCAAAGTTTTTGCCACC
Nar1 5' scrn rvs	AGTCCTGGTACTCCATTCCCTC
CIA1 3' scrn fwd	TTGGTTATTTTCCTTCTGTGGC
CIA1 3' scrn rvs	TTACTTCTTTCTGCTTCTCCCG
CIA2 3' scrn fwd	CTCTTTGATGTTGTGCTTTTGC
CIA2 3' scrn rvs	CGAGGTGAATATCAACTCTCCC
MMS19 3' scrn fwd	TCTGTGTTCAGGTCCTTGCAG
MMS19 3' scrn rvs	AGAATCCCTGTCCGACCCTT
ABCE1 3' scrn fwd	TGGAGTGCGTCGCTCGTT
ABCE1 3' scrn rvs	GGACACAGATACGCAGAGATACACAG
CIA2 comp fwd	gatcagatctaaaATGGACAATCCAAATCCTACGG
Ty1 rvs	CTAGCCCGGGGCTTCTGTGGGCGGTTATCAGG
CIA1 comp fwd	gatcagatctaaaATGCCTACCACTTGGCGTCTC
CIA1$_{ScCD3}$ fwd	atcgggaGGTTGCTTCTTCGTCGCT
CIA1$_{ScCD3}$ rvs	GATAAAACGGTCTGGATTTTCGAGacggacga
GFP fwd	GATCAGATCTAAAATGGCCGGAGTGTCGAA
GFP rvs	GATCCCTAGGACTTCCACCACCGCTACCTCCAC
*Dm*CIA1 fwd	gatcagatctaaaATGGGACGTTTAATTCTGGAACAC
CIA1$_{W526A}$ fwd	TTTGCCTCCTgcgTATGTCACGAG
CIA1$_{W526A}$ rvs	ATCCTTGAGGAGAGCACAC
CIA1$_{Y527A}$ fwd	GCCTCCTTGGgctGTCACGAGTATC
CIA1$_{Y527A}$ rvs	AAAATCCTTGAGGAGAGC
CIA1$_{F532A}$ fwd	CACGAGTATCgctCGCGGGGCGA
CIA1$_{F532A}$ rvs	ACATACCAAGGAGGCAAAATC
CIA1$_{R533A}$ fwd	GAGTATCTTTgccGGGGCGACCC
CIA1$_{R533A}$ rvs	GTGACATACCAAGGAGGC
W526A fwd	GGCGACTTCGGGAAGTGTGCTCTCCTCAAGGATTTTGCCTCCAgcGTATGTC ACGAGTATCTTTCGCGGGGCGACCCTCGGCGATGTGGT
W526A rvs	ACCACATCGCCGAGGGTCGCCCCGCGAAAGATACTCGTGACATACgcTGGAG GCAAAATCCTTGAGGAGAGCACACTTCCCGAAGTCGCC
Y527A fwd	GACTTCGGGAAGTGTGCTCTCCTCAAGGATTTTGCCTCCATGGgcTGTCACGA GTATCTTTCGCGGGGCGACCCTCGGCGATGTGGTTGT
Y527A rvs	ACAACCACATCGCCGAGGGTCGCCCCGCGAAAGATACTCGTGACAgcCCATGG AGGCAAAATCCTTGAGGAGAGCACACTTCCCGAAGTC
R533A fwd	CTCCTCAAGGATTTTGCCTCCATGGTATGTCACGAGTATCTTTgcCGGGG CGACCCTCGGCGATGTGGTTGTCTCGCCTAGTCCAGCGGG
R533A rvs	CCCGCTGGACTAGGCGAGACAACCACATCGCCGAGGGTCGCCCCGgcA AAGATACTCGTGACATACCATGGAGGCAAAATCCTTGAGGAG
CIA1 motif seq fwd	AGGTAGCTCTGCTGCTACGTCT
CIA1 motif seq rvs	CTTGTCAGATTCCGATTCACTC
TEV-HA$_3$ gBlock	GGTGGAGGTAGCGGTGGTGGAAGTGAAAATCTGTACTTCCAGGGAGGTA CCTACCCGTACGACGTCCCGGACTACGCTGGCTATCCCTATGATGTGCCCG ATTATGCGTATCCTTACGATGTTCCAGATTATGCCTGATAACCGCCCACAGA AGC
HA$_3$-mAID N-term gBlock	AAAATGGCATACCCATACGACGTCCCGGACTACGCTGGCTATCCCTATGATG TGCCCGATTATGCGTATCCTTACGATGTTCCAGATTATGCCGGCGGCGGCTCT GAGAAGAGCGCGTGTCCTAAAGATCCCGCTAAGCCGCCTGCCAAGGCCCA GGTGGTTGGCTGGCCCCCGGTTAGGAGTTACCGCAAGAACGTGATGGTCTC TTGCCAGAAGTCTAGTGGTGGCCCTGAGGCGGCGGCATTCGTTAAAGTCTC CATGGACGGAGCGCCGTACCTGCGAAAGATTGATTTGCGAATGTATAAAAG

	TGGTAGCGGTAGCAGTGGTGGAAGT
mAID-HA$_3$ C-term gBlock	GGTGGAGGTAGCGGTGGTGGAAGTGAGAAGAGCGCGTGTCCTAAAGATC<br>CCGCTAAGCCGCCTGCCAAGGCCCAGGTGGTTGGCTGGCCCCCGGTTAG<br>GAGTTACCGCAAGAACGTGATGGTCTCTTGCCAGAAGTCTAGTGGTGGCC<br>CTGAGGCGGCGGCATTCGTTAAAGTCTCCATGGACGGAGCGCCGTACCTG<br>CGAAAGATTGATTTGCGAATGTATAAAAGTGGCGGCGGCGGCTCTTACCCG<br>TACGACGTCCCGGACTACGCTGGCTATCCCTATGATGTGCCCGATTATGCGT<br>ATCCTTACGATGTTCCAGATTATGCCTGATAACCGCCCACAGAAGC
cMyc$_3$ gBlock	GGTGGAGGTAGCGGTGGTGGAAGTGAGCAGAAACTCATCTCTGAAG<br>AAGATCTGGAACAAAAGTTGATTTCAGAAGAAGATCTGGAACAGAA<br>GCTCATCTCTGAGGAAGATCTGGGCGCCTGATAACCGCCCACAGAAGC
CIA2$_{WT}$-Ty1 gBlock	ATGGACAATCCAAATCCTACGGTGTTGGAAGACGAGCCCGAGTCGGATCA<br>GGATGTCTTTGCCCCAGATGCGGACGGTTTTTACGTCACTTCGCGAGGGC<br>GGCTGGCGTCGCTCTTCAGCGCCTCGTCACGAAGCCCCAACTGGAGCTGT<br>TCCTTAGCCAATCCCAAGATTTTCTACTTCCCCCTTCTTCCCTCACATGCAAC<br>CCAACGAGCTTGTCACCAGCAGACCGACGCGTCGGGCATGGCGACTGCAG<br>AGAGCGCCGTTCCTCCTTCCCTTTTCAGAGAAGCAAGCGAGGCGCTAGCC<br>CCCGCAAAGGCCGACCTTCTGCGGAGCGAACAGGCTCTCCGTGCCATCAG<br>CGGAAACAGCGGAATCCACGGTTCTCTGTATCGTCGAAAACAAGATGTTCG<br>GCGACAAGTCGCGGACTACCTTTCGCTTTTGAAGACAGAGAAGCGTGTGG<br>CCAACCTCGTGGAAAAGAGCCGAGACGAAGAGAATGAGAGACGCGGGAG<br>AATACGATCCTCCCCAAGAGAGGCGAATCTGGGAGAGTTAGAGGGAGGCG<br>AACAAGGCAGCAGAGAGCGGAAGGACGAAGCCGAGAGACAACGAAGTG<br>CAGACGAGAAGGGTGAGGAGACCAGAGACGGCGCGGACGCGCTGGAGG<br>AAGCAGATCGAGCTGTGAAAAAGTGGATGTTGAAGAAACAAATGCGCAGA<br>CGCTTGTGGTCGAGGGAACCCTTCACTGTCGAGGAGCTCTACAATTATATCA<br>AACACATTCAAGACCCTGAACATCCATACTCGCTGGAGCAACTCGACGTAGT<br>CGCTCCGAAGAGGCTGACTGTGAGTGGTAGCTGTGAATTGTCTGACTCCAG<br>CGATCAAGAGAGTGTGGAGTCCGACTCCGACGCCTGCGACTCCAGAGGTC<br>ACCAGAGCGCGCTTCCTTCTTGCGGGCCCGGACGCGCGTCGGCTTATATTC<br>GGAGCCGGTGGGGAGGCCGGCGACGGCGCTTTCCGGAGAGTGGGTATGC<br>CTCGAATTCGCCACTGTTATCGGACTTGGAAAGCAACTCTAGCGTGAGCAG<br>TCTCTCCTCTGGAGAGAGTGATTTAGGTTCTTGCGACGATATCGAGTCCTTC<br>TTCTTCCCCGTTTCAGACAAGGAAAGCTGTCTGCACGCAGAGCAGGAGAAC<br>GAAGTGCCTTCGAGCCGAGGAAGCGGGCAGGACACTCGGGAGACGCGAG<br>ACAATACTCAAAACGTCTTTCCGGAGTCAGGGATGCATTTTTCTGTCCCCGA<br>TTTAGGTGCATGCGTTGGTGGCAGAGAATACCAAAAACCGTCGCTACGCTC<br>CTCTTTTTCTTCGACTGTTGCCTCTCCTTCCTGTTGTTCGACTTCCAAAGCAA<br>ACGCAACACTCGACGCCAGCGGAGGAGGTGACAGGGAAGAGACAGTGGG<br>AGGAGGAGAAGAGGAAATCTGCGCGTCGAGGAAACGACCAAGTGCACGC<br>GGTCTCCCGTCAGTGCAGCGAGAAGTCAGGGCCTTGGAACTCGCCTACAAA<br>AGTGGAAGAGGTCGACGCTGCAGTGTCAGTGTCTCCTTCCAGCCCACCATTC<br>CTCACTGCTCTCAGGCGACGTTAATTGGATTGCTGATTCTGGTGAAGCTCTTG<br>CGGTCAGCCCCTGTCTGGATGAAGAGCGAAATTCGAATCGCTGATGGCAAGC<br>ACGTGTCGTTCAAGACGATAAACCGCCAACTGAAAGACAAGGAACGAGTGA<br>GCGCCGCCATCGAAAATCCTGCTCTCTTGAAAGTCATCAATCGGGGTTTGCTA<br>GGAACGGACGCGTGGATTGACATCACCGAGCTTCTCGTCCTCCCTGAGCCTAG<br>GGGTGGAGGTAGCGGTGGTGGAAGTGAGGTGCATACCAATCAAGACCCTTTG<br>GATGAAGTCCATACCAATCAAGATCCTTTGGACGAGGTCCATACGAACCAGGA<br>CCCCTTGGACGGGGCCTGATAACCGCCCACAGAAGC

CIA2$_{C524A}$-Ty1 gBlock (residue 524 underlined)	ATGGACAATCCAAATCCTACGGTGTTGGAAGACGAGCCCGAGTCGGATCAGGA<br>TGTCTTTGCCCCAGATGCGGACGGTTTTTACGTCACTTCGCGAGGGCGGCTGGC<br>GTCGCTCTTCAGCGCCTCGTCACGAAGCCCCAACTGGAGCTGTTCCTTAGCCAA<br>TCCCAAGATTTTCTACTTCCCCCTTCTTCCCTCACATGCAACCCAACGAGCTTGTC<br>ACCAGCAGACCGACGCGTCGGGCATGGCGACTGCAGAGAGCGCCGTTCCTCCT<br>TCCCTTTTCAGAGAAGCAAGCGAGGCGCTAGCCCCCGCAAAGGCCGACCTTCT<br>GCGGAGCGAACAGGCTCTCCGTGCCATCAGCGGAAACAGCGGAATCCACGGTT<br>CTCTGTATCGTCGAAAACAAGATGTTCGGCGACAAGTCGCGGACTACCTTTCGC<br>TTTTGAAGACAGAGAAGCGTGTGGCCAACCTCGTGGAAAAGAGCCGAGACGA<br>AGAGAATGAGAGACGCGGGAGAATACGATCCTCCCCAAGAGAGGCGAATCTG<br>GGAGAGTTAGAGGGAGGCGAACAAGGCAGCAGAGAGCGGAAGGACGAAGCC<br>GAGAGACAACGAAGTGCAGACGAGAAGGGTGAGGAGACCAGAGACGGCGCG<br>GACGCGCTGGAGGAAGCAGATCGAGCTGTGAAAAAGTGGATGTTGAAGAAAC<br>AAATGCGCAGACGCTTGTGGTCGAGGGAACCCTTCACTGTCGAGGAGCTCTAC<br>AATTATATCAAACACATTCAAGACCCTGAACATCCATACTCGCTGGAGCAACTC<br>GACGTAGTCGCTCCGAAGAGGCTGACTGTGAGTGGTAGCTGTGAATTGTCTGA<br>CTCCAGCGATCAAGAGAGTGTGGAGTCCGACTCCGACGCCTGCGACTCCAGAG<br>GTCACCAGAGCGCGCTTCCTTCTTGCGGGCCCGGACGCGCGTCGGCTTATATTC<br>GGAGCCGGTGGGGAGGCCGGCGACGGCGCTTTCCGGAGAGTGGGTATGCCTC<br>GAATTCGCCACTGTTATCGGACTTGGAAAGCAACTCTAGCGTGAGCAGTCTCTC<br>CTCTGGAGAGAGTGATTTAGGTTCTTGCGACGATATCGAGTCCTTCTTCTTCCCC<br>GTTTCAGACAAGGAAAGCTGTCTGCACGCAGAGCAGGAGAACGAAGTGCCTTC<br>GAGCCGAGGAAGCGGGCAGGACACTCGGGAGACGCGAGACAATACTCAAAAC<br>GTCTTTCCGGAGTCAGGGATGCATTTTTCTGTCCCCGATTTAGGTGCATGCGTT<br>GGTGGCAGAGAATACCAAAAACCGTCGCTACGCTCCTCTTTTTCTTCGACTGTT<br>GCCTCTCCTTCCTGTTGTTCGACTTCCAAAGCAAACGCAACACTCGACGCCAGC<br>GGAGGAGGTGACAGGGAAGAGACAGTGGGAGGAGGAGAAGAGGAAATCTGC<br>GCGTCGAGGAAACGACCAAGTGCACGCGGTCTCCCGTCAGTGCAGCGAGAAG<br>TCAGGGCCTTGGAACTCGCCTACAAAAGTGGAAGAGGTCGACGCTGCAGTGTC<br>AGTGTCTCCTTCCAGCCCACCATTCCTCAC<u>GCC</u>TCTCAGGCGACGTTAATTGGAT<br>TGCTGATTCTGGTGAAGCTCTTGCGGTCAGCCCCTGTCTGGATGAAGAGCGAA<br>ATTCGAATCGCTGATGGCAAGCACGTGTCGTTCAAGACGATAAACCGCCAACT<br>GAAAGACAAGGAACGAGTGAGCGCCGCCATCGAAAATCCTGCTCTCTTGAAAG<br>TCATCAATCGGGGTTTGCTAGGAACGGACGCGTGGATTGACATCACCGAGCTT<br>CTCGTCCTCCCTGAGCCTAGGGGTGGAGGTAGCGGTGGTGGAAGTGAGGTGC<br>ATACCAATCAAGACCCTTTGGATGAAGTCCATACCAATCAAGATCCTTTGGACG<br>AGGTCCATACGAACCAGGACCCCTTGGACGGGGCCTGATAACCGCCCACAGAA<br>GC
mAID-cMyc$_3$ gBlock	GGTGGAGGTAGCGGTGGTGGAAGTGAGAAGAGCGCGTGTCCTAAAGATCCC<br>GCTAAGCCGCCTGCCAAGGCCCAGGTGGTTGGCTGGCCCCCGGTTAGGAGTT<br>ACCGCAAGAACGTGATGGTCTCTTGCCAGAAGTCTAGTGGTGGCCCTGAGGC<br>GGCGGCATTCGTTAAAGTCTCCATGGACGGAGCGCCGTACCTGCGAAAGATT<br>GATTTGCGAATGTATAAAGTGGCGGCGGCGGCTCTGAGCAGAAACTCATCTC<br>TGAAGAAGATCTGGAACAAAAGTTGATTTCAGAAGAAGATCTGGAACAGAAG<br>CTCATCTCTGAGGAAGATCTGGGCGCCTGATAACCGCCCACAGAAGC
Ty1 gBlock	GGTGGAGGTAGCGGTGGTGGAAGTGAGGTGCATACCAATCAAGACCCTT<br>TGGATGAAGTCCATACCAATCAAGATCCTTTGGACGAGGTCCATACGAAC<br>CAGGACCCCTTGGACGGGGCCTGATAACCGCCCACAGAAGC
CIA1$_{WT}$ gBlock	ATGCCTACCACTTGGCGTCTCAGGCGTGTGGCTAGATTTCGAGCACACGTG<br>GGGTGTGCATGGGGTGCCGCGTGGCGCCCGGATGGCGGCCTCCTTGCCAC<br>TTGCGGCTCCGACAGGCGCATTTGTCTTTGGGCTCCCGATAAGCTGTCGGC

	TGCTCCGGATGCGGGAGGATCGCTTGCAGGTGGTCGTGCAGAGCTTGCGG<br>ATGCGGCGCCACGGGGTACGGCTTCTTCCGCTCCGACAAACAGCCCAGAA<br>GGCAACGCAGCCAGGCAGCCAGAAAAACATGTAAAGGAGGCCCAGACA<br>GAGACCAGGCACCCCGAGAACGGCGAGAGGAACACCCTACAGACGGTGG<br>AAATCCGACGAAAGGGGATGGCCCACACAAGGAGAATCGAGGGGAAACA<br>CAAGGAACGCGAGAGCGAGGAAACCGCATGTCGCGCTCCGGTGGCGCAG<br>AGAAGACGCCTGGAGAGAACGGAGACTGCGCAAATCCGAGTATCCTTCG<br>TGGACGCTGATTAGCGTGATTGACGCGTCGGCGACCCACACACGCACAGTC<br>AGAAGTGTCTCCTTTTCCCCAGACGGCTTCTGGCTCGCGGCAGCCTCTTTCG<br>ATGCAACTGTCTCCGTTTGGTGCTCCCATGTAGCCGGCGTCTCTCGAACGCC<br>TTCTCGCTTTACGCAAGTTCAAGTCCTCGAAGGCCCTGAACACGAGGTCAAA<br>TGCGTCGCGTGGAGCCGAACGGGGCGGTACCTTGCCACTTGCAGTCGAGAT<br>AAAACGGTCTGGATTTTCGAGAGCAGCGCGGAAGACCGGGAAGAGGTCGA<br>GGCGTTTGCGGCTTCGCTGCGTCAAGGCCGGAGAACGCGAGGCTCTCGGCG<br>ACTCCAGCGGTCTCTCGGAGACGCTGCAGACGGAAGCAGTTGGCCCGCGGA<br>AACACTTGAAGAAGATGGCCGTTGGGATGCGGCGCAGAACGAGCGCGATTT<br>GTTTCTCGAAGAAAGACGCAGCGACTGGCCGCCGCCCTACGAGCTCGACGT<br>CAACATGGGAGACGACGGTTGCTTCTTCGTCGCTGCCGTTCTCAGTGGACAT<br>GCCCAGGATGTGAAAGCCGTGAGGTGGCATCCGCGAGAAGACCTTTGCATT<br>TCTGCTTCCTACGACGATACCTTTCGCGTCTGGGGTCTCCAAGGTGGAGCTG<br>GCGCTGAGTGGGGCCTCCTGCAAGTCGTGAAGGCTCACAGCTCAACGGTGT<br>TGTCGCTCGCGTTCGACCGCCTCGGCAGTCGCCTCGCCACCTGCTCCGACGA<br>CCGCCATCTGAAAATCTGGACTTGCCTCAACCCGCAGCTCGCGCATGCAACCG<br>GAGGTAGCTCTGCTGCTACGTCTCCTCGCTCGTTGCTCGCGTCTGCTTCGGCG<br>CCCCGTTCTCGGATGCTTTCTGAAGAGCTCACGGACCAGGCCGCTTGGGGAC<br>CTCTGCTGCCGCTTGGTGAAACGCATGCGGAGAAACGCGAAGAGGGAGAAG<br>AACTCGAGAGCGGGTCCAAAGCTTCTTGGACGTCGAGTTTTCCGACGAGCAC<br>CACGGCGACTTCGGGAAGTGTGCTCTCCTCAAGGATTTTGCCTCCTTGGTATG<br>TCACGAGTATCTTTCGCGGGGCGACCCTCGGCGATGTGGTTGTCTCGCCTAGT<br>CCAGCGGGTCCAGCAGACACGGAAACAGGGCCGGAAGAGACACATGGAGA<br>CAACGGAGACATCGGAGACACCCACGCAGAGACCGAGGACAGGGGCCGAG<br>CCCAAGGCTCCGGAGAGGTCCGCGGAGACGCAGCAGAAAGACGAAGCAAC<br>GATCGATGTCGACGCGAGAGAGAAGGAAAACGAGATGAGAGGCAATCTGAA<br>TGCTGTGAAGCTGAGTCCTTTGGAGCTTCCGAGAAAGAGGAAAGAACCCCA<br>CAAGCAGCTCGCAGAGAAGAAGCGGAGAAGAGTGGTTTCGTTGACAGCAG<br>AGGAGGGGAGGGAGAAGAGGGGACTGATTTGAGTGAATCGGAATCTGACA<br>AGGAGCGAAACGACAAAGAAGAAATACGACAACAACAACCAGATAAATGGA<br>GACCTGAAGCTGCGCTCTTGTCGGACATCCACACGAGACCCGTCTACTTTGTT<br>GACTGGCATGCGACACTCGATATCATCGTTACGGCATGTGGAGACAATGCTCT<br>GCGATTCTTCTCCGCCGAAGAAGACGAGGAAGGCGCCCGTTCCTGGGGGTT<br>GCTTCTTTCCAAGCCTGACGCGCACTACAGCGATATCAACTGCGCTGTTTGGA<br>ATCCTGTGACTCCTGCATGCAGTCGTCGATCCGAAGTGCTCCTCGGGAACGCG<br>AACGCGCACAAGACCGCAGCTCTGCTGGCGTCGGTCGATGACGACGGGAAG<br>ATGGCCATATGGAGTCTAGAGAGACGCCCTAGGGGTGGAGGTAGCGGTGGTG<br>GAAGTGAGGTGCATACCAATCAAGACCCTTTGGATGAAGTCCATACCAATCAA<br>GATCCTTTGGACGAGGTCCATACGAACCAGGACCCCTTGGACGGGGCCTGAT<br>AACCGCCCACAGAAGC
CIA1$_{ScCD1}$ gBlock	ATGCCTACCACTTGGCGTCTCAGGCGTGTGGCTAGATTTCGAGCACACGTGG<br>GGTGTGCATGGGGTGCCGCGTGGCGCCCGGATGGCGGCCTCCTTGCCACTT<br>GCGGCTCCGACAGGCGCATTTGTCTTTGGAGCGTGAAGTACGATGACTGGA<br>CGCTGATTAGCGTGATTGACGCGTCGGCGACCCACACACGCACAGTCAGAA

	GTGTCTCCTTTTCCCCAGACGGCTTCTGGCTCGCGGCAGCCTCTTTCGATGC<br>AACTGTCTCCGTTTGGTGCTCCCATGTAGCCGGCGTCTCTCGAACGCCTTCT<br>CGCTTTACGCAAGTTCAAGTCCTCGAAGGCCCTGAACACGAGGTCAAATG<br>CGTCGCGTGGAGCCGAACGGGGCGGTACCTTGCCACTTGCAGTCGAGATA<br>AAACGGTCTGGATTTTCGAGAGCAGCGCGGAAGACCGGGAAGAGGTCG<br>AGGCGTTTGCGGCTTCGCTGCGTCAAGGCCGGAGAACGCGAGGCTCTCG<br>GCGACTCCAGCGGTCTCTCGGAGACGCTGCAGACGGAAGCAGTTGGCCC<br>GCGGAAACACTTGAAGAAGATGGCCGTTGGGATGCGGCGCAGAACGAG<br>CGCGATTTGTTTCTCGAAGAAAGACGCAGCGACTGGCCGCCGCCCTACGA<br>GCTCGACGTCAACATGGGAGACGACGGTTGCTTCTTCGTCGCTGCCGTTC<br>TCAGTGGACATGCCCAGGATGTGAAAGCCGTGAGGTGGCATCCGCGAGAA<br>GACCTTTGCATTTCTGCTTCCTACGACGATACCTTTCGCGTCTGGGGTCTCC<br>AAGGTGGAGCTGGCGCTGAGTGGGGCCTCCTGCAAGTCGTGAAGGCTCA<br>CAGCTCAACGGTGTTGTCGCTCGCGTTCGACCGCCTCGGCAGTCGCCTCG<br>CCACCTGCTCCGACGACCGCCATCTGAAAATCTGGACTTGCCTCAACCCG<br>CAGCTCGCGCATGCAACCGGAGGTAGCTCTGCTGCTACGTCTCCTCGCTC<br>GTTGCTCGCGTCTGCTTCGGCGCCCCGTTCTCGGATGCTTTCTGAAGAGC<br>TCACGGACCAGGCCGCTTGGGGACCTCTGCTGCCGCTTGGTGAAACGCA<br>TGCGGAGAAACGCGAAGAGGGAGAAGAACTCGAGAGCGGGTCCAAAG<br>CTTCTTGGACGTCGAGTTTTCCGACGAGCACCACGGCGACTTCGGGAAG<br>TGTGCTCTCCTCAAGGATTTTGCCTCCTTGGTATGTCACGAGTATCTTTCG<br>CGGGGCGACCCTCGGCGATGTGGTTGTCTCGCCTAGTCCAGCGGGTCCA<br>GCAGACACGGAAACAGGGCCGGAAGAGACACATGGAGACAACGGAGA<br>CATCGGAGACACCCACGCAGAGACCGAGGACAGGGGCCGAGCCCAAG<br>GCTCCGGAGAGGTCCGCGGAGACGCAGCAGAAAGACGAAGCAACGAT<br>CGATGTCGACGCGAGAGAGAAGGAAAACGAGATGAGAGGCAATCTGA<br>ATGCTGTGAAGCTGAGTCCTTTGGAGCTTCCGAGAAAGAGGAAAGAAC<br>CCCACAAGCAGCTCGCAGAGAAGAAGCGGAGAAGAGTGGTTTCGTTG<br>ACAGCAGAGGAGGGGAGGGAGAAGAGGGGACTGATTTGAGTGAATC<br>GGAATCTGACAAGGAGCGAAACGACAAAGAAGAAATACGACAACAAC<br>AACCAGATAAATGGAGACCTGAAGCTGCGCTCTTGTCGGACATCCACAC<br>GAGACCCGTCTACTTTGTTGACTGGCATGCGACACTCGATATCATCGTTA<br>CGGCATGTGGAGACAATGCTCTGCGATTCTTCTCCGCCGAAGAAGACG<br>AGGAAGGCGCCCGTTCCTGGGGGTTGCTTCTTTCCAAGCCTGACGCGC<br>ACTACAGCGATATCAACTGCGCTGTTTGGAATCCTGTGACTCCTGCATGC<br>AGTCGTCGATCCGAAGTGCTCCTCGGGAACGCGAACGCGCACAAGAC<br>CGCAGCTCTGCTGGCGTCGGTCGATGACGACGGGAAGATGGCCATATG<br>GAGTCTAGAGAGACGCCCTAGGGGTGGAGGTAGCGGTGGTGGAAGT<br>GAGGTGCATACCAATCAAGACCCTTTGGATGAAGTCCATACCAATCAA<br>GATCCTTTGGACGAGGTCCATACGAACCAGGACCCCTTGGACGGGG<br>CCTGATAACCGCCCACAGAAGC
CIA1$_{\mathit{Sc}\mathrm{CD5}}$ gBlock	ATGCCTACCACTTGGCGTCTCAGGCGTGTGGCTAGATTTCGAGCACACGTGG<br>GGTGTGCATGGGGTGCCGCGTGGCGCCCGGATGGCGGCCTCCTTGCCACTT<br>GCGGCTCCGACAGGCGCATTTGTCTTTGGGCTCCCGATAAGCTGTCGGCTG<br>CTCCGGATGCGGGAGGATCGCTTGCAGGTGGTCGTGCAGAGCTTGCGGAT<br>GCGGCGCCACGGGGTACGGCTTCTTCCGCTCCGACAAACAGCCCAGAAGG<br>CAACGCAGCCAGGCAGCCAGAAAAACATGTAAAAGGAGGCCCAGACAGA<br>GACCAGGCACCCCGAGAACGGCGAGAGGAACACCCTACAGACGGTGGAA<br>ATCCGACGAAAGGGGATGGCCCACACAAGGAGAATCGAGGGGAAACACA<br>AGGAACGCGAGAGCGAGGAAACCGCATGTCGCGCTCCGGTGGCGCAGAG<br>AAGACGCCTGGAGAGAACGGGAGACTGCGCAAATCCGAGTATCCTTCGTG

	GACGCTGATTAGCGTGATTGACGCGTCGGCGACCCACACACGCACAGTCA<br>GAAGTGTCTCCTTTTCCCCAGACGGCTTCTGGCTCGCGGCAGCCTCTTTCG<br>ATGCAACTGTCTCCGTTTGGTGCTCCCATGTAGCCGGCGTCTCTCGAACGC<br>CTTCTCGCTTTACGCAAGTTCAAGTCCTCGAAGGCCCTGAACACGAGGTCA<br>AATGCGTCGCGTGGAGCCGAACGGGGCGGTACCTTGCCACTTGCAGTCGA<br>GATAAAACGGTCTGGATTTTCGAGAGCAGCGCGGAAGACCGGGAAGAG<br>GTCGAGGCGTTTGCGGCTTCGCTGCGTCAAGGCCGGAGAACGCGAGGCT<br>CTCGGCGACTCCAGCGGTCTCTCGGAGACGCTGCAGACGGAAGCAGTTG<br>GCCCGCGGAAACACTTGAAGAAGATGGCCGTTGGGATGCGGCGCAGAA<br>CGAGCGCGATTTGTTTCTCGAAGAAAGACGCAGCGACTGGCCGCCGCCC<br>TACGAGCTCGACGTCAACATGGGAGACGACGGTTGCTTCTTCGTCGCTGC<br>CGTTCTCAGTGGACATGCCCAGGATGTGAAAGCCGTGAGGTGGCATCCGC<br>GAGAAGACCTTTGCATTTCTGCTTCCTACGACGATACCTTTCGCGTCTGGG<br>GTCTCCAAGGTGGAGCTGGCGCTGAGTGGGGCCTCCTGCAAGTCGTGAA<br>GGCTCACAGCTCAACGGTGTTGTCGCTCGCGTTCGACCGCCTCGGCAGTC<br>GCCTCGCCACCTGCTCCGACGACCGCCATCTGAAAATCTGGAAGTATATG<br>GGTGATGACGAAGATGACCAGCAGGAATGGAGACCTGAAGCTGCGCTC<br>TTGTCGGACATCCACACGAGACCCGTCTACTTTGTTGACTGGCATGCGAC<br>ACTCGATATCATCGTTACGGCATGTGGAGACAATGCTCTGCGATTCTTCTC<br>CGCCGAAGAAGACGAGGAAGGCGCCCGTTCCTGGGGGTTGCTTCTTTCC<br>AAGCCTGACGCGCACTACAGCGATATCAACTGCGCTGTTTGGAATCCTGT<br>GACTCCTGCATGCAGTCGTCGATCCGAAGTGCTCCTCGGGAACGCGAAC<br>GCGCACAAGACCGCAGCTCTGCTGGCGTCGGTCGATGACGACGGGAAG<br>ATGGCCATATGGAGTCTAGAGAGACGCCCTAGGGGTGGAGGTAGCGGTG<br>GTGGAAGTGAGGTGCATACCAATCAAGACCCTTTGGATGAAGTCCATAC<br>CAATCAAGATCCTTTGGACGAGGTCCATACGAACCAGGACCCCTTGGAC<br>GGGGCCTGATAACCGCCCACAGAAGC
GFP$_{TgCD5}$ gBlock	AAAATGGCCGGAGTGTCGAAGGGTGAAGAGCTGTTCACCGGCGTGGT<br>GCCCATCCTCGTCGAGCTGGACGGAGATGTCAATGGACATAAATTCTCC<br>GTACGGGGAGAGGGCGAGGGCGACGCAACCAACGGTAAACTGACCCT<br>GAAGTTCATTTGCACCACGGGTAAACTGCCCGTACCCTGGCCCACCCT<br>GGTCACAACCTTGGGTGGCGGAGTTCAGTGTTTTTCGCGGTATCCAGA<br>TCACATGAAGCAGCATGACTTTTTCAAGAGCGCTATGCCCGAGGGTTA<br>CGTTCAAGAGAGGACCATCAGTTTCAAGGACGACGGCACCTACAAGA<br>CACGGGCTGAGGTGAAATTCGAGGGTGATACACTCGTGAATCGTATCG<br>AGCTGAAGGGCATAGATTTTAAGGAGGATGGCAATATCCTGGGCCACA<br>AGTTGGAGTATAACTTCAACAGTCACAATGTCTATATAACTGCCGATAAG<br>CAGAAGAATGGCATTAAAGCCAATTTCAAAATTCGTCACAATGTCGAA<br>GGCGGCGCAACTTGCCTCAACCCGCAGCTCGCGCATGCAACCGGAGG<br>TAGCTCTGCTGCTACGTCTCCTCGCTCGTTGCTCGCGTCTGCTTCGGCG<br>CCCCGTTCTCGGATGCTTTCTGAAGAGCTCACGGACCAGGCCGCTTGG<br>GGACCTCTGCTGCCGCTTGGTGAAACGCATGCGGAGAAACGCGAAGA<br>GGGAGAAGAACTCGAGAGCGGGTCCAAAGCTTCTTGGACGTCGAGT<br>TTTCCGACGAGCACCACGGCGACTTCGGGAAGTGTGCTCTCCTCAAG<br>GATTTTGCCTCCATGGTATGTCACGAGTATCTTTCGCGGGGCGACCCT<br>CGGCGATGTGGTTGTCTCGCCTAGTCCAGCGGGTCCAGCAGACACG<br>GAAACAGGGCCGGAAGAGACACATGGAGACAACGGAGACATCGGA<br>GACACCCACGCAGAGACCGAGGACAGGGGCCGAGCCCAAGGCTCC<br>GGAGAGGTCCGCGGAGACGCAGCAGAAAGACGAAGCAACGATCGA<br>TGTCGACGCGAGAGAGAAGGAAAACGAGATGAGAGGCAATCTGAAT<br>GCTGTGAAGCTGAGTCCTTTGGAGCTTCCGAGAAAGAGGAAAGAAC

	CCCACAAGCAGCTCGCAGAGAAGAAGCGGAGAAGAGTGGTTTCGTT<br>GACAGCAGAGGAGGGGAGGGAGAAGAGGGGACTGATTTGAGTGA<br>ATCGGAATCTGACAAGGAGCGAAACGACAAAGAAGAAATACGACAA<br>CAACAACCAGATAAAGCGGGAGGAGATGGAAGTGTCCAGTTGGCG<br>GATCACTACCAGCAGAATACGCCAATTGGTGACGGCCCCGTCCTTCT<br>GCCGGATAACCACTACCTCTCCACCCAGTCGGTGCTGAGCAAGGACC<br>CGAATGAAAAACGTGACCACATGGTTCTTCTTGAGTTCGTCACGGCT<br>GCGGGCATCACGTTGGGCATGGACGAACTGTACAAGGGTGGAGGT<br>AGCGGTGGTGGAAGT
*Dm*CIA1$_{WT}$ gBlock	ATGGGACGTTTAATTCTGGAACACACGCTGCAGGGCCACAAGGGGC<br>GCATCTGGGGAGTGGCATGGCATCCTAAGGGCAACGTATTCGCCTCG<br>TGCGGCGAGGACAAAGCCATCCGCATTTGGTCGCTGACCGGGAACA<br>CTTGGAGCACAAAGACCATCCTATCCGATGGCCACAAGCGCACTATT<br>CGCGAGATCCGATGGTCTCCATGCGGTCAGTACTTGGCCTCGGCCAG<br>CTTTGATGCCACCACGGCGATCTGGTCGAAATCCTCGGGAGAATTCG<br>AATGCAACGCAACATTGGAGGGTCACGAAAACGAGGTGAAGAGCG<br>TTAGCTGGTCGCGATCCGGCGGACTACTGGCCACCTGTTCGCGGGA<br>CAAGTCCGTGTGGATCTGGGAGGTTGCCGGCGATGATGAGTTCGAG<br>TGCGCCGCCGTTTTGAACCCGCACACACAGGACGTGAAGAGAGTG<br>GTGTGGCATCCAACCAAGGACATTTTGGCCTCCGCCTCGTACGACA<br>ATACCATAAAAATGTTCGCAGAGGAGCCCATAGACAACGACTGGGA<br>TTGCACAGCCACTCTTACTTCACACACAAGCACGGTTTGGGGCATT<br>GACTTCGATGCTGATGGCGAGCGCCTGGTTTCCTGTAGCGATGACAC<br>CACAATAAAGATTTGGAGGGCCTACCATCCCGGAAATACAGCCGGA<br>GTGGCTACGCCCGACCAGCAAACCGTGTGGAAGTGCGTGTGCACAG<br>TGTCCGGGCAGCACTCGCGGGCTATCTACGACGTGTCCTGGTGCAA<br>GCTTACAGGTCTGATAGCGACTGCCTGTGGCGACGACGGCATTCGT<br>ATCTTTAAGGAGAGCAGCGACTCCAAGCCAGACGAACCCACGTTCG<br>AGCAGATAACCGCGGAGGAAGGCGCTCACGATCAGGACGTAAACT<br>CGGTGCAGTGGAATCCTGTGGTGGCCGGGCAACTGATATCATGCAG<br>CGATGATGGCACCATTAAAATTTGGAAAGTGACCGAGCCTAGGGGT<br>GGAGGTAGCGGTGGTGGAAGTGAGGTGCATACCAATCAAGACCC<br>TTTGGATGAAGTCCATACCAATCAAGATCCTTTGGACGAGGTCCA<br>TACGAACCAGGACCCCTTGGACGGGGCCTGATAACCGCCCACAGA<br>AGC
*Dm*CIA1$_{\mathit{Tg}\mathrm{CD5}}$ gBlock	ATGGGACGTTTAATTCTGGAACACACGCTGCAGGGCCACAAGGGGCG<br>CATCTGGGGAGTGGCATGGCATCCTAAGGGCAACGTATTCGCCTCGTGC<br>GGCGAGGACAAAGCCATCCGCATTTGGTCGCTGACCGGGAACACTTGG<br>AGCACAAAGACCATCCTATCCGATGGCCACAAGCGCACTATTCGCGAGA<br>TCCGATGGTCTCCATGCGGTCAGTACTTGGCCTCGGCCAGCTTTGATGC<br>CACCACGGCGATCTGGTCGAAATCCTCGGGAGAATTCGAATGCAACGCA<br>ACATTGGAGGGTCACGAAAACGAGGTGAAGAGCGTTAGCTGGTCGCG<br>ATCCGGCGGACTACTGGCCACCTGTTCGCGGGACAAGTCCGTGTGGAT<br>CTGGGAGGTTGCCGGCGATGATGAGTTCGAGTGCGCCGCCGTTTTGAA<br>CCCGCACACACAGGACGTGAAGAGAGTGGTGTGGCATCCAACCAAGG<br>ACATTTTGGCCTCCGCCTCGTACGACAATACCATAAAAATGTTCGCAGA<br>GGAGCCCATAGACAACGACTGGGATTGCACAGCCACTCTTACTTCACA<br>CACAAGCACGGTTTGGGGCATTGACTTCGATGCTGATGGCGAGCGCCT<br>GGTTTCCTGTAGCGATGACACCACAATAAAGATTTGGACTTGCCTCAAC<br>CCGCAGCTCGCGCATGCAACCGGAGGTAGCTCTGCTGCTACGTCTCCT<br>CGCTCGTTGCTCGCGTCTGCTTCGGCGCCCCGTTCTCGGATGCTTTCTG

	AAGAGCTCACGGACCAGGCCGCTTGGGGACCTCTGCTGCCGCTTGGT<br>GAAACGCATGCGGAGAAACGCGAAGAGGGAGAAGAACTCGAGAGC<br>GGGTCCAAAGCTTCTTGGACGTCGAGTTTTCCGACGAGCACCACGGC<br>GACTTCGGGAAGTGTGCTCTCCTCAAGGATTTTGCCTCCTTGGTATGTC<br>ACGAGTATCTTTCGCGGGGCGACCCTCGGCGATGTGGTTGTCTCGCCT<br>AGTCCAGCGGGTCCAGCAGACACGGAAACAGGGCCGGAAGAGACAC<br>ATGGAGACAACGGAGACATCGGAGACACCCACGCAGAGACCGAGGA<br>CAGGGGCCGAGCCCAAGGCTCCGGAGAGGTCCGCGGAGACGCAGC<br>AGAAAGACGAAGCAACGATCGATGTCGACGCGAGAGAGAAGGAAAA<br>CGAGATGAGAGGCAATCTGAATGCTGTGAAGCTGAGTCCTTTGGAGC<br>TTCCGAGAAAGAGGAAAGAACCCCACAAGCAGCTCGCAGAGAAGAA<br>GCGGAGAAGAGTGGTTTCGTTGACAGCAGAGGAGGGGAGGGAGA<br>AGAGGGGACTGATTTGAGTGAATCGGAATCTGACAAGGAGCGAAAC<br>GACAAAGAAGAAATACGACAACAACAACCAGATAAATGGAAGTGCGT<br>GTGCACAGTGTCCGGGCAGCACTCGCGGGCTATCTACGACGTGTCCT<br>GGTGCAAGCTTACAGGTCTGATAGCGACTGCCTGTGGCGACGACGGC<br>ATTCGTATCTTTAAGGAGAGCAGCGACTCCAAGCCAGACGAACCCACG<br>TTCGAGCAGATAACCGCGGAGGAAGGCGCTCACGATCAGGACGTAA<br>ACTCGGTGCAGTGGAATCCTGTGGTGGCCGGGCAACTGATATCATGCA<br>GCGATGATGGCACCATTAAAATTTGGAAAGTGACCGAGCCTAGGGGT<br>GGAGGTAGCGGTGGTGGAAGTGAGGTGCATACCAATCAAGACCCTTT<br>GGATGAAGTCCATACCAATCAAGATCCTTTGGACGAGGTCCATACGAAC<br>CAGGACCCCTTGGACGGGGCCTGATAACCGCCCACAGAAGC
*Tg*CIA1$_{CD5\text{-}to\text{-}CD1}$<br>gBlock	AAAATGCCTACCACTTGGCGTCTCAGGCGTGTGGCTAGATTTCGAGCACAC<br>GTGGGGTGTGCATGGGGTGCCGCGTGGCGCCCGGATGGCGGCCTCCTTGC<br>CACTTGCGGCTCCGACAGGCGCATTTGTCTTTGGACTTGCCTCAACCCGCA<br>GCTCGCGCATGCAACCGGAGGTAGCTCTGCTGCTACGTCTCCTCGCTCGTTG<br>CTCGCGTCTGCTTCGGCGCCCCGTTCTCGGATGCTTTCTGAAGAGCTCACG<br>GACCAGGCCGCTTGGGGACCTCTGCTGCCGCTTGGTGAAACGCATGCGGA<br>GAAACGCGAAGAGGGAGAAGAACTCGAGAGCGGGTCCAAAGCTTCTTG<br>GACGTCGAGTTTTCCGACGAGCACCACGGCGACTTCGGGAAGTGTGCTCT<br>CCTCAAGGATTTTGCCTCCATGGTATGTCACGAGTATCTTTCGCGGGGCGA<br>CCCTCGGCGATGTGGTTGTCTCGCCTAGTCCAGCGGGTCCAGCAGACACG<br>GAAACAGGGCCGGAAGAGACACATGGAGACAACGGAGACATCGGAGAC<br>ACCCACGCAGAGACCGAGGACAGGGGCCGAGCCCAAGGCTCCGGAGAG<br>GTCCGCGGAGACGCAGCAGAAAGACGAAGCAACGATCGATGTCGACGCG<br>AGAGAGAAGGAAAACGAGATGAGAGGCAATCTGAATGCTGTGAAGCTGAG<br>TCCTTTGGAGCTTCCGAGAAAGAGGAAAGAACCCCACAAGCAGCTCGCAG<br>AGAAGAAGCGGAGAAGAGTGGTTTCGTTGACAGCAGAGGAGGGGAGGGA<br>GAAGAGGGGACTGATTTGAGTGAATCGGAATCTGACAAGGAGCGAAACGA<br>CAAAGAAGAAATACGACAACAACAACCAGATAAATGGACGCTGATTAGCGT<br>GATTGACGCGTCGGCGACCCACACACGCACAGTCAGAAGTGTCTCCTTTTCC<br>CCAGACGGCTTCTGGCTCGCGGCAGCCTCTTTCGATGCAACTGTCTCCGTTT<br>GGTGCTCCCATGTAGCCGGCGTCTCTCGAACGCCTTCTCGCTTTACGCAAGTT<br>CAAGTCCTCGAAGGCCCTGAACACGAGGTCAAATGCGTCGCGTGGAGCCGA<br>ACGGGGCGGTACCTTGCCACTTGCAGTCGAGATAAAACGGTCTGGATTTTCG<br>AGAGCAGCGCGGAAGACCGGGAAGAGGTCGAGGCGTTTGCGGCTTCGCTG<br>CGTCAAGGCCGGAGAACGCGAGGCTCTCGGCGACTCCAGCGGTCTCTCGGA<br>GACGCTGCAGACGGAAGCAGTTGGCCCGCGGAAACACTTGAAGAAGATGG<br>CCGTTGGGATGCGGCGCAGAACGAGCGCGATTTGTTTCTCGAAGAAAGACG<br>CAGCGACTGGCCGCCGCCCTACGAGCTCGACGTCAACATGGGAGACGACGG

	TTGCTTCTTCGTCGCTGCCGTTCTCAGTGGACATGCCCAGGATGTGAAAGCCG<br>TGAGGTGGCATCCGCGAGAAGACCTTTGCATTTCTGCTTCCTACGACGATACC<br>TTTCGCGTCTGGGGTCTCCAAGGTGGAGCTGGCGCTGAGTGGGGCCTCCTGC<br>AAGTCGTGAAGGCTCACAGCTCAACGGTGTTGTCGCTCGCGTTCGACCGCCT<br>CGGCAGTCGCCTCGCCACCTGCTCCGACGACCGCCATCTGAAAATCTGGAAGT<br>ATATGGGTGATGACGAAGATGACCAGCAGGAATGGAGACCTGAAGCTGCGCT<br>CTTGTCGGACATCCACACGAGACCCGTCTACTTTGTTGACTGGCATGCGACAC<br>TCGATATCATCGTTACGGCATGTGGAGACAATGCTCTGCGATTCTTCTCCGCCG<br>AAGAAGACGAGGAAGGCGCCCGTTCCTGGGGGTTGCTTCTTTCCAAGCCTG<br>ACGCGCACTACAGCGATATCAATTGCGCTGTTTGGAATCCTGTGACTCCTGCA<br>TGCAGTCGTCGATCCGAAGTGCTCCTCGGGAACGCGAACGCGCACAAGACC<br>GCAGCTCTGCTGGCGTCGGTCGATGACGACGGGAAGATGGCCATATGGAGT<br>CTAGAGAGACGC
smFP-cMyc gBlock	GGTGGAGGTAGCGGTGGTGGAAGTGAGCAAAAACTGATATCGGAGGAAGAC<br>TTGGCAGAGCAGAAACTGATTTCGGAAGAGGATCTTGCCGAACAAAAACTGA<br>TCAGTGAGGAAGACCTTGCCGGAGTGTCGAAGGGTGAAGAGCTGTTCACCG<br>GCGTGGTGCCCATCCTCGTCGAGCTGGACGGAGATGTCAATGGACATAAATTC<br>TCCGTACGGGGAGAGGGCGAGGGCGACGCAACCAACGGTAAACTGACCCTG<br>AAGTTCATTTGCACCACGGGTAAACTGCCCGTACCCTGGCCCACCCTGGTCAC<br>AACCTTGGGTGGCGGAGTTCAGTGTTTTTCGCGGTATCCAGATCACATGAAGC<br>AGCATGACTTTTTCAAGAGCGCTATGCCCGAGGGTTACGTTCAAGAGAGGAC<br>CATCAGTTTCAAGGACGACGGCACCTACAAGACACGGGCTGAGGTGAAATTC<br>GAGGGTGATACACTCGTGAATCGTATCGAGCTGAAGGGCATAGATTTTAAGGA<br>GGATGGCAATATCCTGGGCCACAAGTTGGAGTATAACTTCAACAGTCACAATG<br>TCTATATAACTGCCGATAAGCAGAAGAATGGCATTAAAGCCAATTTCAAAATTC<br>GTCACAATGTCGAAGGCGGCGCAGAACAGAAGCTCATCAGTGAAGAGGATCT<br>GGCCGCAGAGCAGAAACTCATTAGCGAGGAGGACTTGGGCGGAGGCGGCG<br>AGCAGAAGCTGATATCGGAGGAAGACCTGGCGGCCGAGCAAAAGCTCATCTC<br>GGAGGAGGATCTCGCGGGAGGAGATGGAAGTGTCCAGTTGGCGGATCACTAC<br>CAGCAGAATACGCCAATTGGTGACGGCCCCGTCCTTCTGCCGGATAACCACTAC<br>CTCTCCACCCAGTCGGTGCTGAGCAAGGACCCGAATGAAAAACGTGACCACAT<br>GGTTCTTCTTGAGTTCGTCACGGCTGCGGGCATCACGTTGGGCATGGACGAAC<br>TGTACAAGGGCGCTGAACAGAAACTTATCTCCGAGGAGGATCTGGCGGAACA<br>GAAGCTGATCAGCGAAGAGGACCTGGCGGAGCAGAAACTTATCAGCGAAGA<br>GGATCTGGCCTGATAACCGCCCACAGAAGC
CIA1 $_{VbCD5}$ gBlock	ATGCCTACCACTTGGCGTCTCAGGCGTGTGGCTAGATTTCGAGCACACGTGG<br>GGTGTGCATGGGGTGCCGCGTGGCGCCCGGATGGCGGCCTCCTTGCCACTT<br>GCGGCTCCGACAGGCGCATTTGTCTTTGGGCTCCCGATAAGCTGTCGGCTGC<br>TCCGGATGCGGGAGGATCGCTTGCAGGTGGTCGTGCAGAGCTTGCGGATGC<br>GGCGCCACGGGGTACGGCTTCTTCCGCTCCGACAAACAGCCCAGAAGGCAA<br>CGCAGCCAGGCAGCCAGAAAAACATGTAAAAGGAGGCCCAGACAGAGACC<br>AGGCACCCCGAGAACGGCGAGAGGAACACCCTACAGACGGTGGAAATCCG<br>ACGAAAGGGGATGGCCCACACAAGGAGAATCGAGGGGAAACACAAGGAAC<br>GCGAGAGCGAGGAAACCGCATGTCGCGCTCCGGTGGCGCAGAGAAGACGC<br>CTGGAGAGAACGGGAGACTGCGCAAATCCGAGTATCCTTCGTGGACGCTGA<br>TTAGCGTGATTGACGCGTCGGCGACCCACACACGCACAGTCAGAAGTGTCTC<br>CTTTTCCCCAGACGGCTTCTGGCTCGCGGCAGCCTCTTTCGATGCAACTGTCT<br>CCGTTTGGTGCTCCCATGTAGCCGGCGTCTCTCGAACGCCTTCTCGCTTTACG<br>CAAGTTCAAGTCCTCGAAGGCCCTGAACACGAGGTCAAATGCGTCGCGTGG<br>AGCCGAACGGGGCGGTACCTTGCCACTTGCAGTCGAGATAAAACGGTCTGG<br>ATTTTCGAGAGCAGCGCGGAAGACCGGGAAGAGGTCGAGGCGTTTGCGGCT

	TCGCTGCGTCAAGGCCGGAGAACGCGAGGCTCTCGGCGACTCCAGCGGTCT<br>CTCGGAGACGCTGCAGACGGAAGCAGTTGGCCCGCGGAAACACTTGAAGAA<br>GATGGCCGTTGGGATGCGGCGCAGAACGAGCGCGATTTGTTTCTCGAAGAA<br>AGACGCAGCGACTGGCCGCCGCCCTACGAGCTCGACGTCAACATGGGAGA<br>CGACGGTTGCTTCTTCGTCGCTGCCGTTCTCAGTGGACATGCCCAGGATGTG<br>AAAGCCGTGAGGTGGCATCCGCGAGAAGACCTTTGCATTTCTGCTTCCTACG<br>ACGATACCTTTCGCGTCTGGGGTCTCCAAGGTGGAGCTGGCGCTGAGTGGG<br>GCCTCCTGCAAGTCGTGAAGGCTCACAGCTCAACGGTGTTGTCGCTCGCGTT<br>CGACCGCCTCGGCAGTCGCCTCGCCACCTGCTCCGACGACCGCCATCTGAAA<br>ATCTGGACACGTCTGCCTCCCCGGCGGCCCCCAGCGGCTGCCCCCCAGCAG<br>CAGACTCTCCCCGACGGCAAAGGGAAGCTGCCCCCATGGTACCTGTCCGGT<br>GTCTTCAGATCCGCCGTCACATCATCCTCTCACACAGAACAACTTGACCAATA<br>CCTGCAACAACAGCAACAGCAGCAGCCATCTGAGCAGGAGCCACAGGAGC<br>AGCAAGAGCAGGCCAACGGTGTGTTGGGTGAAGAGGAGAGGGTCGAGAT<br>GGATGTGAGTGGGTGGAGACCTGAAGCTGCGCTCTTGTCGGACATCCACAC<br>GAGACCCGTCTACTTTGTTGACTGGCATGCGACACTCGATATCATCGTTACG<br>GCATGTGGAGACAATGCTCTGCGATTCTTCTCCGCCGAAGAAGACGAGGAA<br>GGCGCCCGTTCCTGGGGGTTGCTTCTTTCCAAGCCTGACGCGCACTACAGCG<br>ATATCAACTGCGCTGTTTGGAATCCTGTGACTCCTGCATGCAGTCGTCGATC<br>CGAAGTGCTCCTCGGGAACGCGAACGCGCACAAGACCGCAGCTCTGCTGGC<br>GTCGGTCGATGACGACGGGAAGATGGCCATATGGAGTCTAGAGAGACGCC<br>CTAGGGGTGGAGGTAGCGGTGGTGGAAGTGAGGTGCATACCAATCAAGAC<br>CCTTTGGATGAAGTCCATACCAATCAAGATCCTTTGGACGAGGTCCATACGA<br>ACCAGGACCCCTTGGACGGGGCCTGATAACCGCCCACAGAAGC
CIA1 $_{\mathit{Sm}\mathrm{CD5}}$ gBlock	ATGCCTACCACTTGGCGTCTCAGGCGTGTGGCTAGATTTCGAGCA<br>CACGTGGGGTGTGCATGGGGTGCCGCGTGGCGCCCGGATGGCGG<br>CCTCCTTGCCACTTGCGGCTCCGACAGGCGCATTTGTCTTTGGGC<br>TCCCGATAAGCTGTCGGCTGCTCCGGATGCGGGAGGATCGCTTG<br>CAGGTGGTCGTGCAGAGCTTGCGGATGCGGCGCCACGGGGTACG<br>GCTTCTTCCGCTCCGACAAACAGCCCAGAAGGCAACGCAGCCAG<br>GCAGCCAGAAAAACATGTAAAAGGAGGCCCAGACAGAGACCAG<br>GCACCCCGAGAACGGCGAGAGGAACACCCTACAGACGGTGGAA<br>ATCCGACGAAAGGGGATGGCCCACACAAGGAGAATCGAGGGGA<br>AACACAAGGAACGCGAGAGCGAGGAAACCGCATGTCGCGCTCC<br>GGTGGCGCAGAGAAGACGCCTGGAGAGAACGGGAGACTGCGCA<br>AATCCGAGTATCCTTCGTGGACGCTGATTAGCGTGATTGACGCG<br>TCGGCGACCCACACACGCACAGTCAGAAGTGTCTCCTTTTCCCC<br>AGACGGCTTCTGGCTCGCGGCAGCCTCTTTCGATGCAACTGTCTC<br>CGTTTGGTGCTCCCATGTAGCCGGCGTCTCTCGAACGCCTTCTCG<br>CTTTACGCAAGTTCAAGTCCTCGAAGGCCCTGAACACGAGGTCA<br>AATGCGTCGCGTGGAGCCGAACGGGGCGGTACCTTGCCACTTGC<br>AGTCGAGATAAAACGGTCTGGATTTTCGAGAGCAGCGCGGAAGA<br>CCGGGAAGAGGTCGAGGCGTTTGCGGCTTCGCTGCGTCAAGGCC<br>GGAGAACGCGAGGCTCTCGGCGACTCCAGCGGTCTCTCGGAGAC<br>GCTGCAGACGGAAGCAGTTGGCCCGCGGAAACACTTGAAGAAGA<br>TGGCCGTTGGGATGCGGCGCAGAACGAGCGCGATTTGTTTCTCGA<br>AGAAAGACGCAGCGACTGGCCGCCGCCCTACGAGCTCGACGTCA<br>ACATGGGAGACGACGGTTGCTTCTTCGTCGCTGCCGTTCTCAGTG<br>GACATGCCCAGGATGTGAAAGCCGTGAGGTGGCATCCGCGAGAA<br>GACCTTTGCATTTCTGCTTCCTACGACGATACCTTTCGCGTCTGGG<br>GTCTCCAAGGTGGAGCTGGCGCTGAGTGGGGCCTCCTGCAAGTC

	GTGAAGGCTCACAGCTCAACGGTGTTGTCGCTCGCGTTCGACCGC<br>CTCGGCAGTCGCCTCGCCACCTGCTCCGACGACCGCCATCTGAAA<br>ATCTGGGCTCCCTCTGCAACCCTTCCAGCAGGCTTCAGGCCCGTG<br>GAAAGCCACAGCACAGAGCCTGTCAAGCAGGCTGAACAAGTTGA<br>AGTCACAACAGCAAGTGCCGAAGACTTGCTCTTCGCCCAGTCTGT<br>CAGGCCACTTTTTCGAGGAGCTTTGGTCAAACCGGCGTCTTCGTCT<br>GCAGCACCCCCGCAAGCTGTCACACAGCCAGTGGCCAACGGCAGA<br>AGTGAGGCTGCTACGACGTCAGGCCACAAAGCTTGCAGATCAGCG<br>CCCACTGACGCCTCCTGCGGCTGGAGACCTGAAGCTGCGCTCTTG<br>TCGGACATCCACACGAGACCCGTCTACTTTGTTGACTGGCATGCGA<br>CACTCGATATCATCGTTACGGCATGTGGAGACAATGCTCTGCGATT<br>CTTCTCCGCCGAAGAAGACGAGGAAGGCGCCCGTTCCTGGGGGTT<br>GCTTCTTTCCAAGCCTGACGCGCACTACAGCGATATCAACTGCGCT<br>GTTTGGAATCCTGTGACTCCTGCATGCAGTCGTCGATCCGAAGTGC<br>TCCTCGGGAACGCGAACGCGCACAAGACCGCAGCTCTGCTGGCGT<br>CGGTCGATGACGACGGGAAGATGGCCATATGGAGTCTAGAGAGA<br>CGCCCTAGGGGTGGAGGTAGCGGTGGTGGAAGTGAGGTGCATAC<br>CAATCAAGACCCTTTGGATGAAGTCCATACCAATCAAGATCCTTT<br>GGACGAGGTCCATACGAACCAGGACCCCTTGGACGGGGCCTGAT<br>AACCGCCCACAGAAGC
