# Supplementary material for: A novel targeting domain directs essential components of the cytosolic iron–sulfur cluster assembly pathway to the mitochondrion of Toxoplasma parasites
Source: PLoS Biol. 2025 Nov 25;23(11):e3003520. doi: 10.1371/journal.pbio.3003520 (PMC12674569; doi:10.1371/journal.pbio.3003520)
Supplement: S1 Raw images — Refer to the Figure legends in the manuscript for details on each figure. (PDF) [file pbio.3003520.s015.pdf]

Fig1

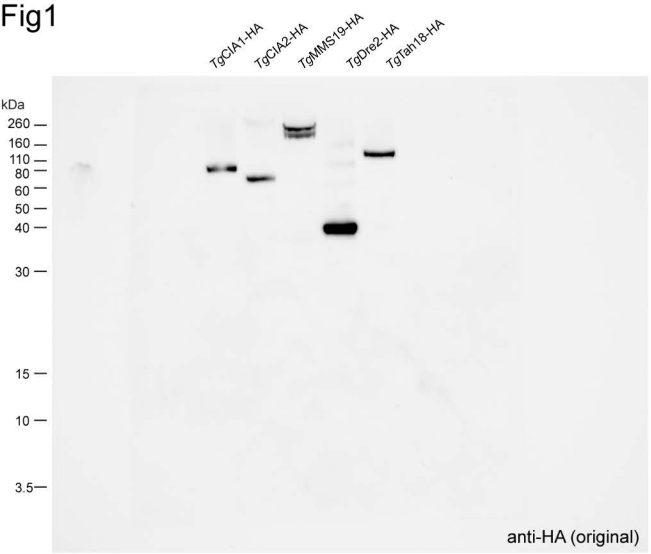

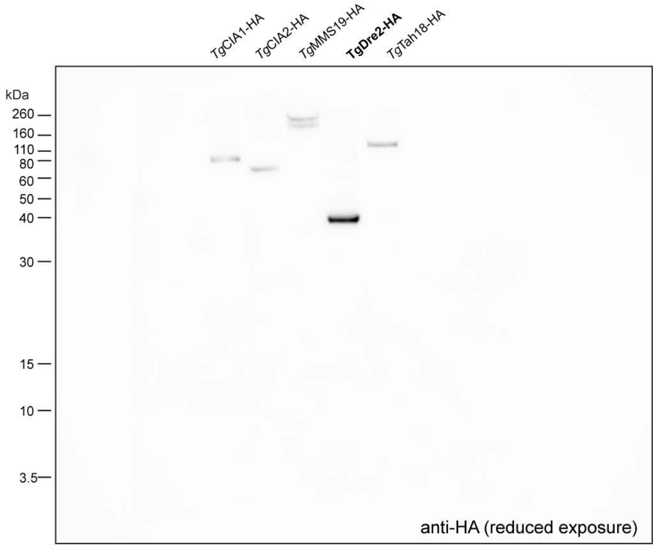

Fig1C

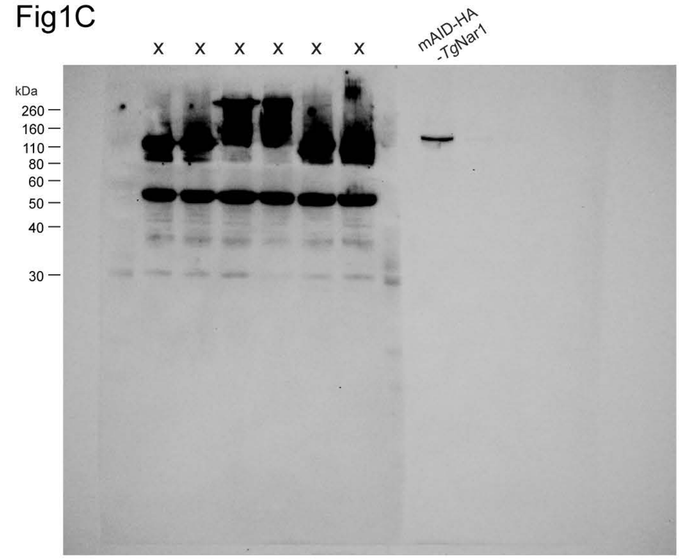

Fig2

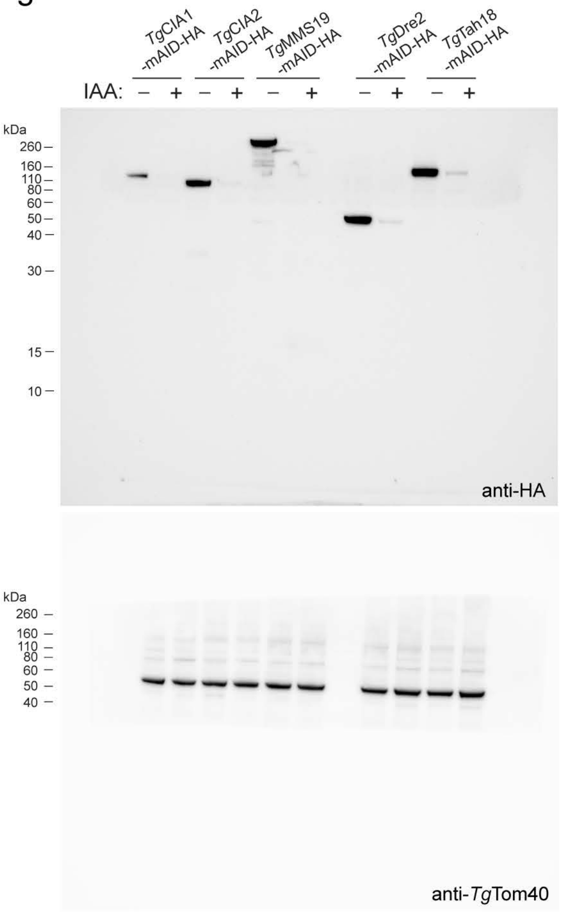

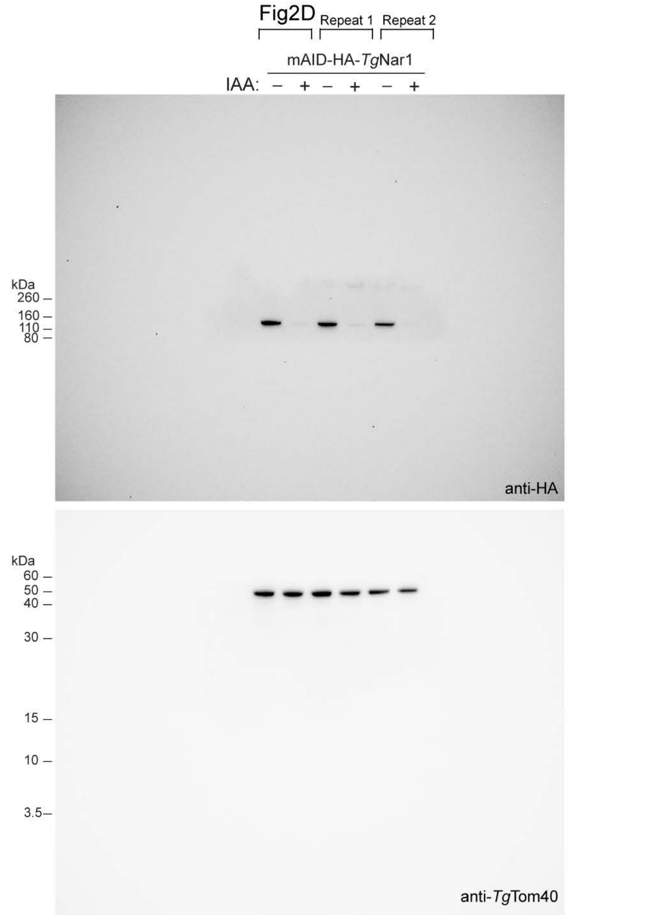

Fig3C Repeat 1 Repeat 2

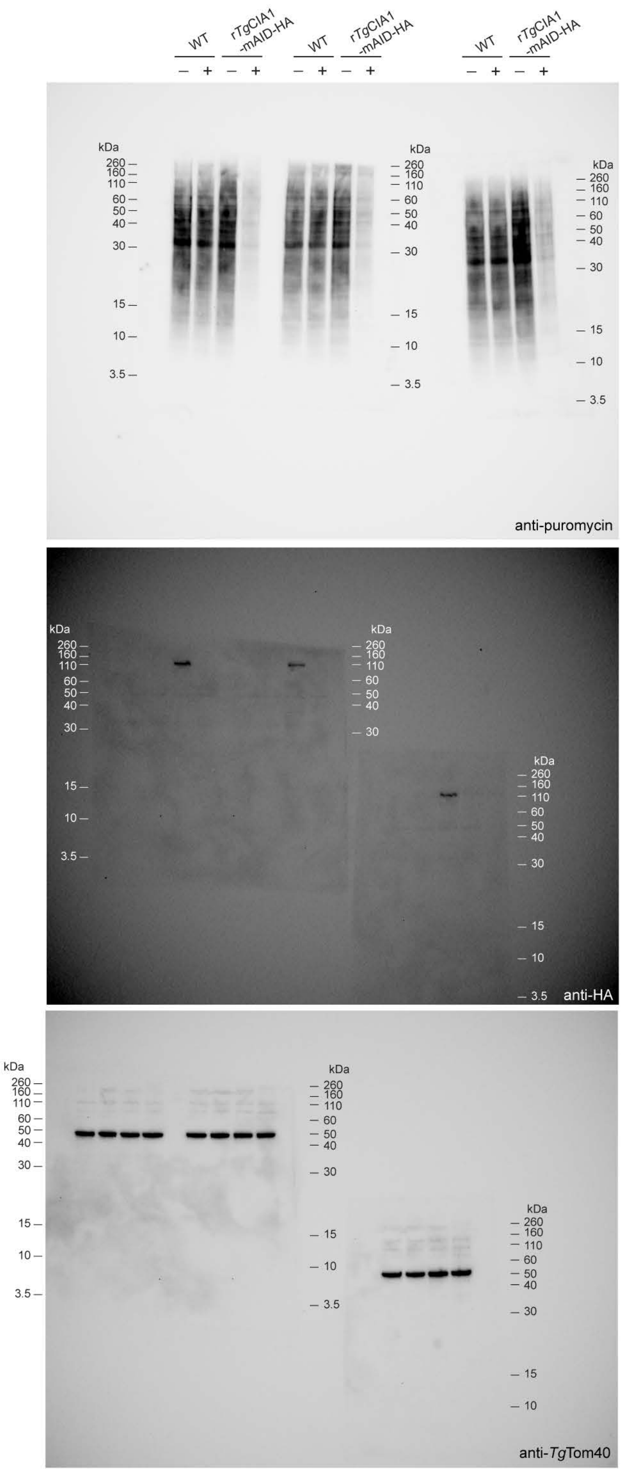

Fig3F

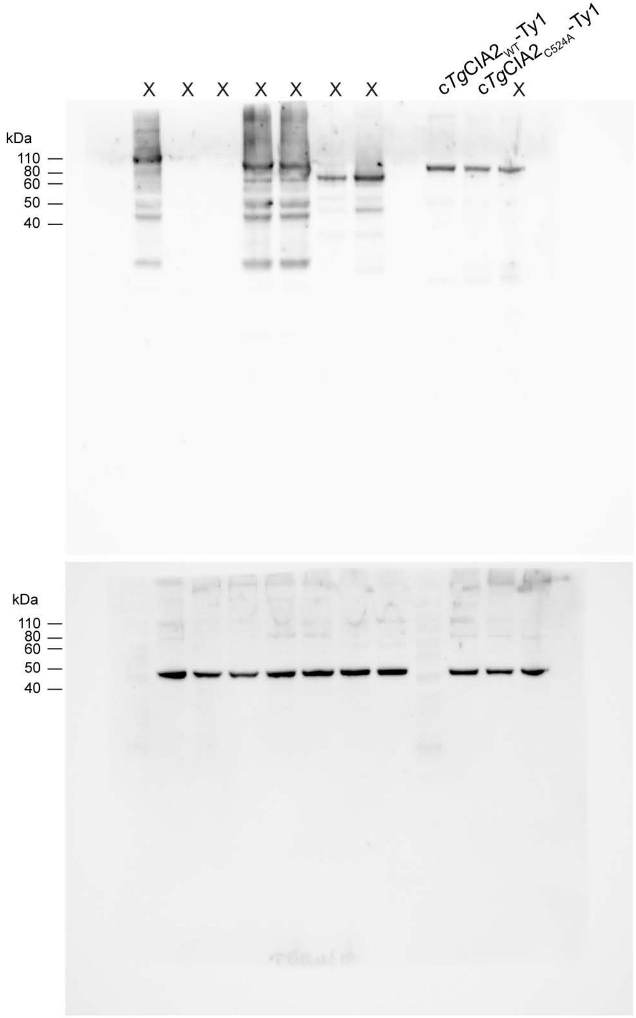

Fig3A

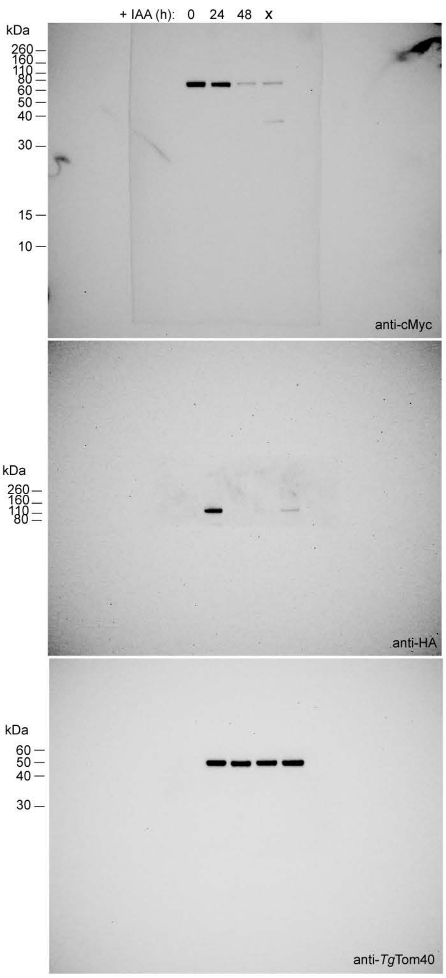

Repeat 1

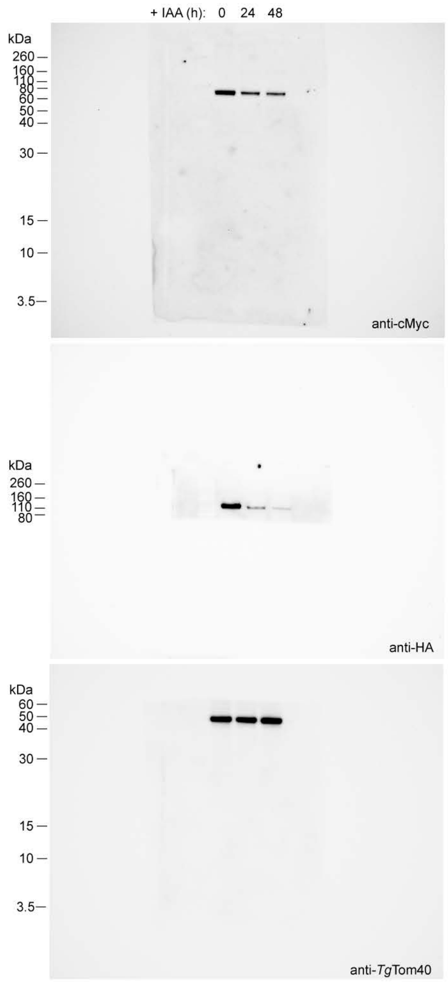

Repeat 2

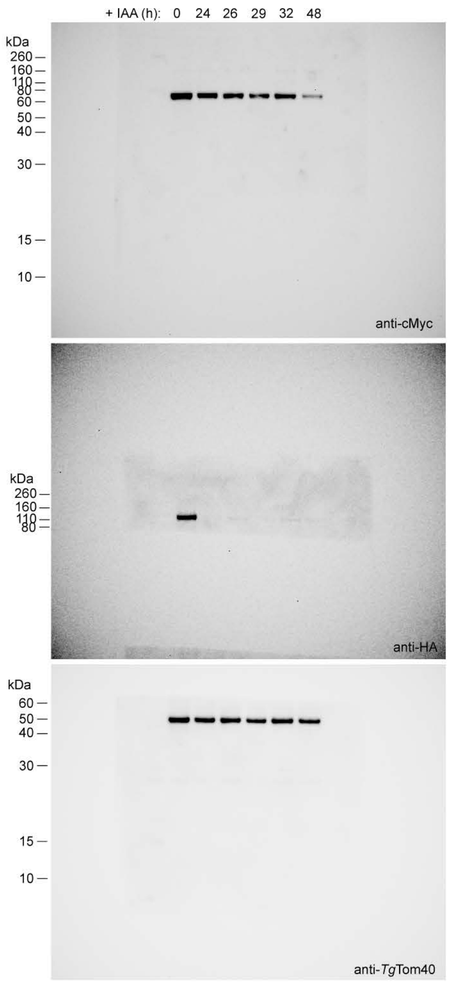

Repeat 3

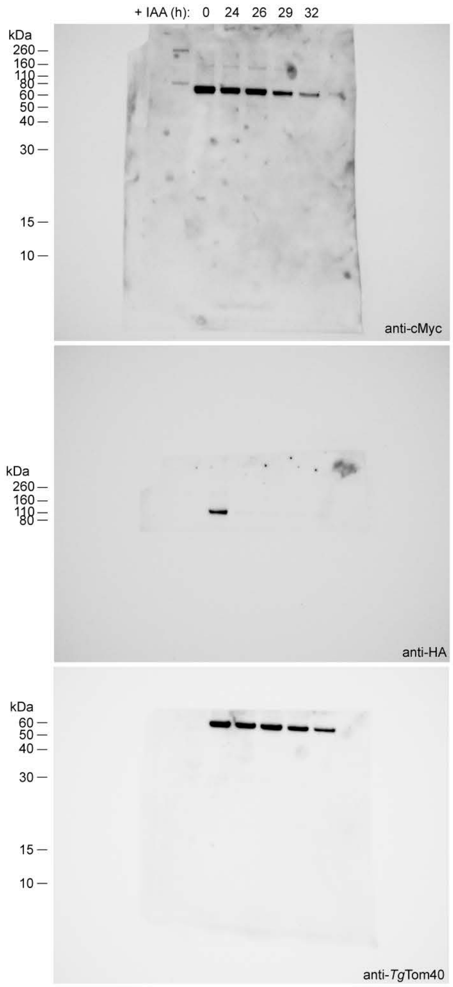

Repeat 4

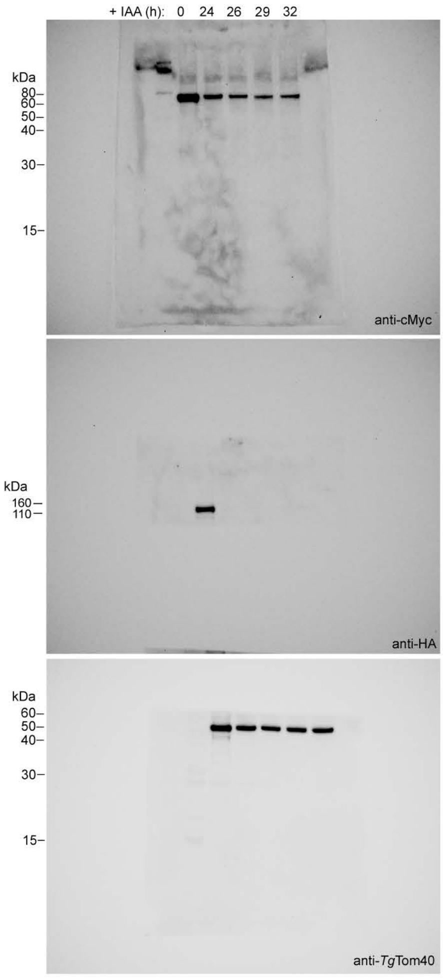

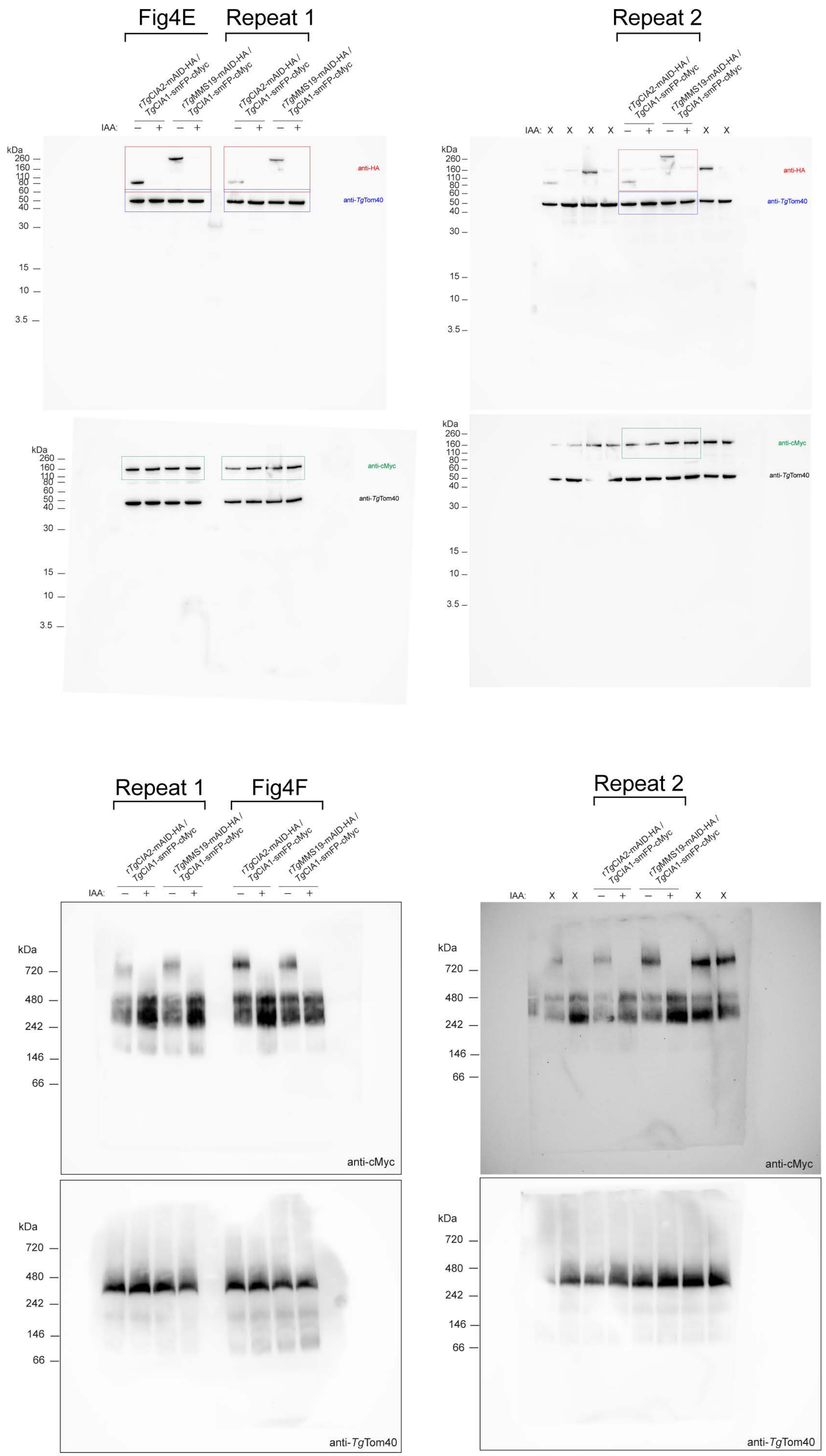

Fig4E
Repeat 1
Repeat 2
rTgCIA2-mAID-HA / TgCIA1-smFP-cMyc
rTgMMS19-mAID-HA / TgCIA1-smFP-cMyc
IAA:
kDa
260
160
110
80
60
50
40
30
15
10
3.5
anti-HA
anti-TgTom40
anti-cMyc
Repeat 1
Fig4F
Repeat 2
720
480
242
146
66
anti-cMyc
anti-TgTom40

Fig4A

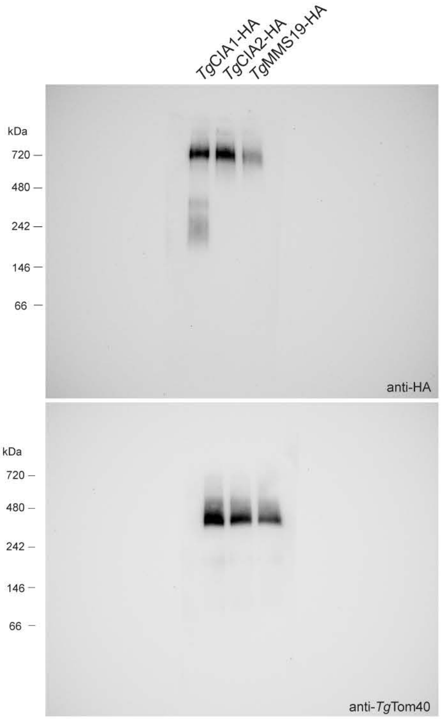

Repeat 1 Fig4B

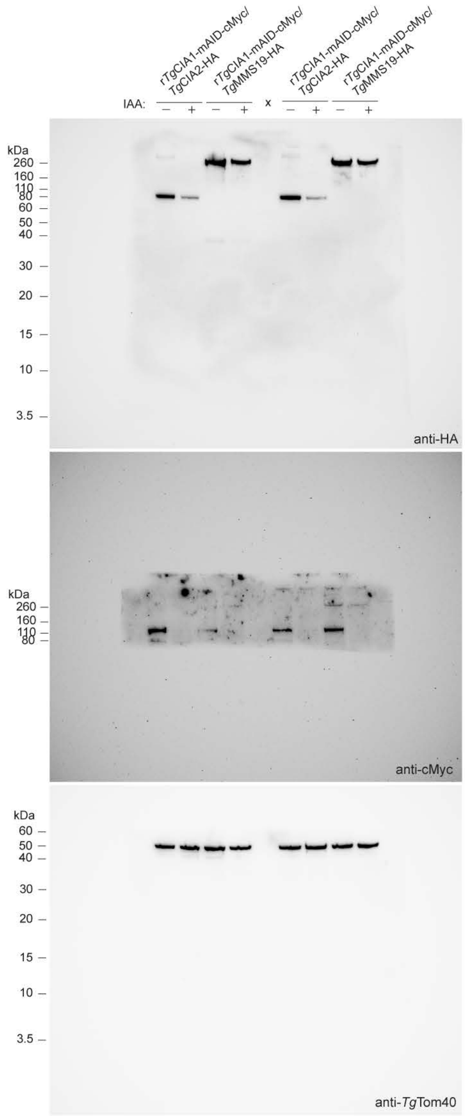

Repeat 2

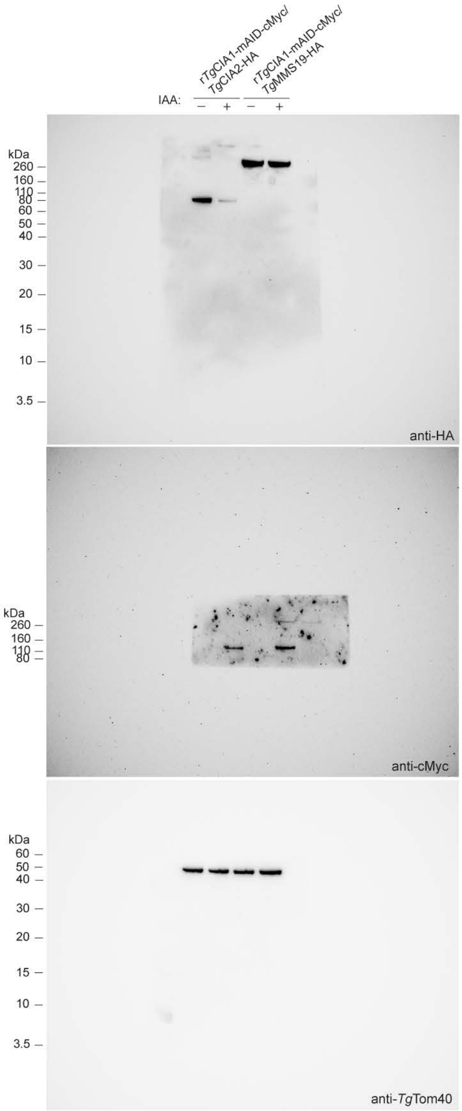

Fig4D

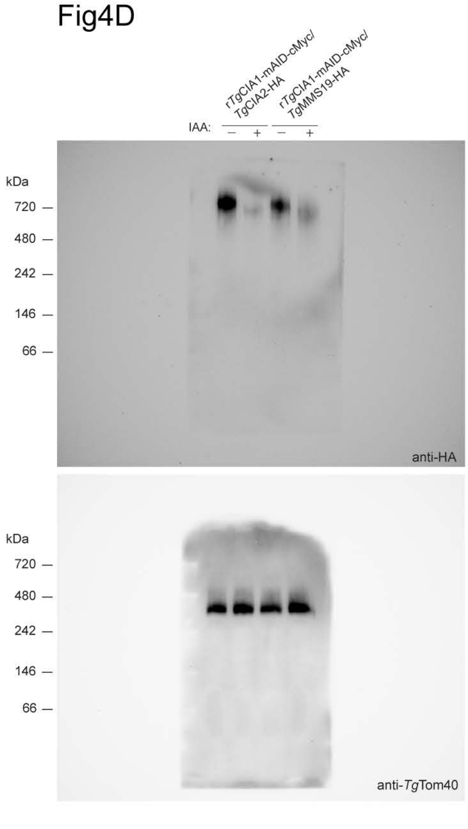

Repeat 1

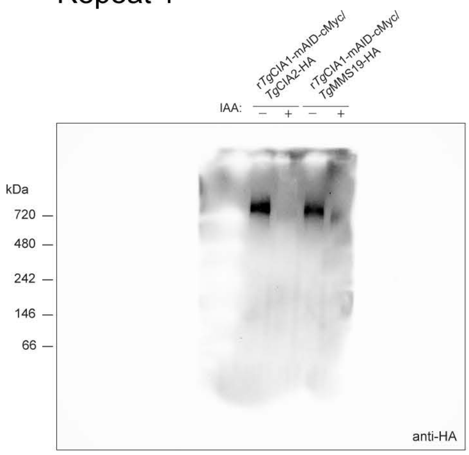

Repeat 2

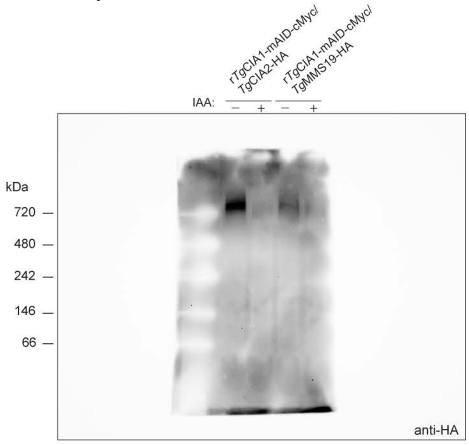

Fig6B

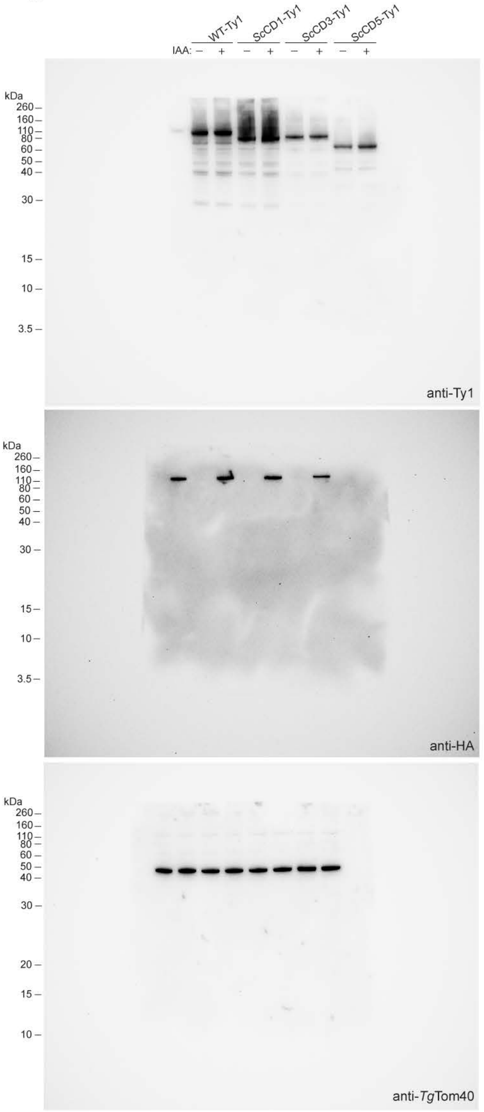

Repeat 1

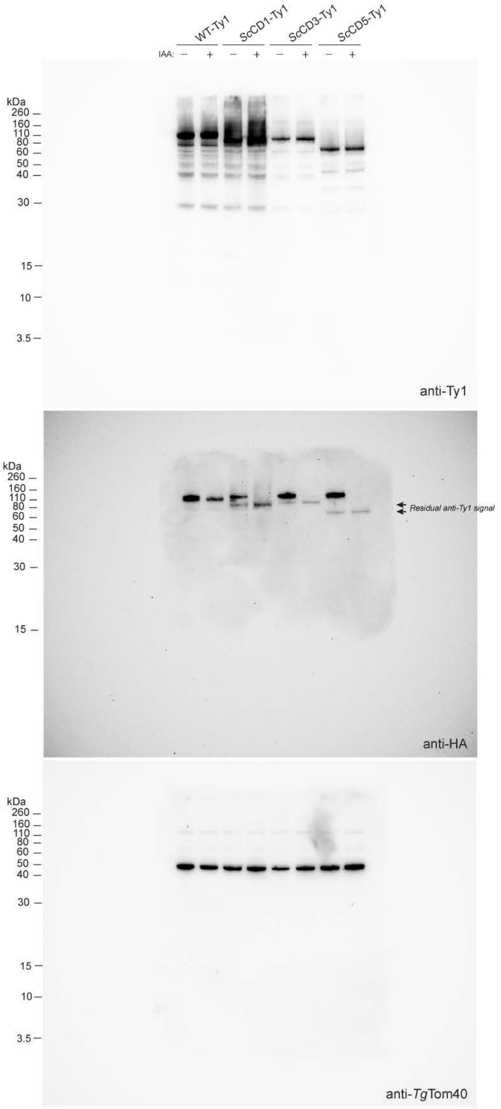

Repeat 2

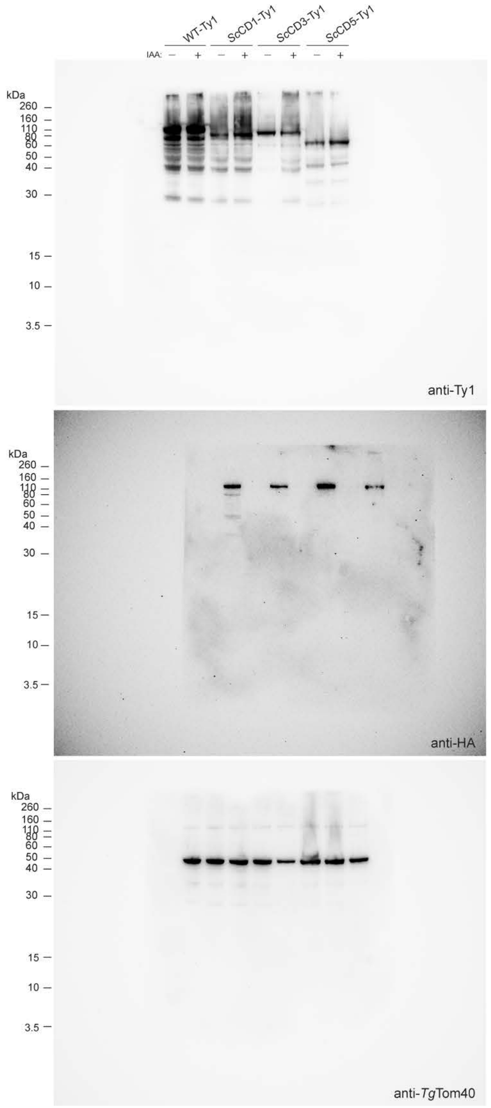

Fig6E

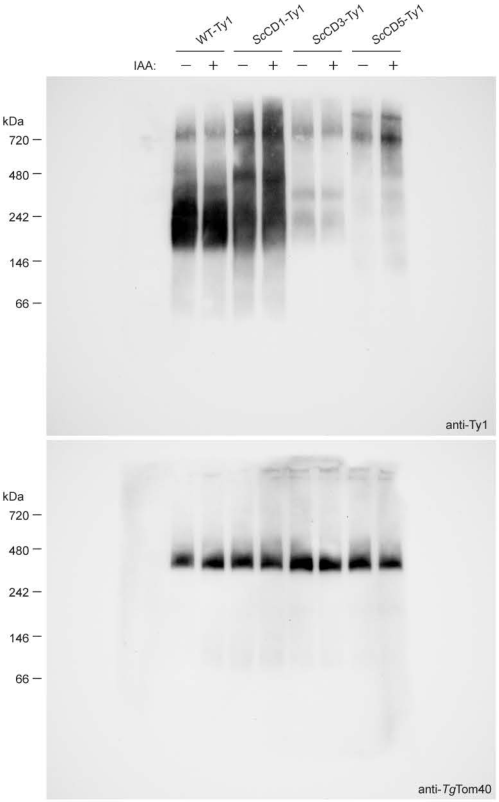

Repeat 1

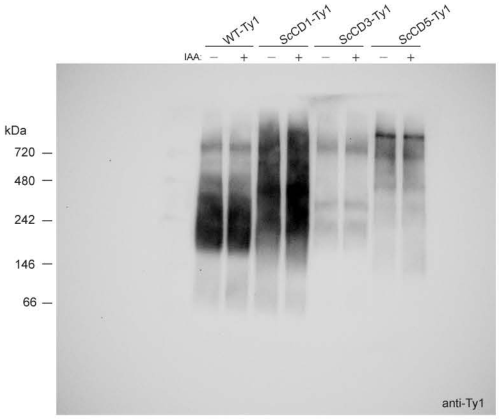

Repeat 2

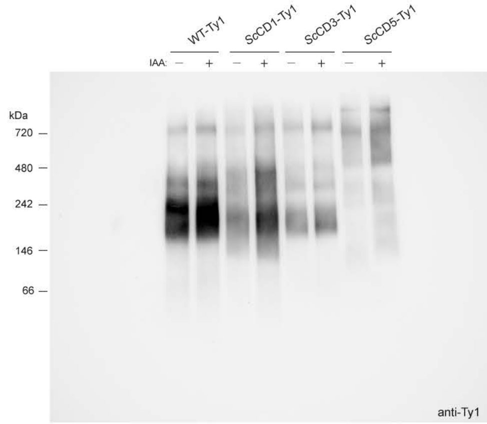

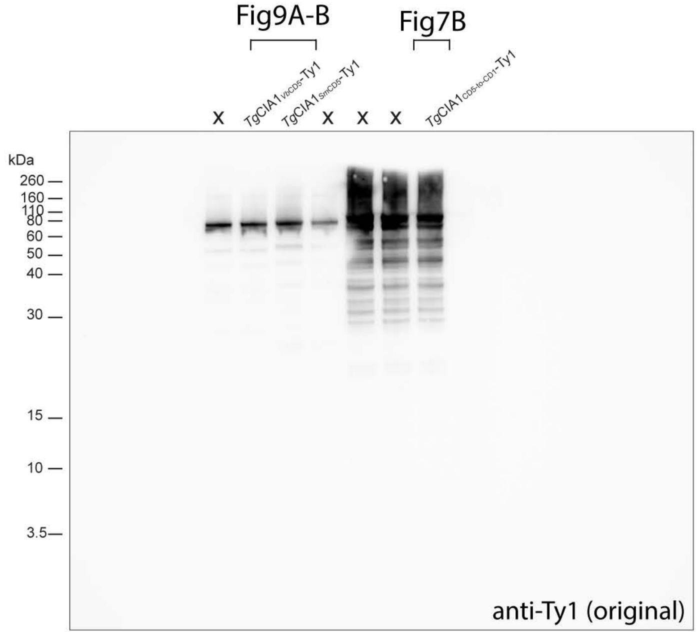
Fig9A-B
Fig7B
TgCIA1VbCD5-Ty1
TgCIA1SmCD5-Ty1
TgCIA1CD5-to-CD1-Ty1
X
X
X
X
kDa
260
160
110
80
60
50
40
30
15
10
3.5
anti-Ty1 (original)
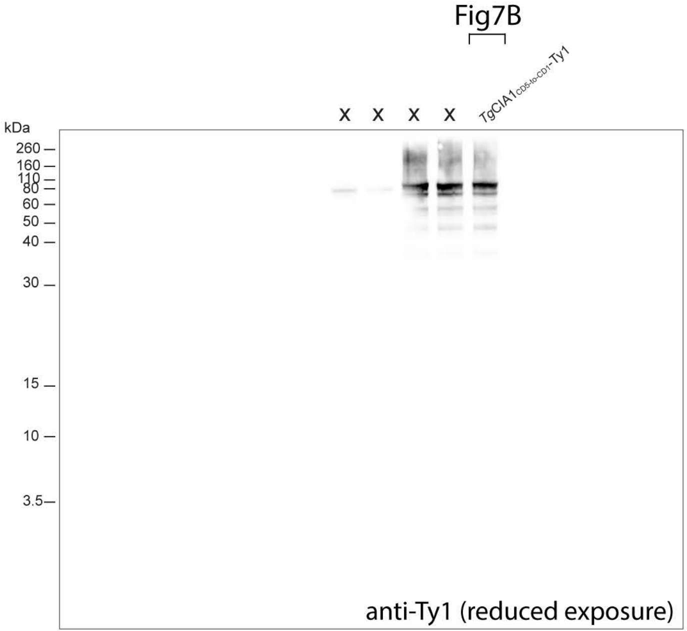
Fig7B
TgCIA1CD5-to-CD1-Ty1
X
X
X
X
kDa
260
160
110
80
60
50
40
30
15
10
3.5
anti-Ty1 (reduced exposure)

Fig8F

r*Tg*NBP35-cmyc/
*Tg*CIA1$_{WT}$-HA

r*Tg*NBP35-cmyc/
*Tg*CIA1$_{WT}$-HA
+ATc control

r*Tg*NBP35-cmyc/
*Tg*CIA1$_{Y527A}$-HA

r*Tg*NBP35-cmyc/
*Tg*CIA1$_{W526A}$-HA

r*Tg*NBP35-cmyc/
*Tg*CIA1$_{R533A}$-HA

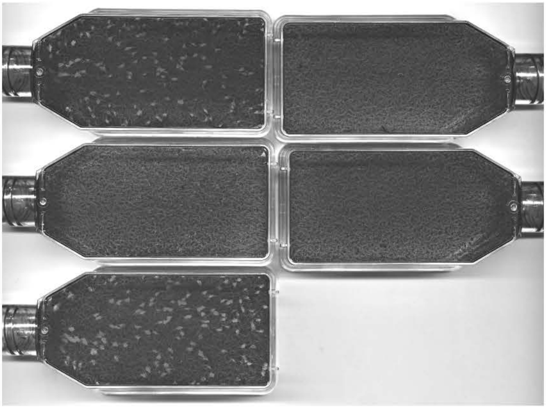

Repeat 1

r*Tg*NBP35-cmyc/
*Tg*CIA1$_{WT}$-HA

r*Tg*NBP35-cmyc/
*Tg*CIA1$_{WT}$-HA
+ATc control

r*Tg*NBP35-cmyc/
*Tg*CIA1$_{W526A}$-HA

r*Tg*NBP35-cmyc/
*Tg*CIA1$_{Y527A}$-HA

r*Tg*NBP35-cmyc/
*Tg*CIA1$_{R533A}$-HA

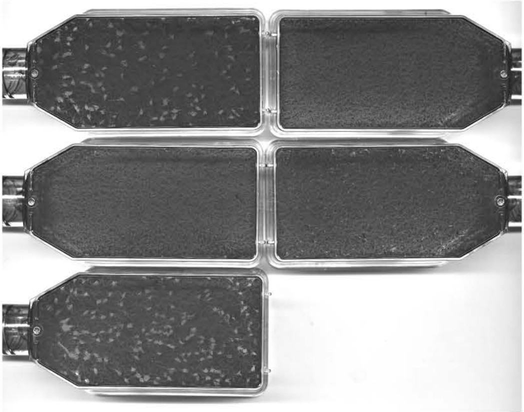

Repeat 2

r*Tg*NBP35-cmyc/
*Tg*CIA1$_{WT}$-HA

r*Tg*NBP35-cmyc/
*Tg*CIA1$_{WT}$-HA
+ATc control

r*Tg*NBP35-cmyc/
*Tg*CIA1$_{W526A}$-HA

r*Tg*NBP35-cmyc/
*Tg*CIA1$_{Y527A}$-HA

r*Tg*NBP35-cmyc/
*Tg*CIA1$_{W533A}$-HA

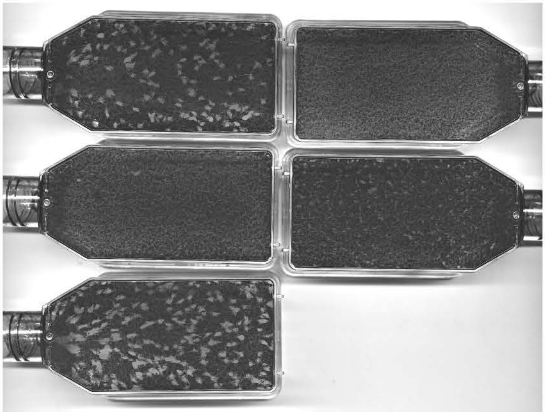

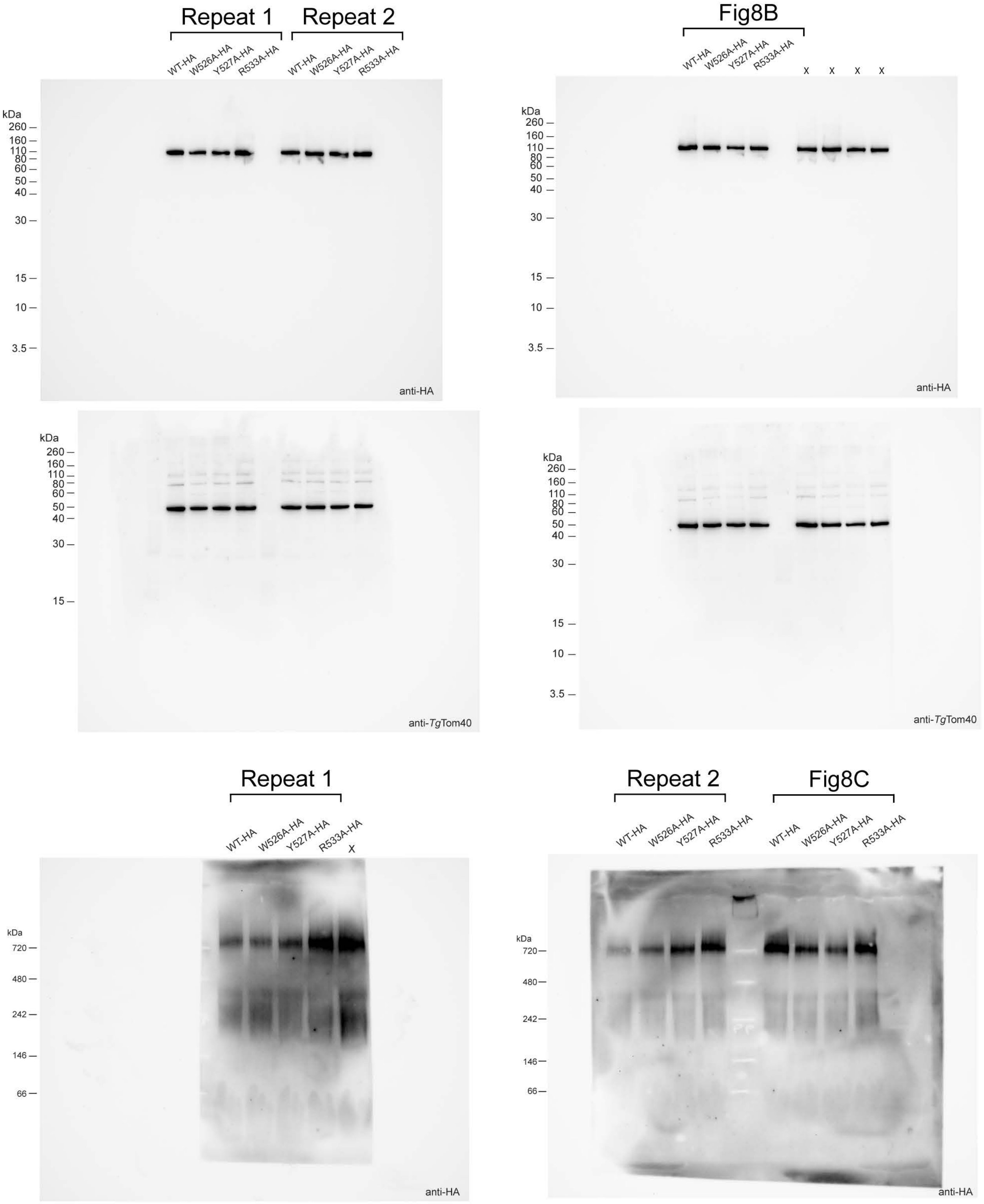

Repeat 1
Repeat 2
WT-HA
W526A-HA
Y527A-HA
R533A-HA
kDa
260
160
110
80
60
50
40
30
15
10
3.5
anti-HA
Fig8B
x
anti-*Tg*Tom40
720
480
242
146
66
X
Fig8C

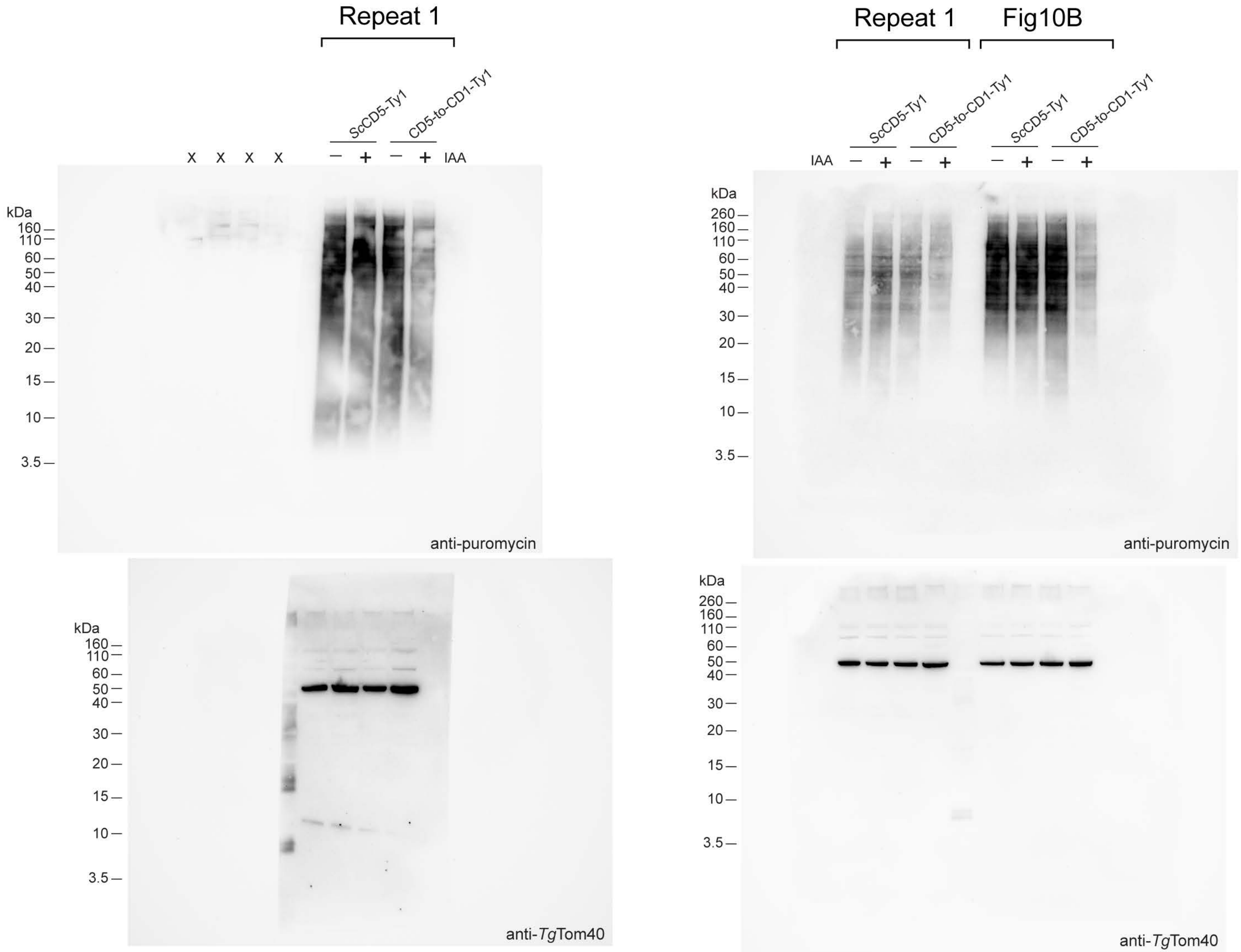

Repeat 1
ScCD5-Ty1
CD5-to-CD1-Ty1
X X X X
− + − + IAA
kDa
160
110
60
50
40
30
20
15
10
3.5
anti-puromycin
kDa
160
110
60
50
40
30
20
15
10
3.5
anti-TgTom40
Repeat 1
Fig10B
ScCD5-Ty1
CD5-to-CD1-Ty1
ScCD5-Ty1
CD5-to-CD1-Ty1
IAA − + − + − + − +
kDa
260
160
110
60
50
40
30
20
15
10
3.5
anti-puromycin
kDa
260
160
110
60
50
40
30
20
15
10
3.5
anti-TgTom40

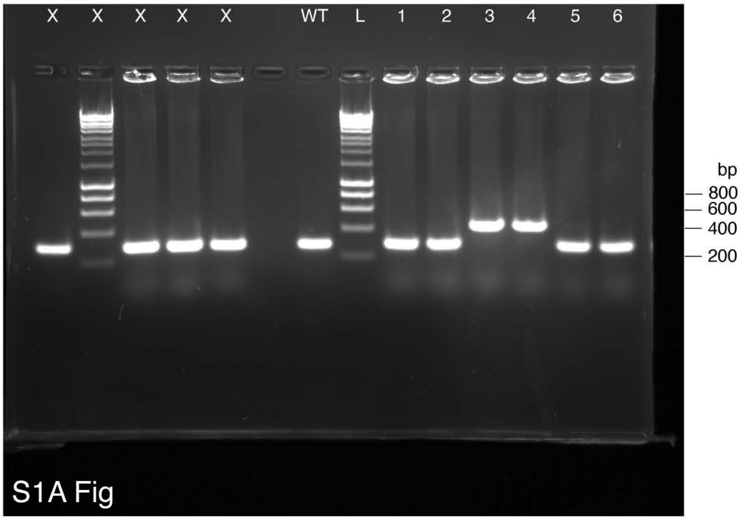
X X X X X WT L 1 2 3 4 5 6
bp
800
600
400
200
S1A Fig

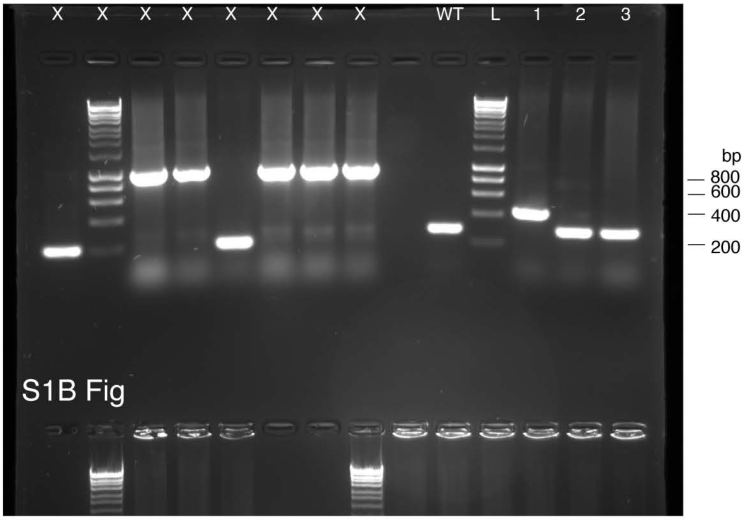
X X X X X X X X WT L 1 2 3
bp
800
600
400
200
S1B Fig

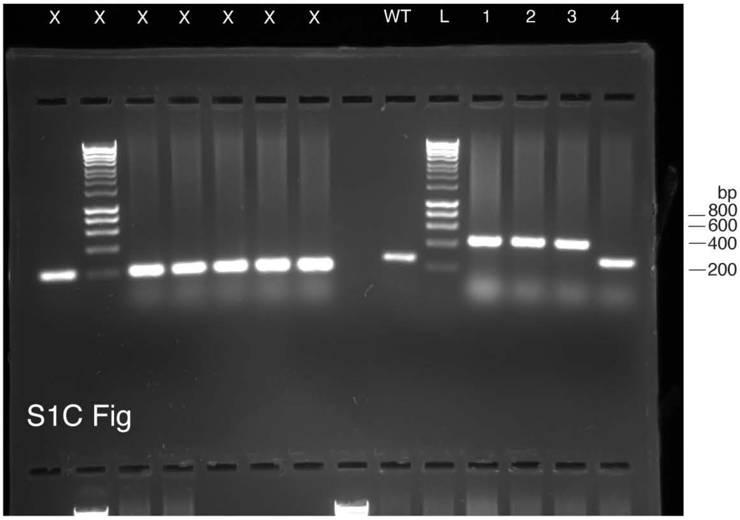
X X X X X X X WT L 1 2 3 4
bp
800
600
400
200
S1C Fig

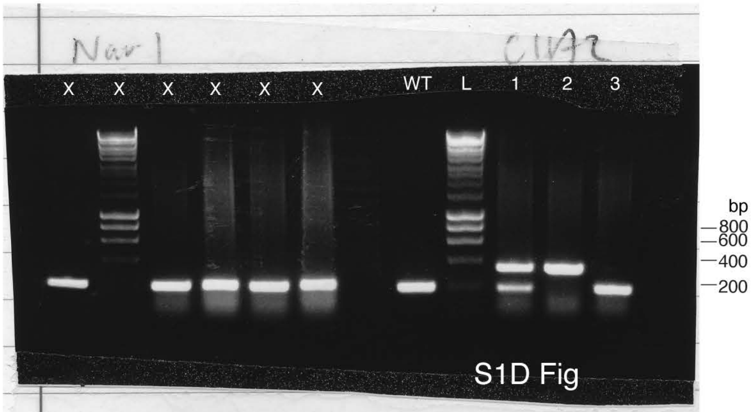
X X X X X X WT L 1 2 3
bp
800
600
400
200
S1D Fig

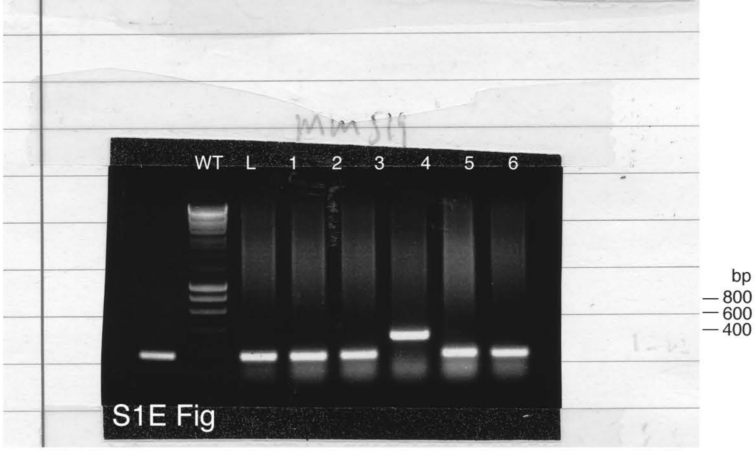
WT L 1 2 3 4 5 6
bp
800
600
400
S1E Fig

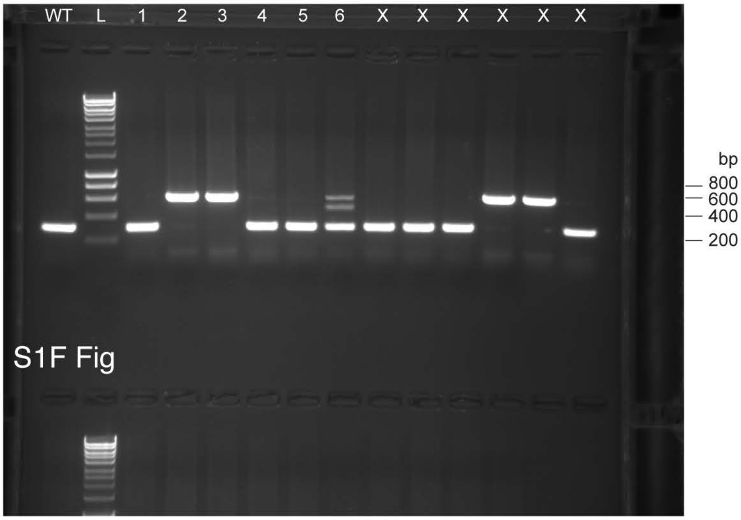
WT L 1 2 3 4 5 6 X X X X X X
bp
800
600
400
200
S1F Fig

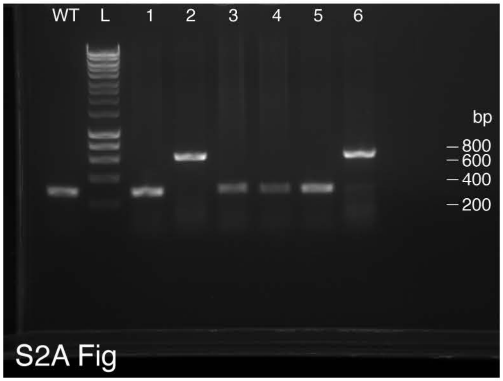

S2A Fig

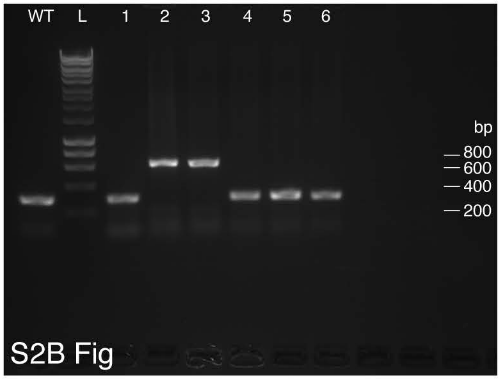

S2B Fig

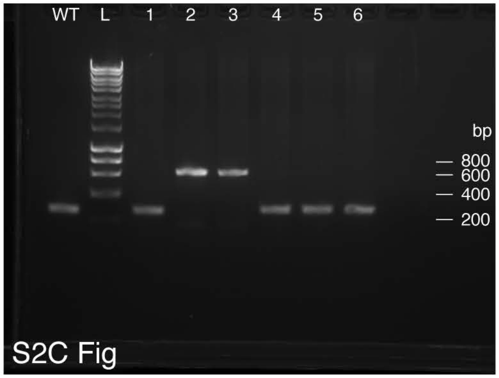

S2C Fig

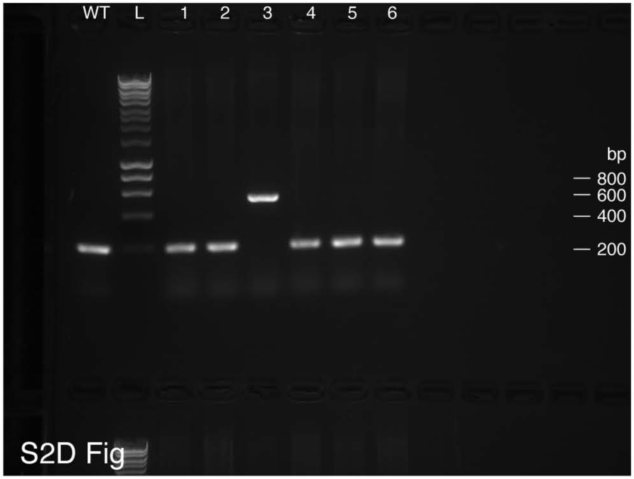

S2D Fig

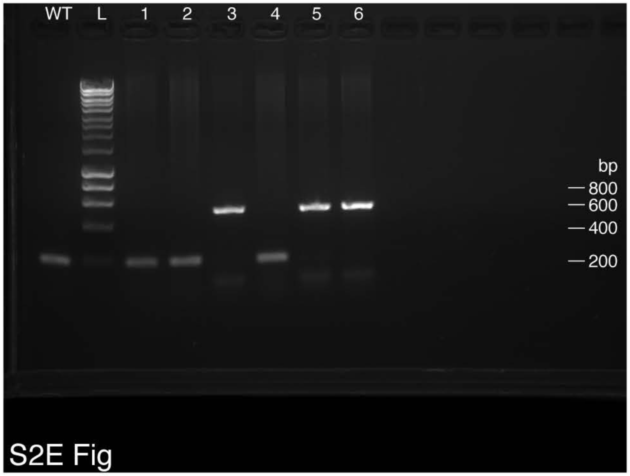

S2E Fig

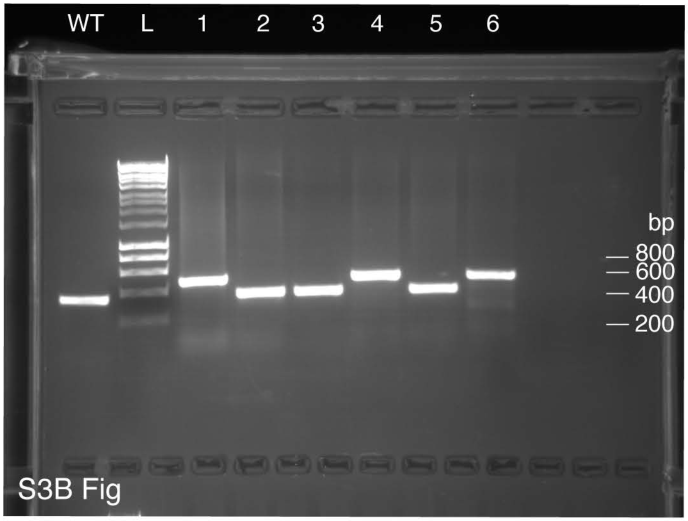

S3B Fig

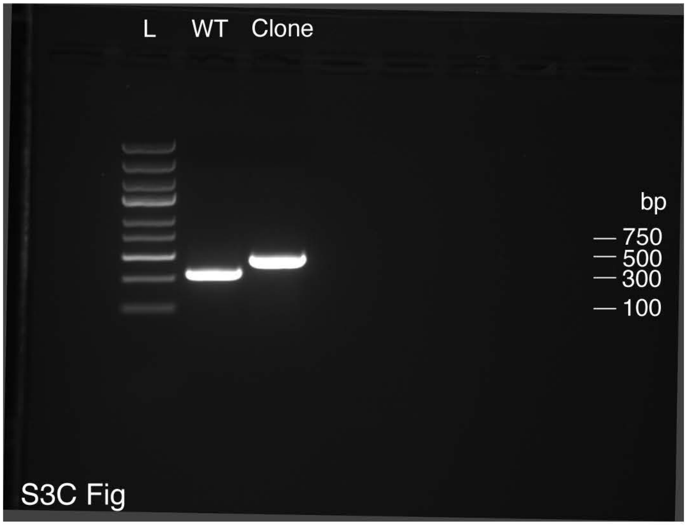

S3C Fig

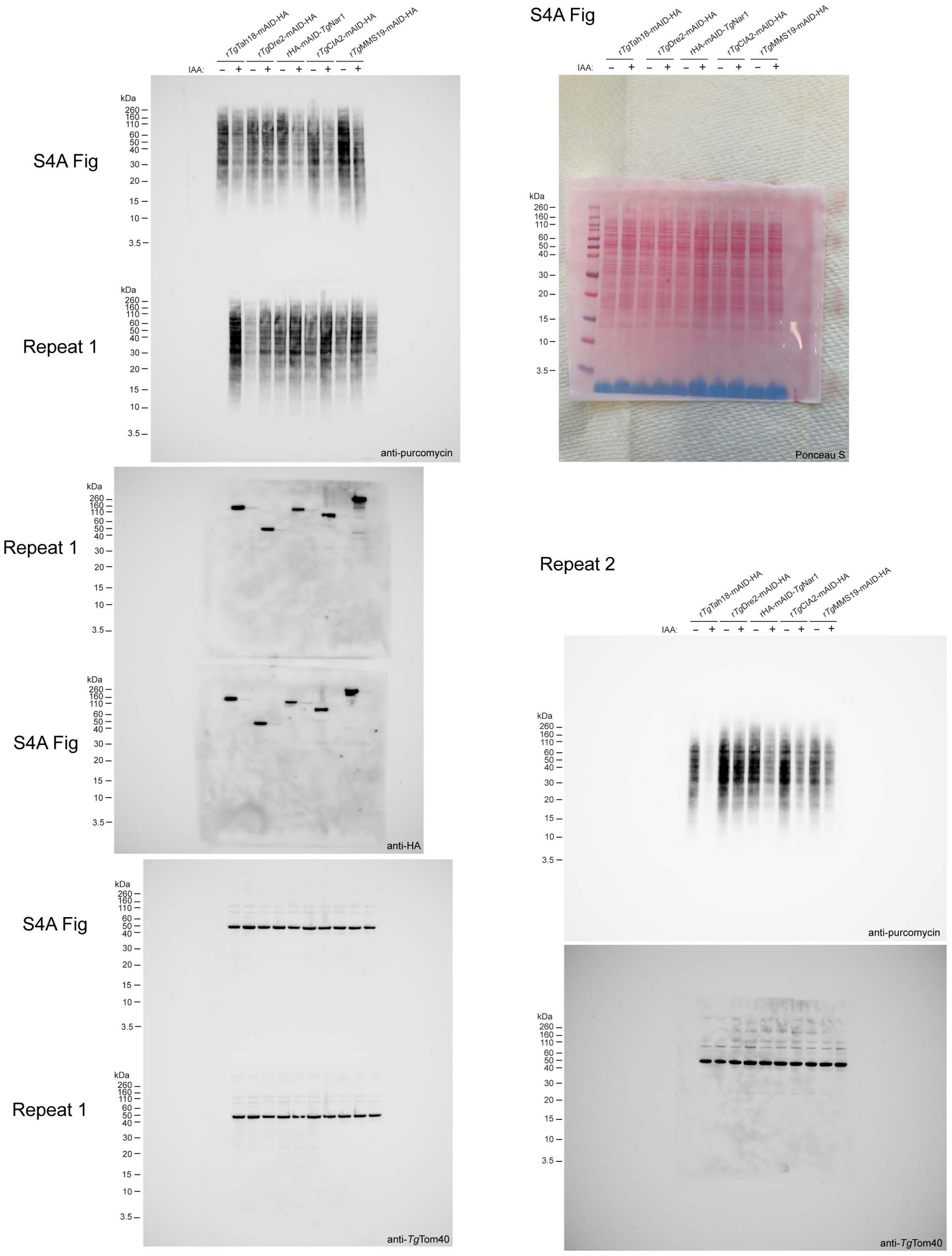
S4A Fig
Repeat 1
Repeat 2
rTgTah18-mAID-HA
rTgDre2-mAID-HA
rHA-mAID-TgNar1
rTgCIA2-mAID-HA
rTgMMS19-mAID-HA
IAA:
kDa
260
160
110
60
50
40
30
20
15
10
3.5
anti-purcomycin
Ponceau S
anti-HA
anti-TgTom40

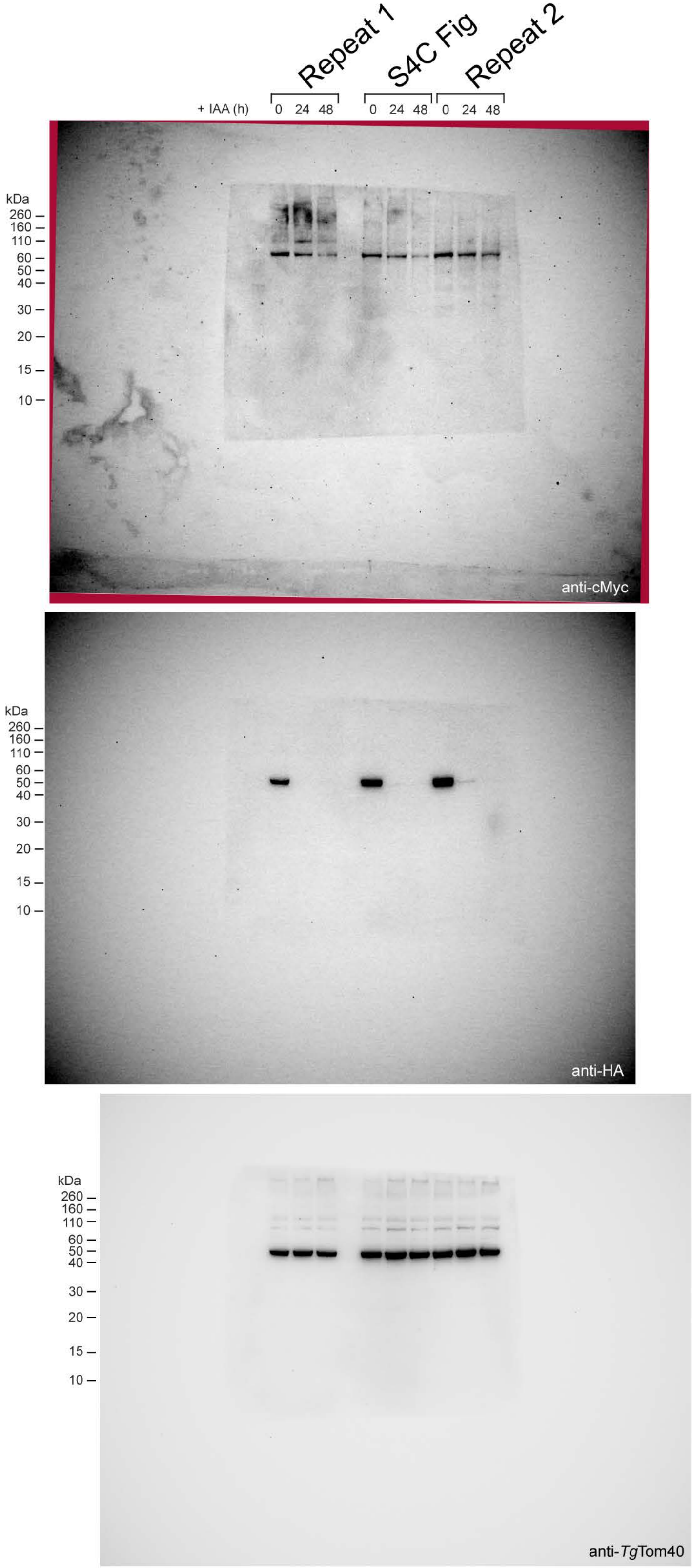

Repeat 1
S4C Fig
Repeat 2
+ IAA (h)
0
24
48
0
24
48
0
24
48
kDa
260
160
110
60
50
40
30
20
15
10
anti-cMyc
kDa
260
160
110
60
50
40
30
20
15
10
anti-HA
kDa
260
160
110
60
50
40
30
20
15
10
anti-*Tg*Tom40

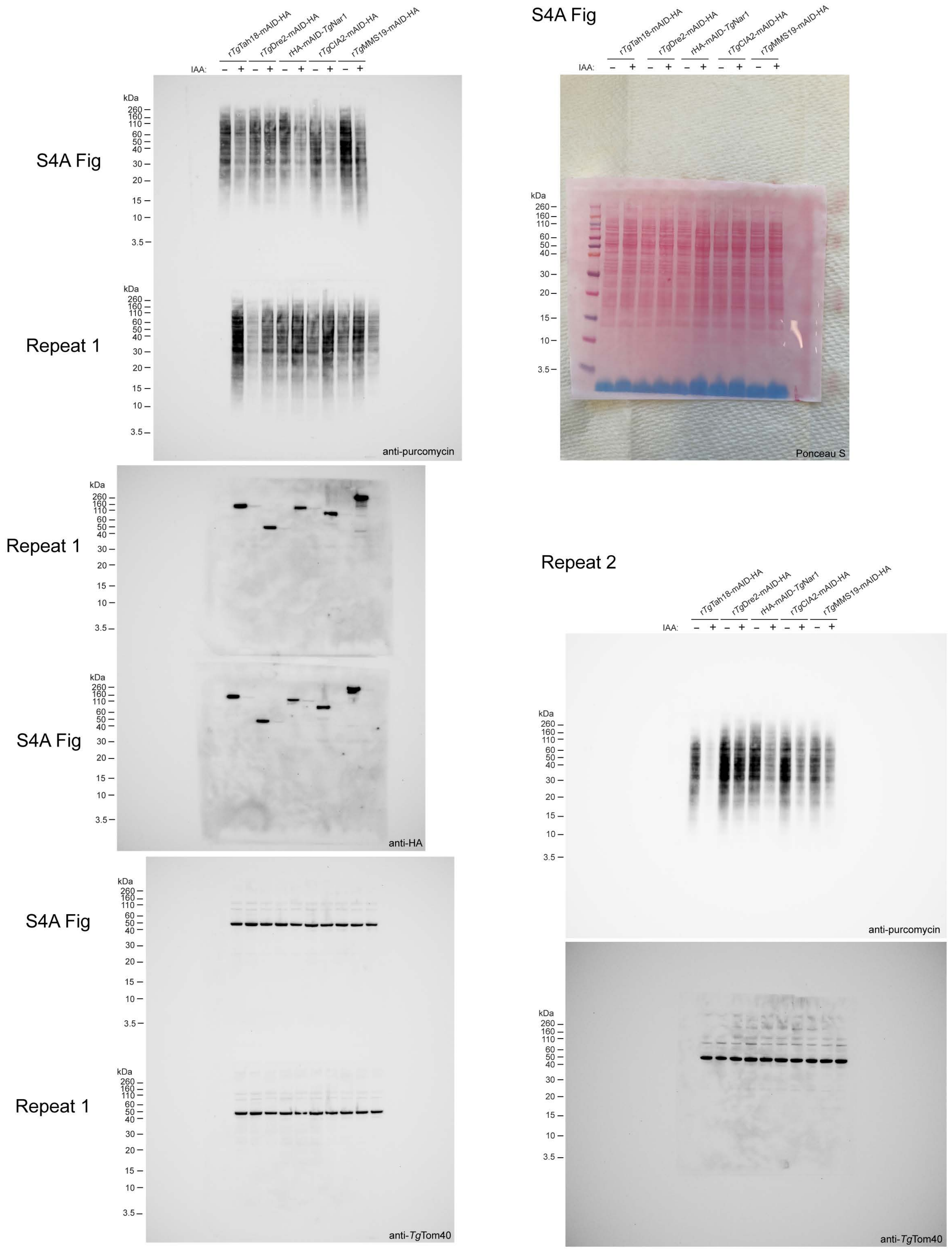
S4A Fig
Repeat 1
Repeat 2
rTgTah18-mAID-HA
rTgDre2-mAID-HA
rHA-mAID-TgNar1
rTgCIA2-mAID-HA
rTgMMS19-mAID-HA
IAA: – + – + – + – + – +
kDa
260
160
110
60
50
40
30
20
15
10
3.5
anti-purcomycin
Ponceau S
anti-HA
anti-TgTom40

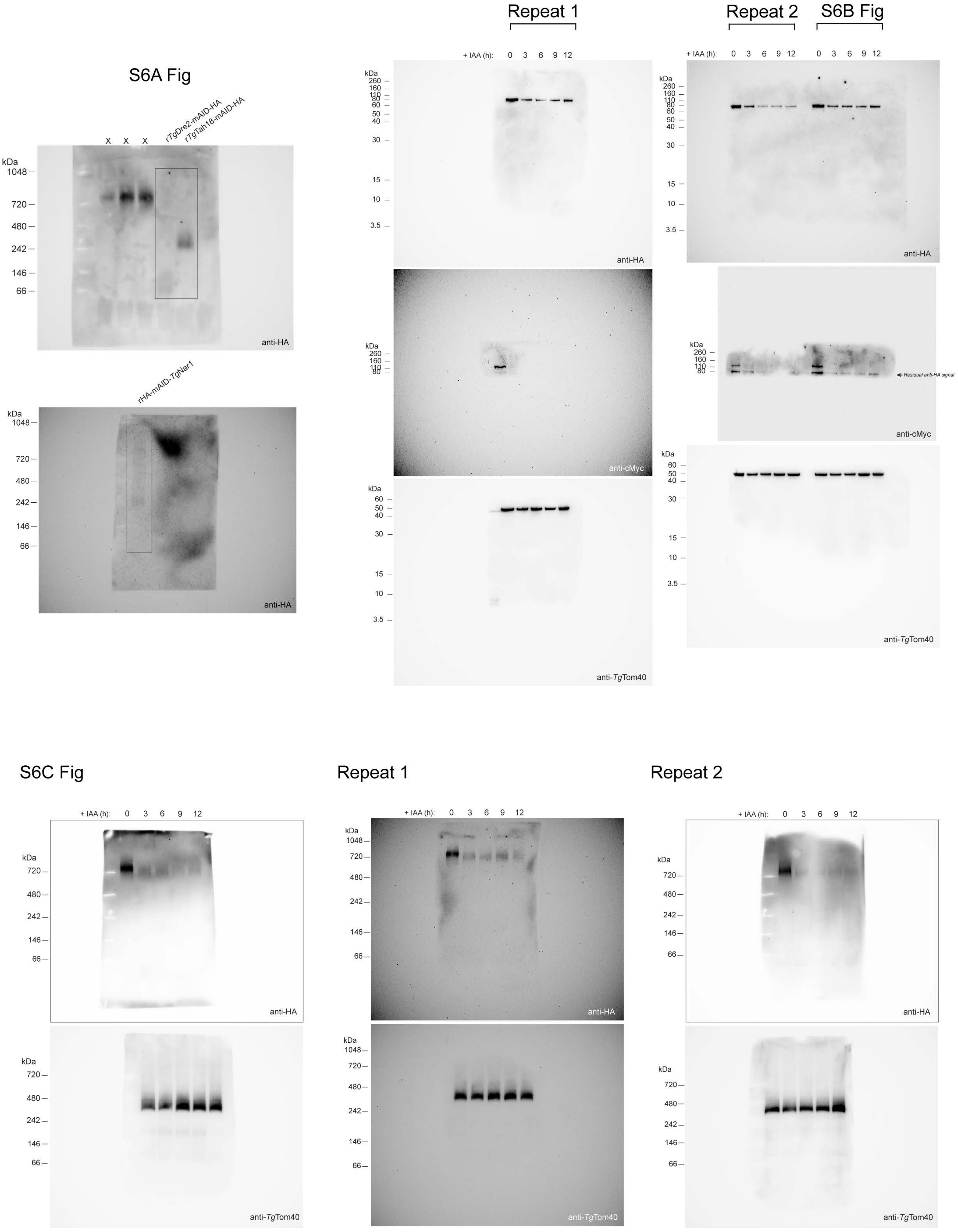
S6A Fig
Repeat 1
Repeat 2
S6B Fig
+ IAA (h): 0 3 6 9 12
kDa
rTgDre2-mAID-HA
rTgTah18-mAID-HA
rHA-mAID-TgNar1
anti-HA
anti-cMyc
anti-TgTom40
Residual anti-HA signal
S6C Fig
Repeat 1
Repeat 2

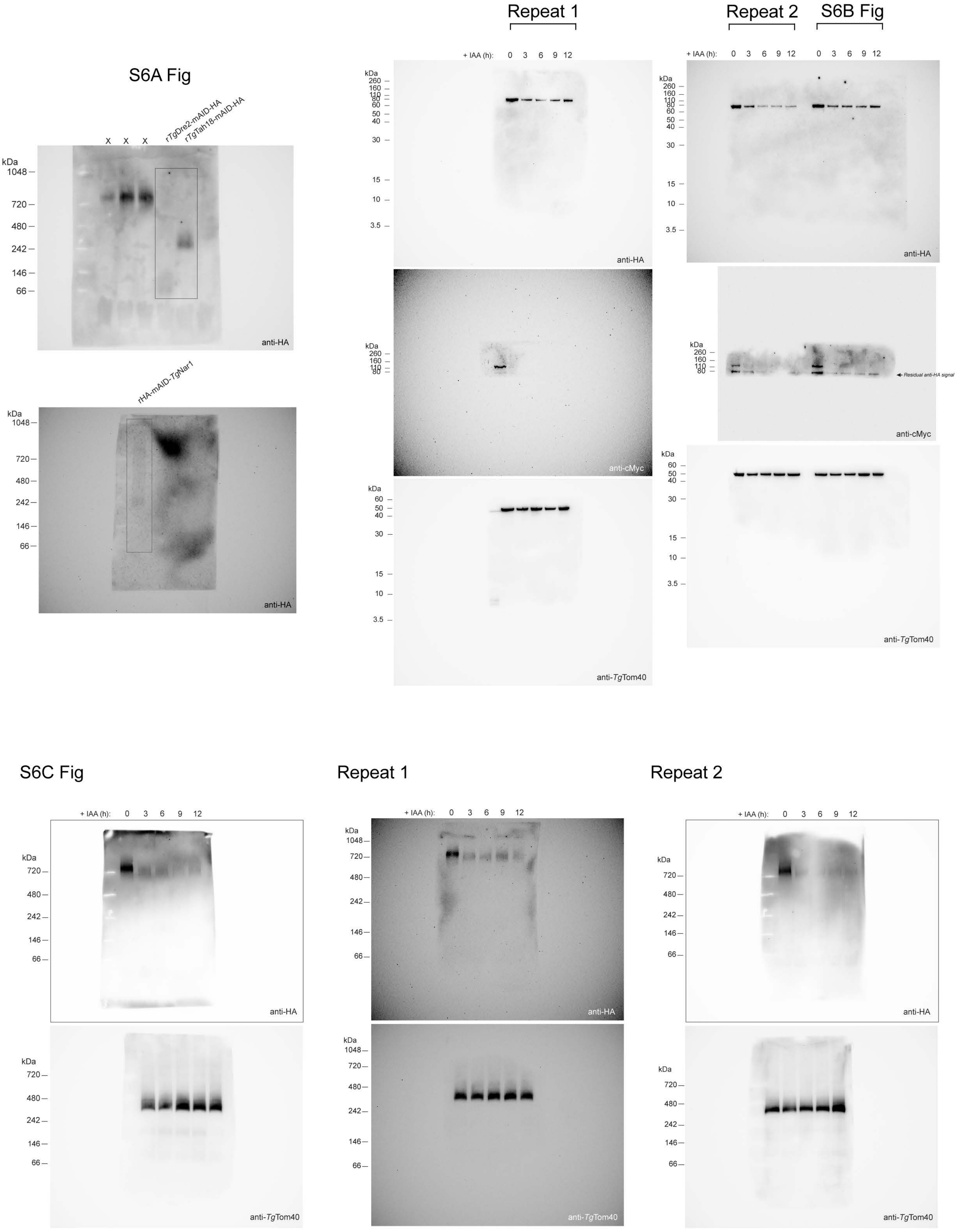
S6A Fig
X
X
X
rTgDre2-mAID-HA
rTgTah18-mAID-HA
kDa
1048
720
480
242
146
66
anti-HA
rHA-mAID-TgNar1
kDa
1048
720
480
242
146
66
anti-HA
Repeat 1
Repeat 2
S6B Fig
+ IAA (h):
0
3
6
9
12
kDa
260
160
110
80
60
50
40
30
15
10
3.5
anti-HA
anti-cMyc
Residual anti-HA signal
anti-TgTom40
S6C Fig
Repeat 1
Repeat 2
+ IAA (h):
0
3
6
9
12
kDa
1048
720
480
242
146
66
anti-HA
anti-TgTom40

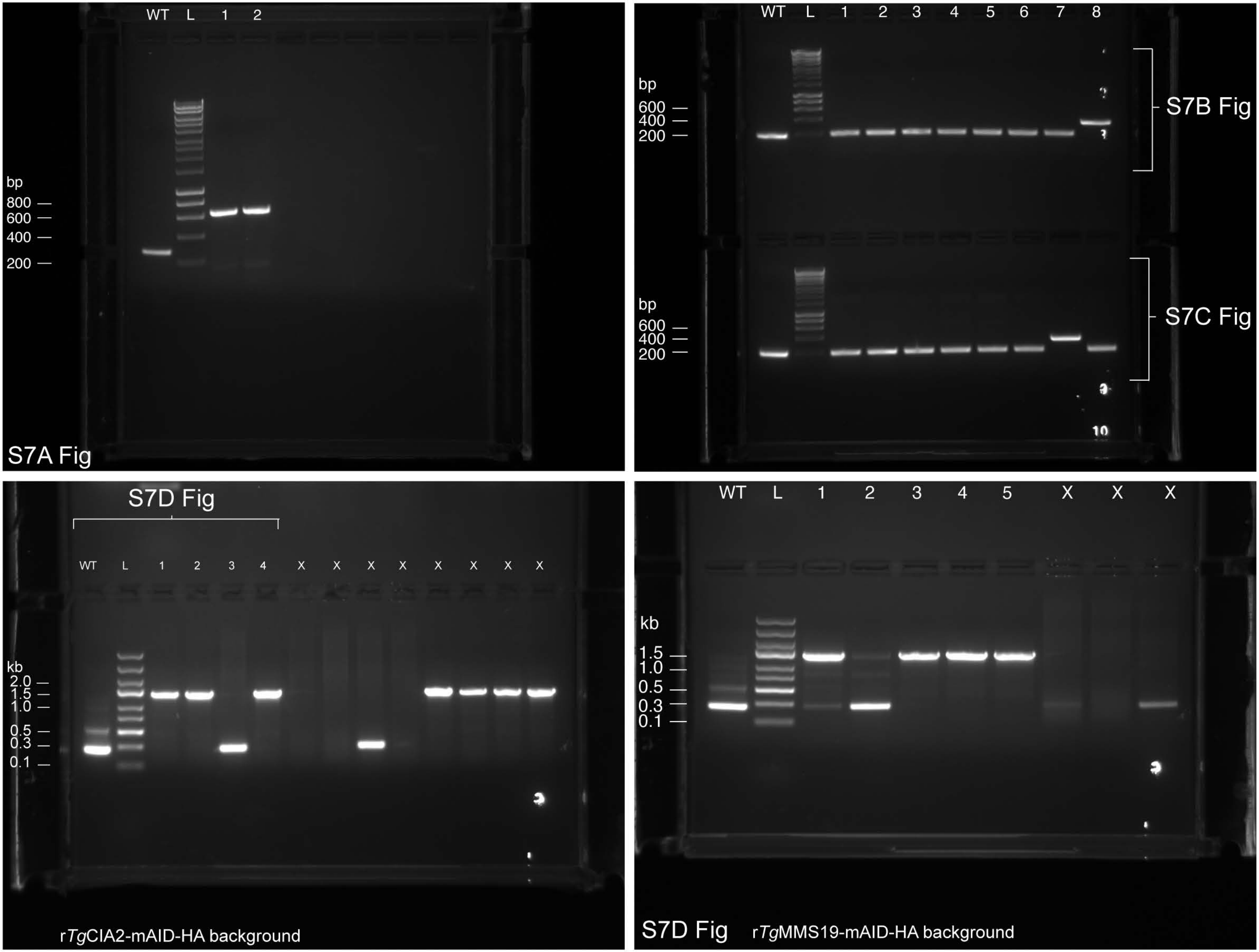

WT L 1 2
bp
800
600
400
200
S7A Fig
WT L 1 2 3 4 5 6 7 8
bp
600
400
200
S7B Fig
bp
600
400
200
S7C Fig
S7D Fig
WT L 1 2 3 4 X X X X X X X X
kb
2.0
1.5
1.0
0.5
0.3
0.1
rTgCIA2-mAID-HA background
WT L 1 2 3 4 5 X X X
kb
1.5
1.0
0.5
0.3
0.1
S7D Fig
rTgMMS19-mAID-HA background

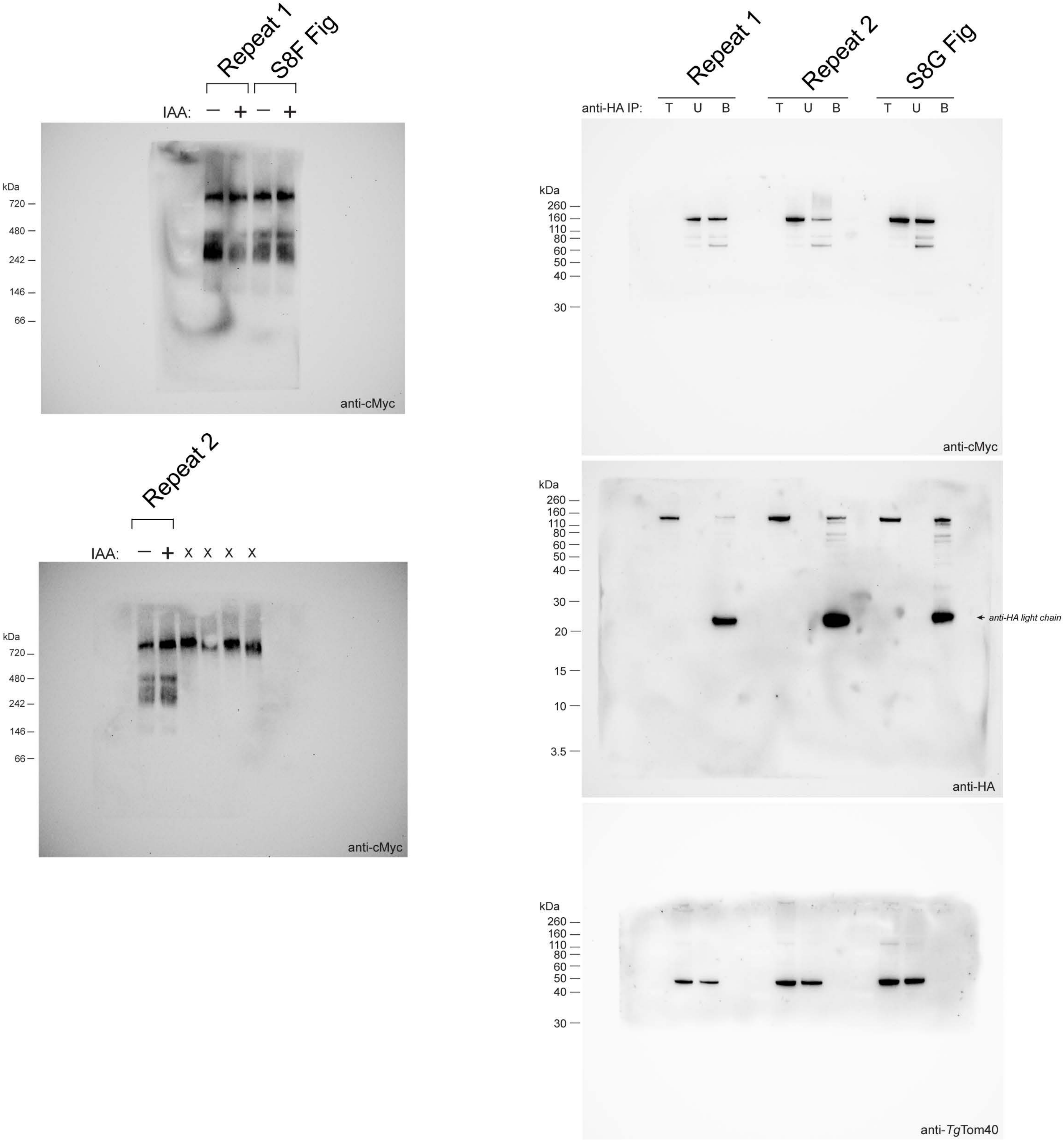
Repeat 1
S8F Fig
IAA: − + − +
kDa
720
480
242
146
66
anti-cMyc
Repeat 2
IAA: − + x x x x
kDa
720
480
242
146
66
anti-cMyc
Repeat 1
Repeat 2
S8G Fig
anti-HA IP: T U B T U B T U B
kDa
260
160
110
80
60
50
40
30
anti-cMyc
kDa
260
160
110
80
60
50
40
30
20
15
10
3.5
anti-HA light chain
anti-HA
kDa
260
160
110
80
60
50
40
30
anti-*Tg*Tom40

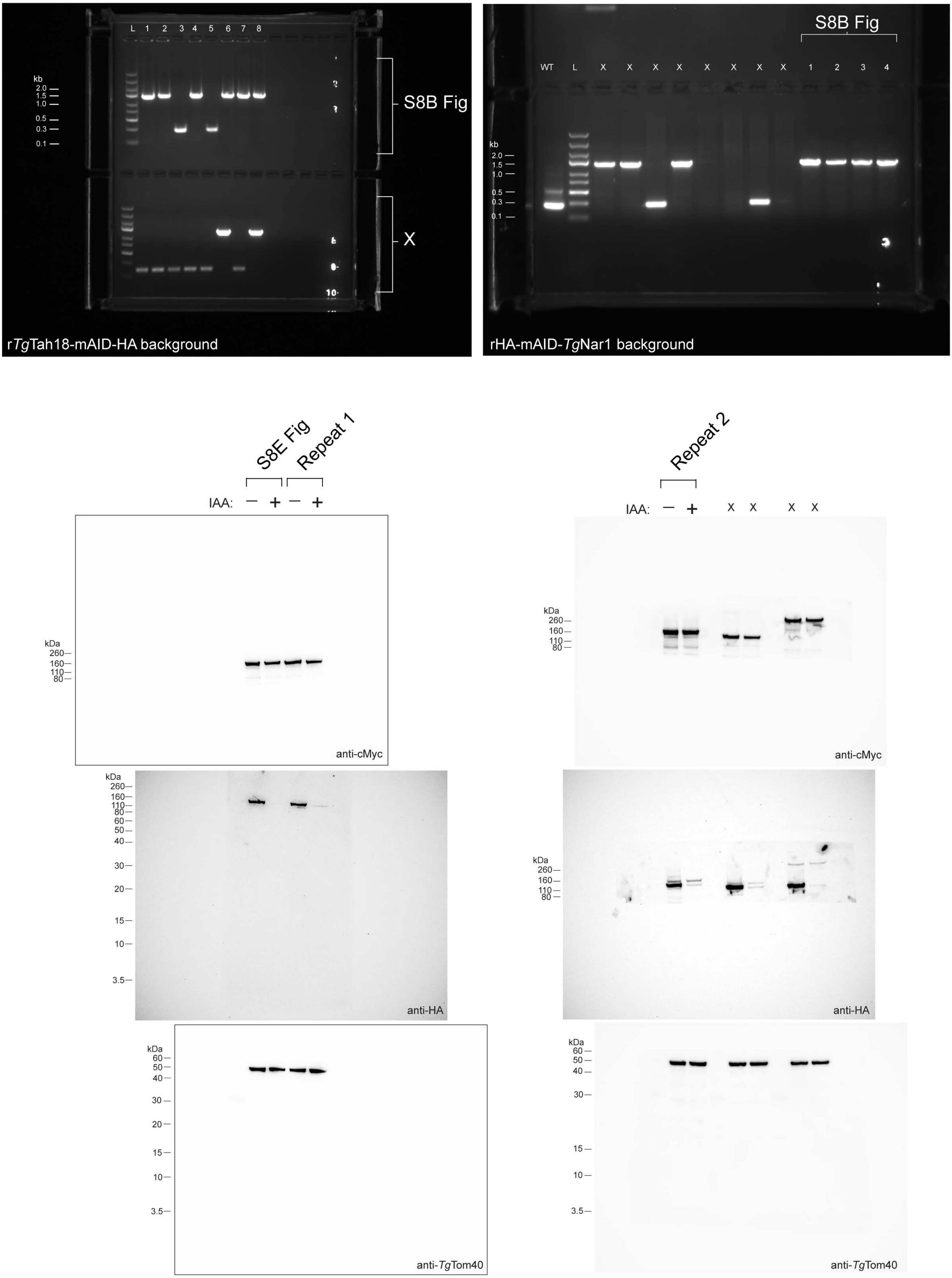

L 1 2 3 4 5 6 7 8
kb
2.0
1.5
1.0
0.5
0.3
0.1
S8B Fig
X
rTgTah18-mAID-HA background
S8B Fig
WT L X X X X X X X X 1 2 3 4
kb
2.0
1.5
1.0
0.5
0.3
0.1
rHA-mAID-TgNar1 background
S8E Fig
Repeat 1
IAA: − + − +
kDa
260
160
110
80
anti-cMyc
kDa
260
160
110
80
60
50
40
30
20
15
10
3.5
anti-HA
kDa
60
50
40
30
20
15
10
3.5
anti-TgTom40
Repeat 2
IAA: − + X X X X
kDa
260
160
110
80
anti-cMyc
kDa
260
160
110
80
anti-HA
kDa
60
50
40
30
15
10
3.5
anti-TgTom40

S10E Fig

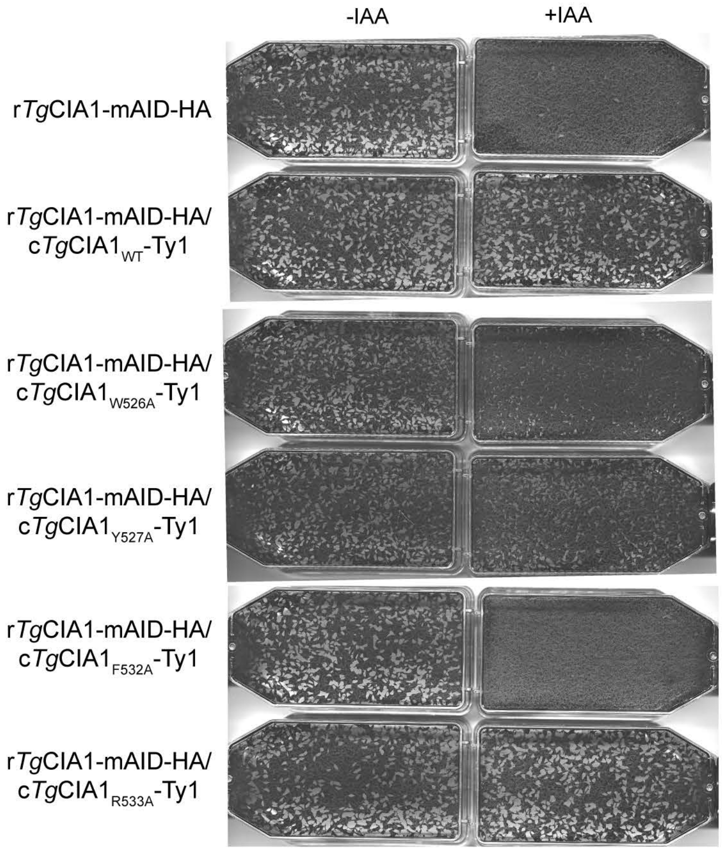

Repeat 1

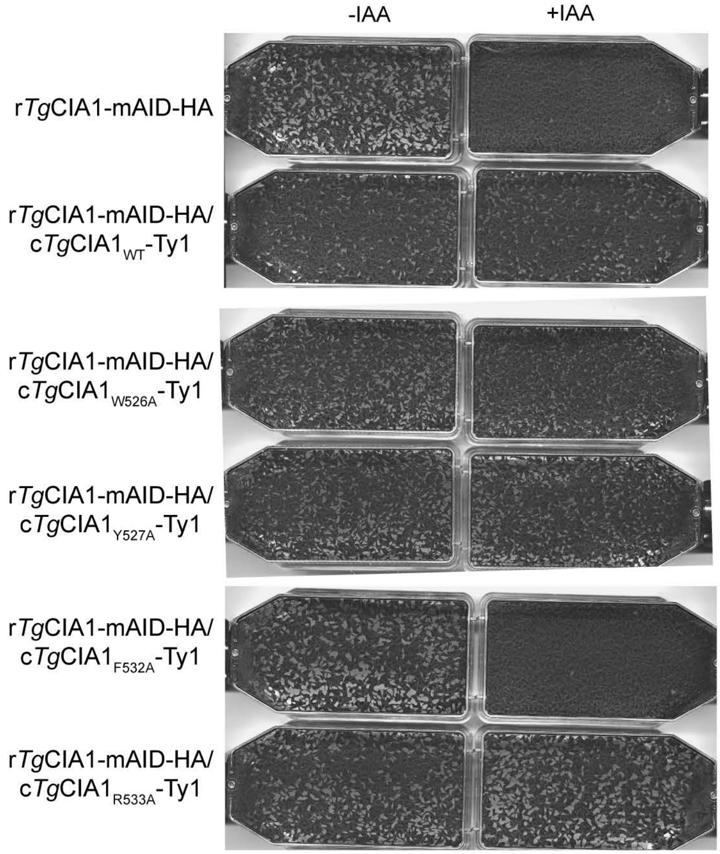

Repeat 2

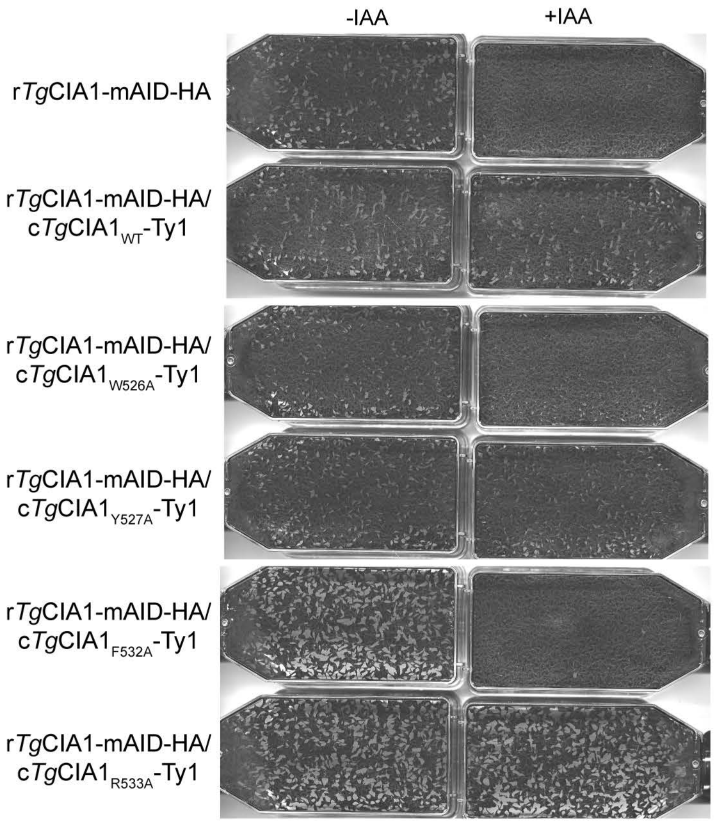

S10D Fig

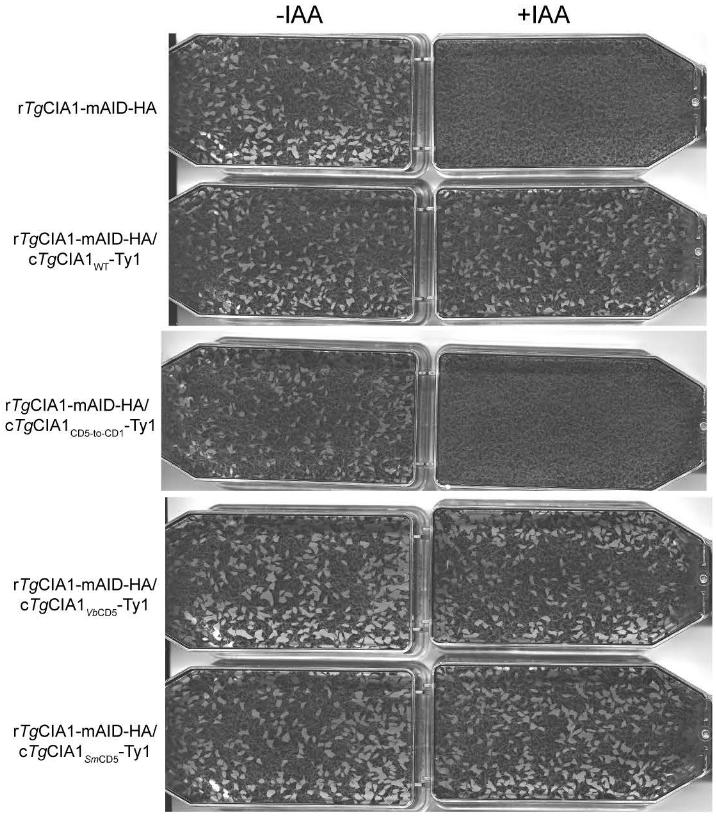

Repeat 1

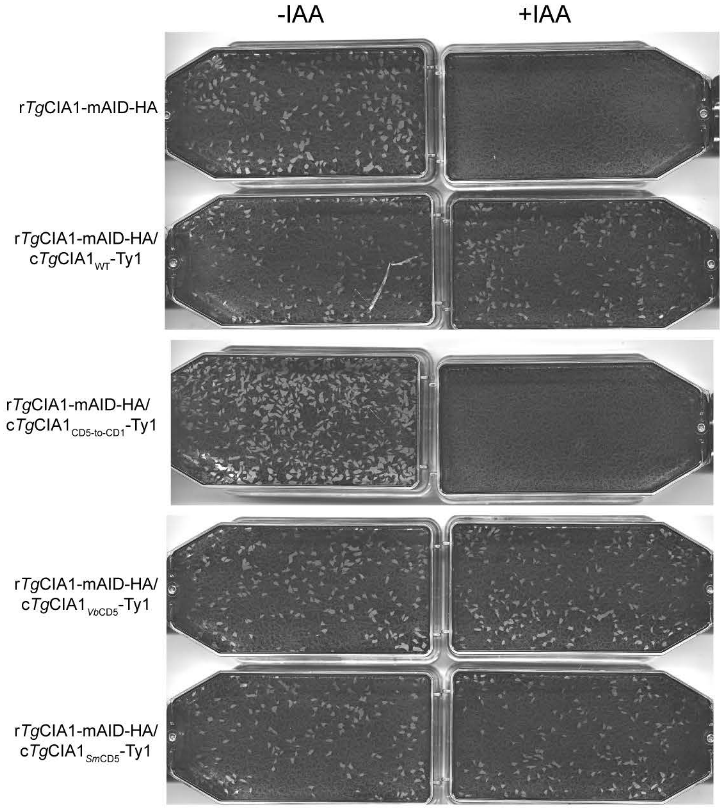

Repeat 2

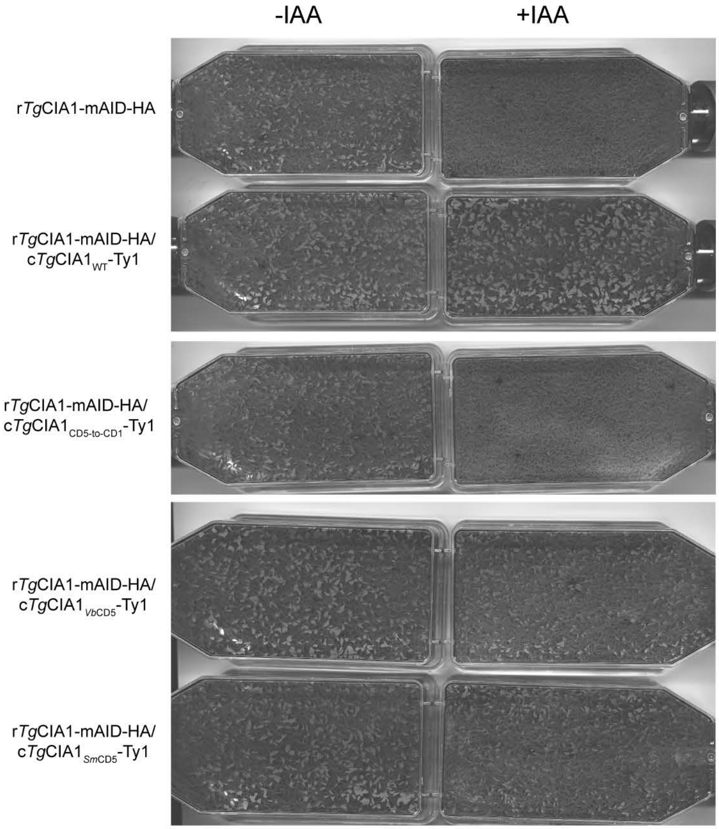

S10C Fig

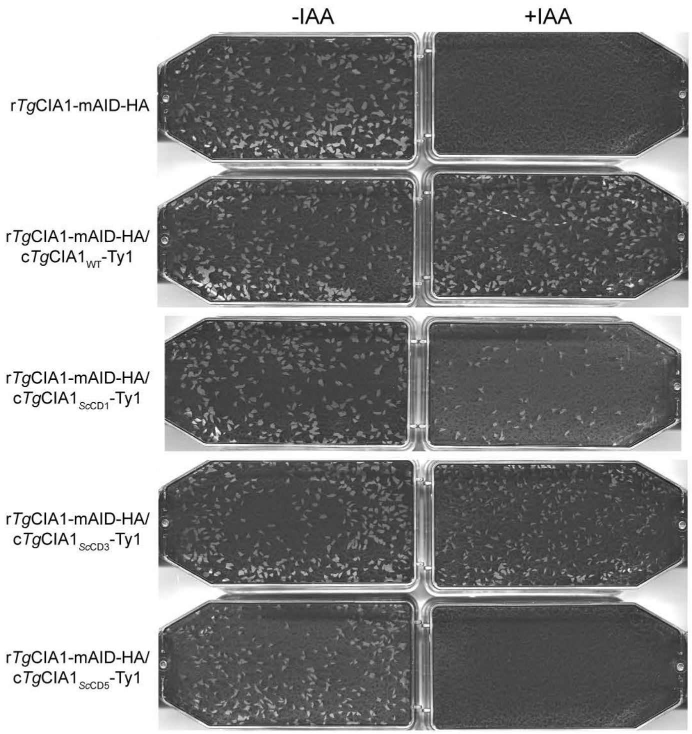

Repeat 1

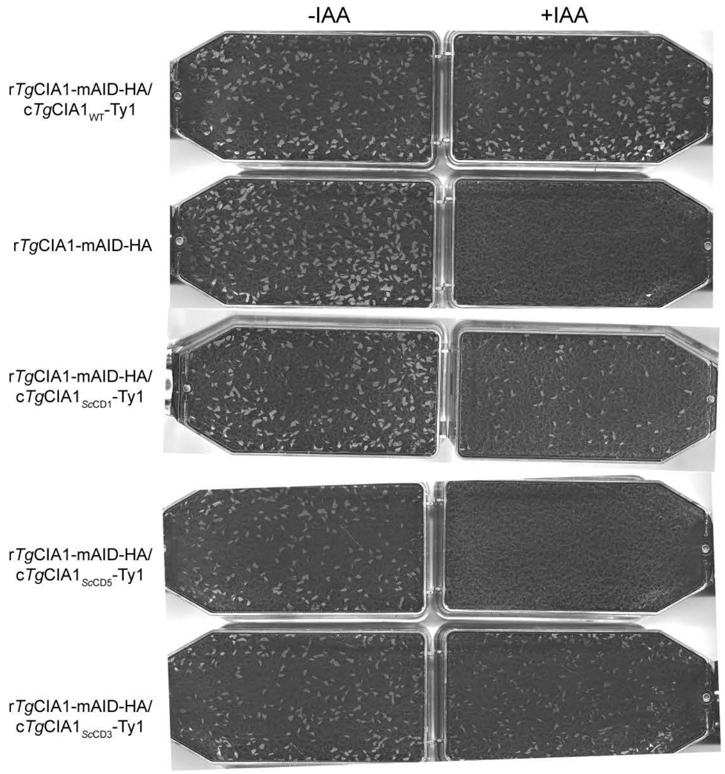

Repeat 2

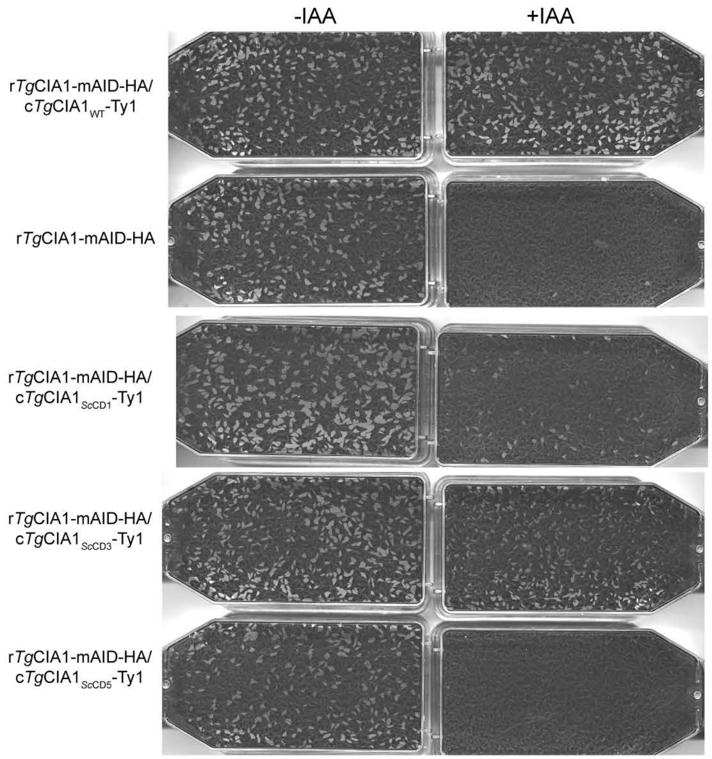

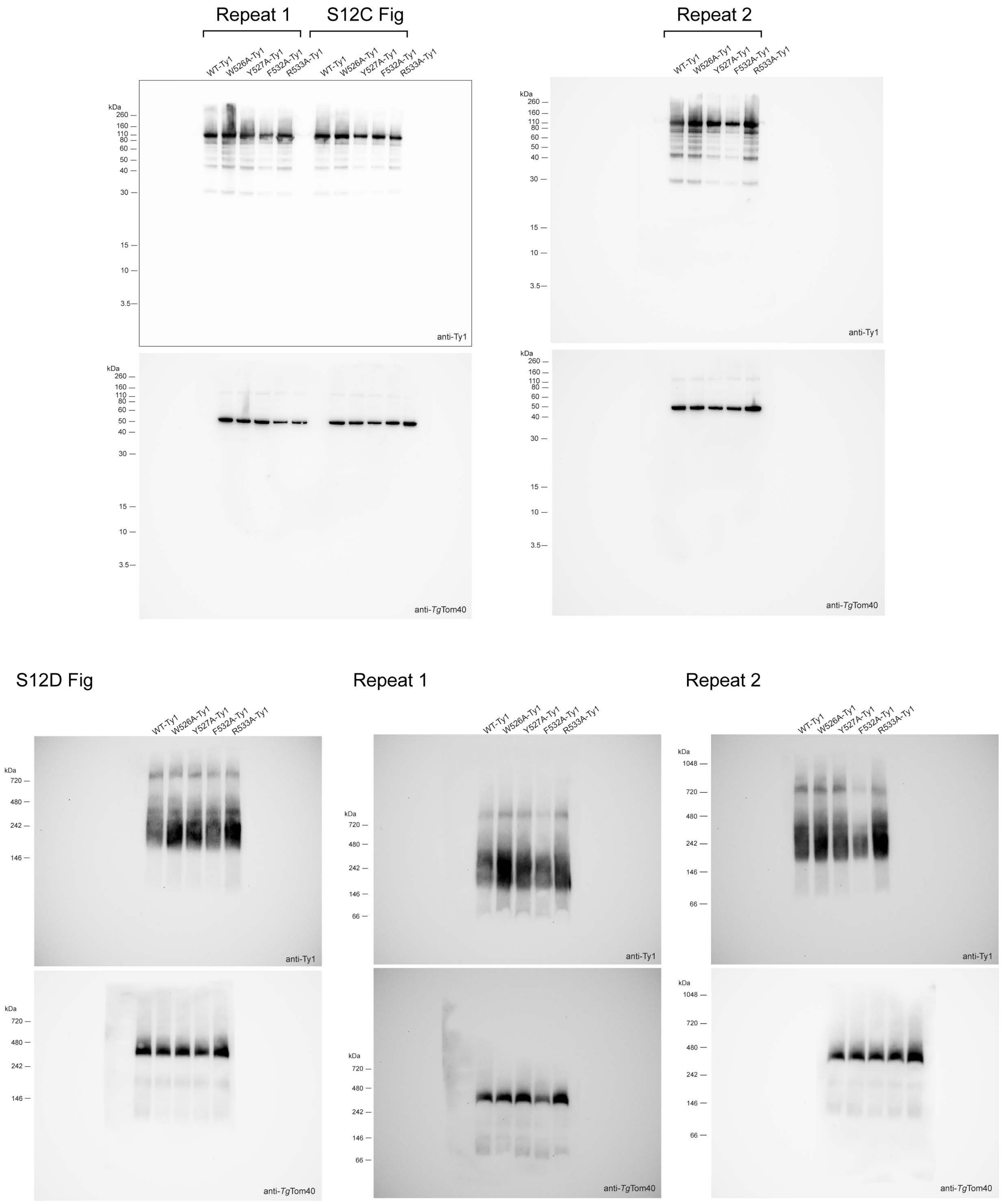

Repeat 1
S12C Fig
Repeat 2
WT-Ty1
W526A-Ty1
Y527A-Ty1
F532A-Ty1
R533A-Ty1
kDa
260
160
110
80
60
50
40
30
15
10
3.5
anti-Ty1
anti-TgTom40
S12D Fig
Repeat 1
Repeat 2
1048
720
480
242
146
66
anti-Ty1
anti-TgTom40
