# Supplementary material for: A novel targeting domain directs essential components of the cytosolic iron–sulfur cluster assembly pathway to the mitochondrion of Toxoplasma parasites
Source: PLoS Biol. 2025 Nov 25;23(11):e3003520. doi: 10.1371/journal.pbio.3003520 (PMC12674569; doi:10.1371/journal.pbio.3003520)
Supplement: S2 Data — Refer to the Figure legends in the manuscript for details on each figure. (PDF) [file pbio.3003520.s017.pdf]

Replicate data from experiments included in the manuscript. Refer to the Figure legends in the manuscript for details on each figure.

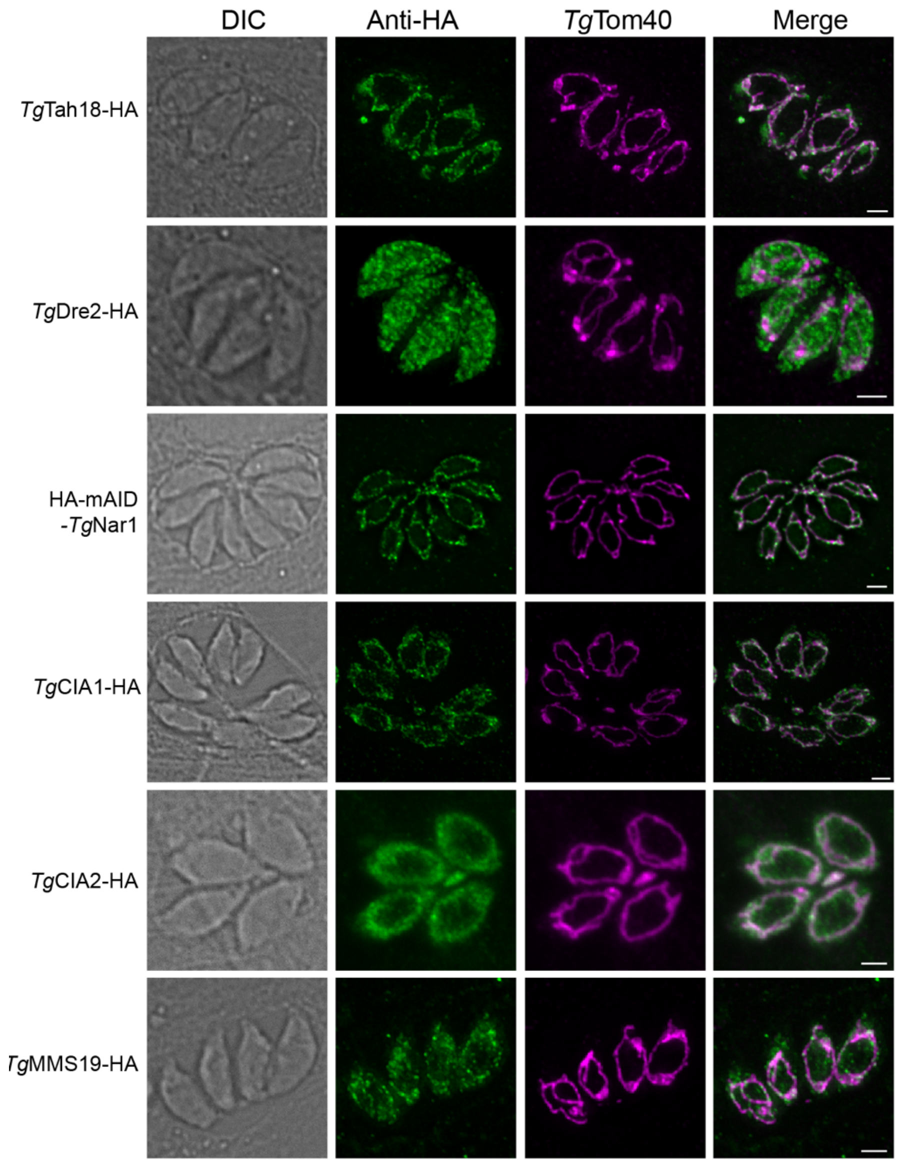

Figure 1 replicate data. Note that the fluorescence images for the *Tg*Tah18-HA, *Tg*Dre2-HA, *Tg*CIA1-HA, and *Tg*MMS19-HA expressing parasites were deconvolved using SoftWorx Suite 2.0 software.

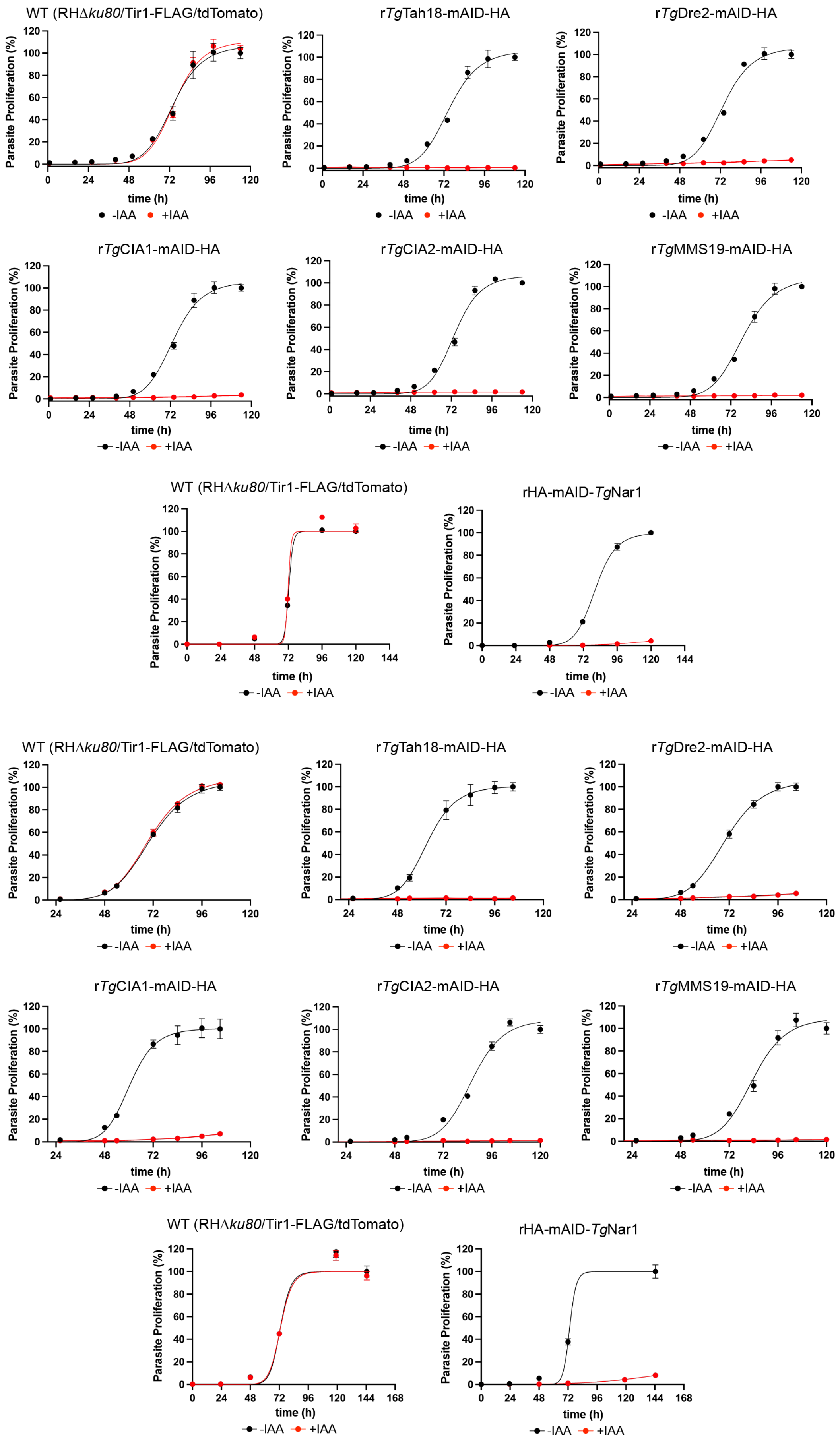

Figure 2 replicate data.

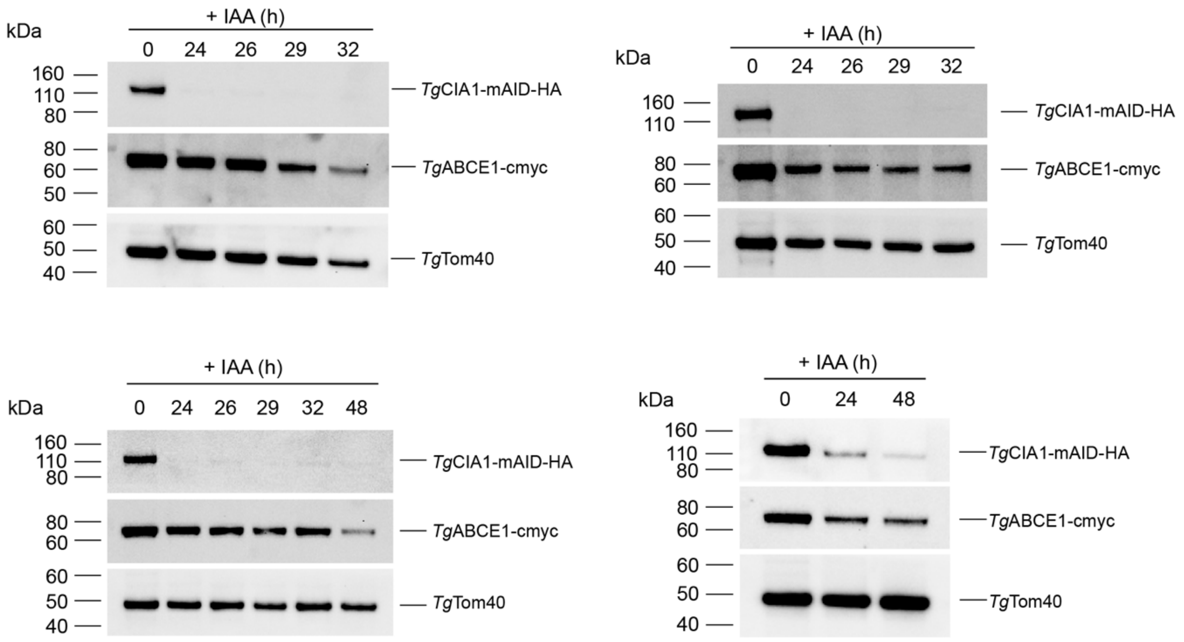

Figure 3A replicate data.

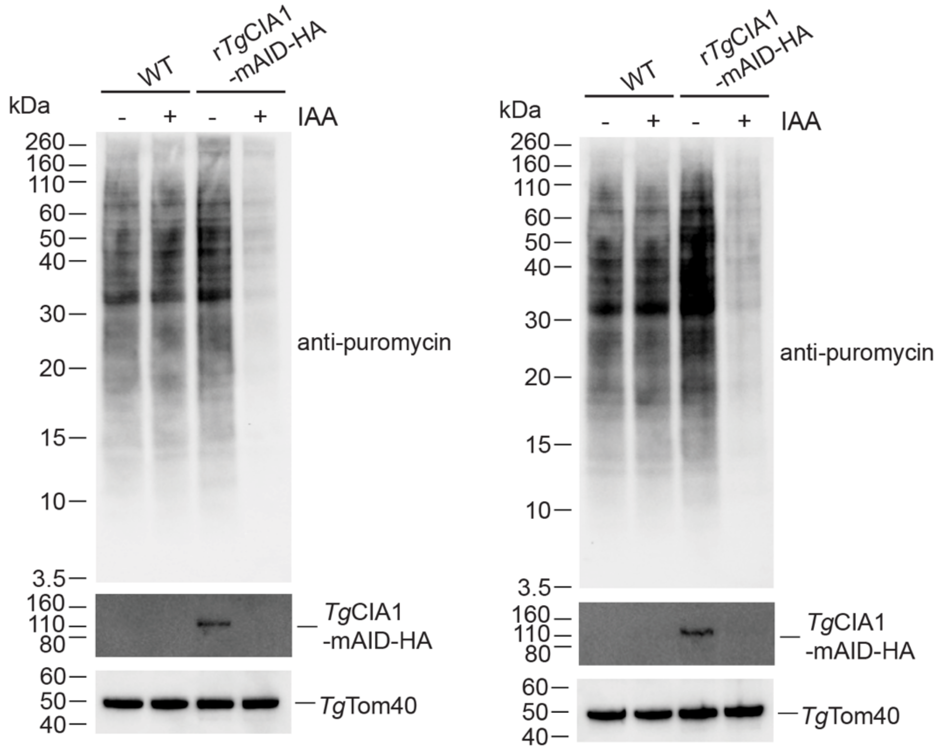

Figure 3C replicate data.

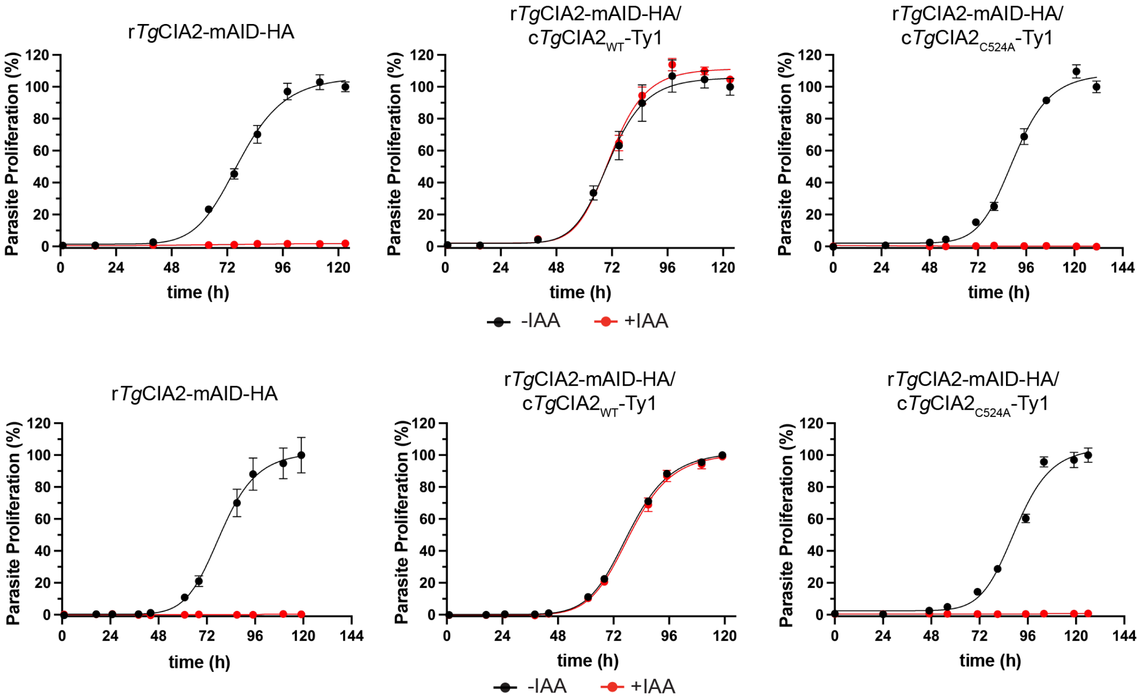

Figure 3G replicate data.

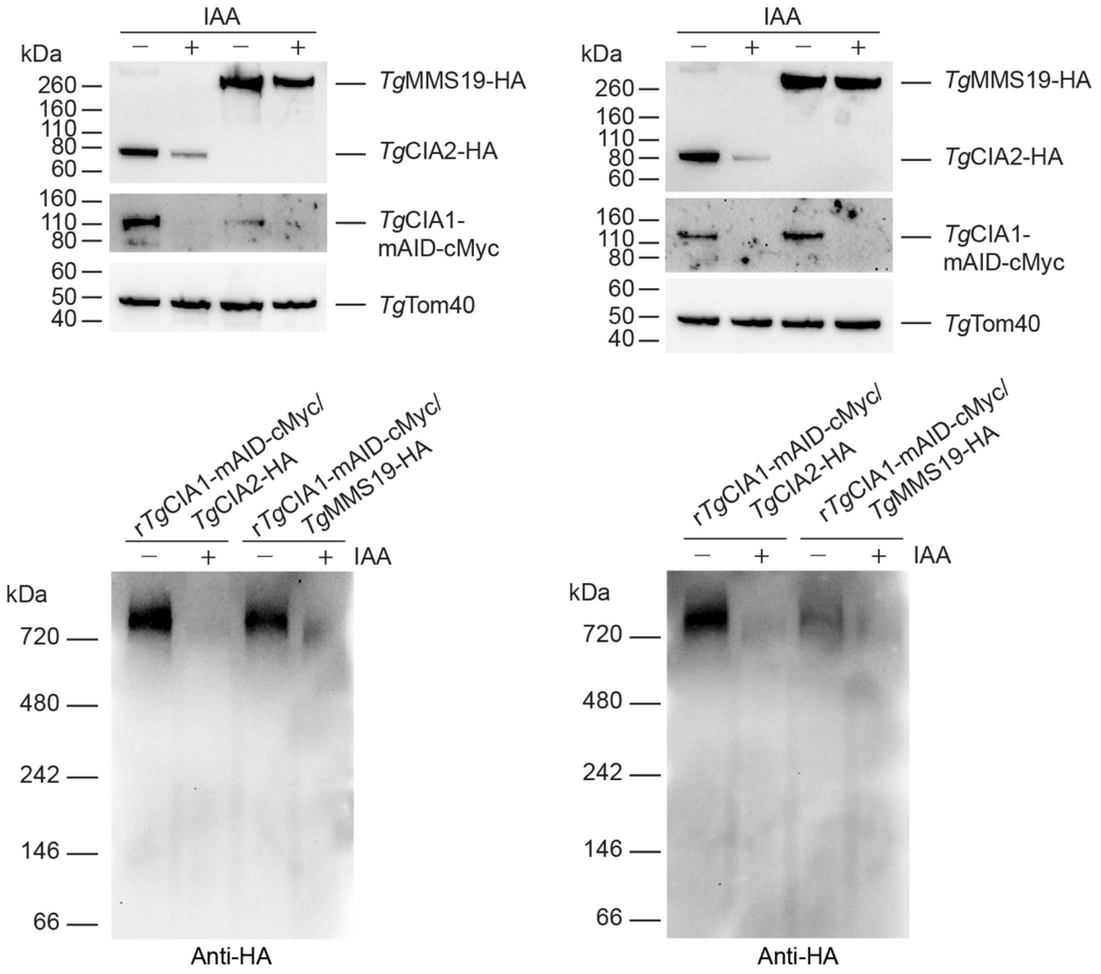

Figure 4B (top) and Figure 4D (bottom) replicate data.

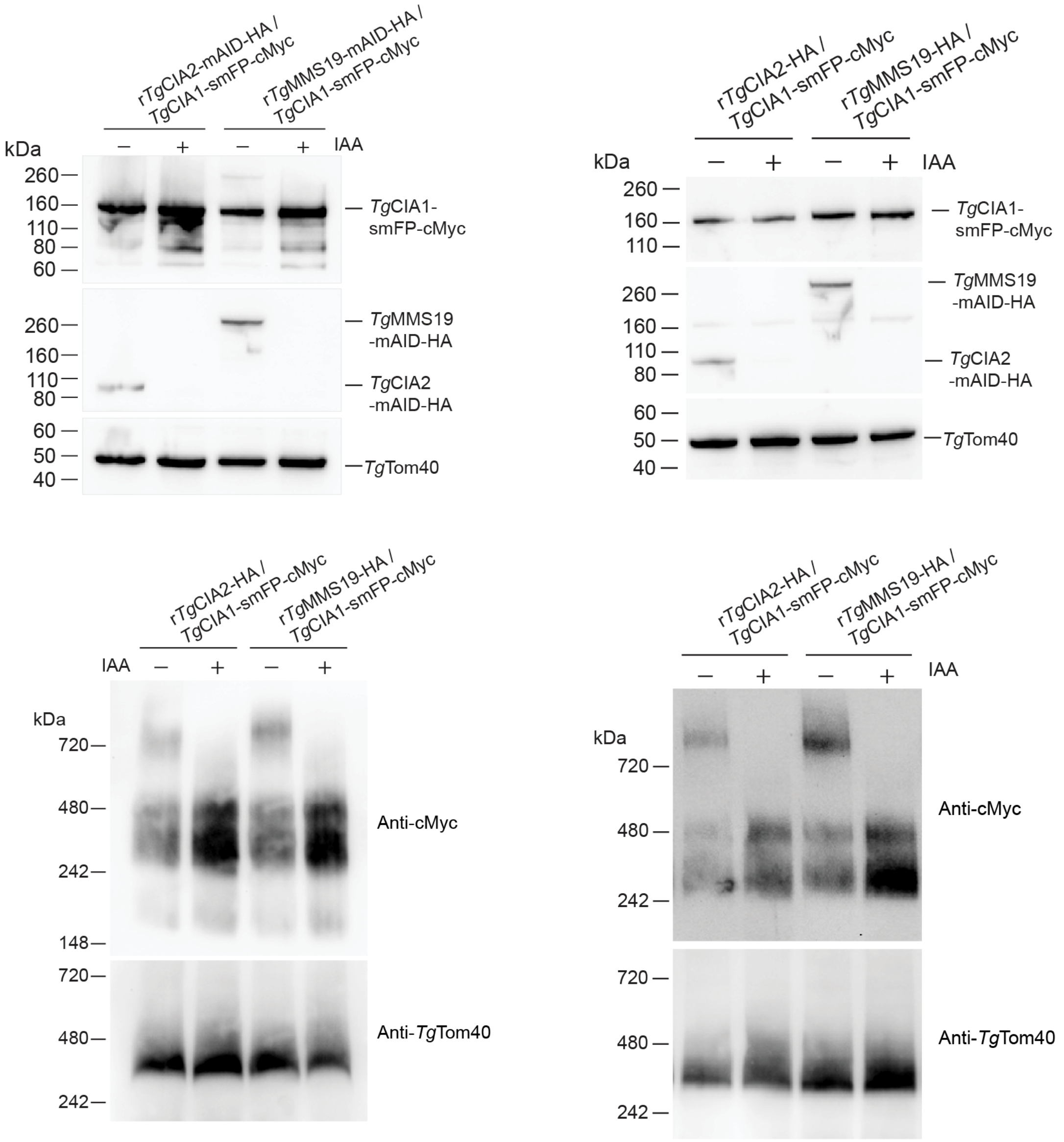

Figure 4E (top) and Figure 4F (bottom) replicate data.

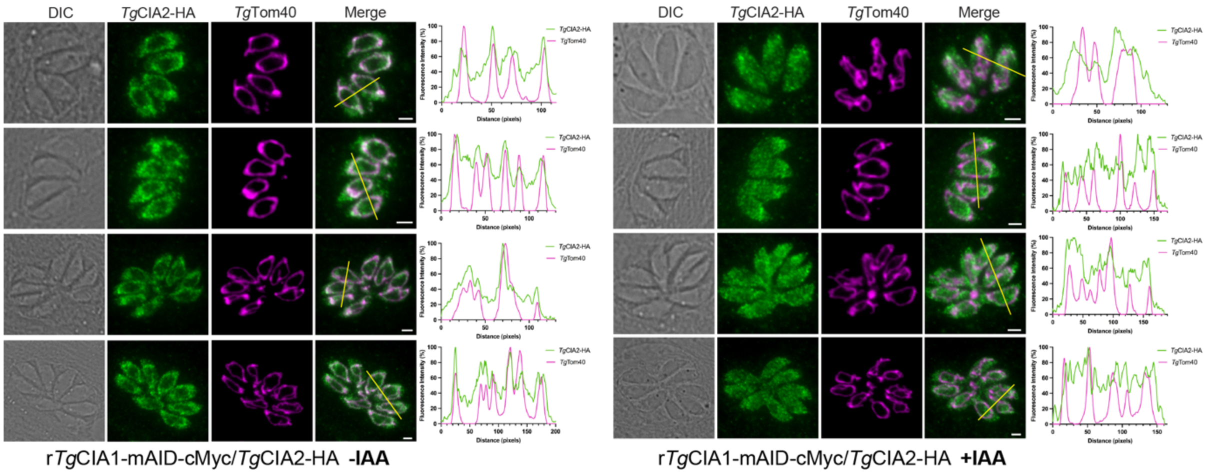

Figure 5A replicate data.

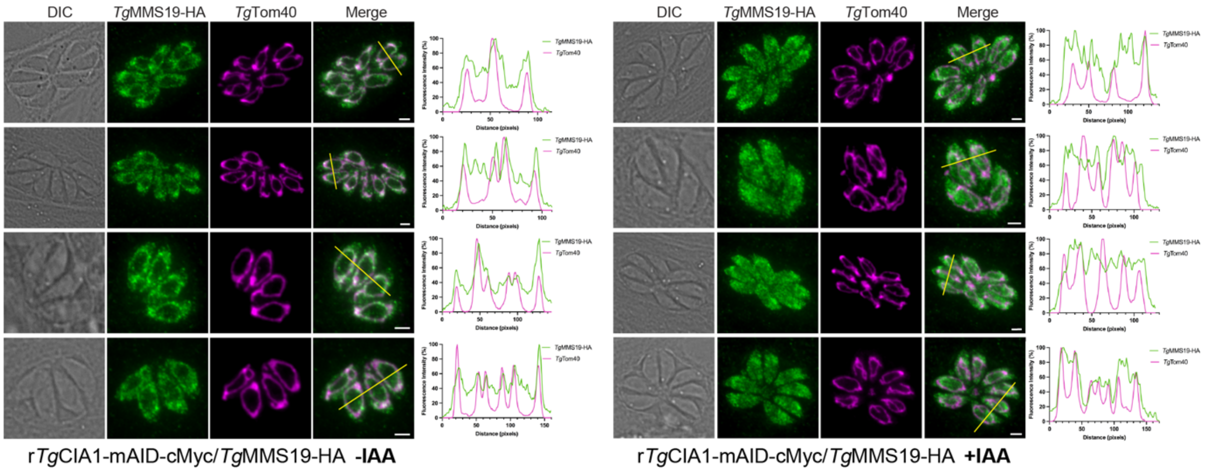

Figure 5B replicate data.

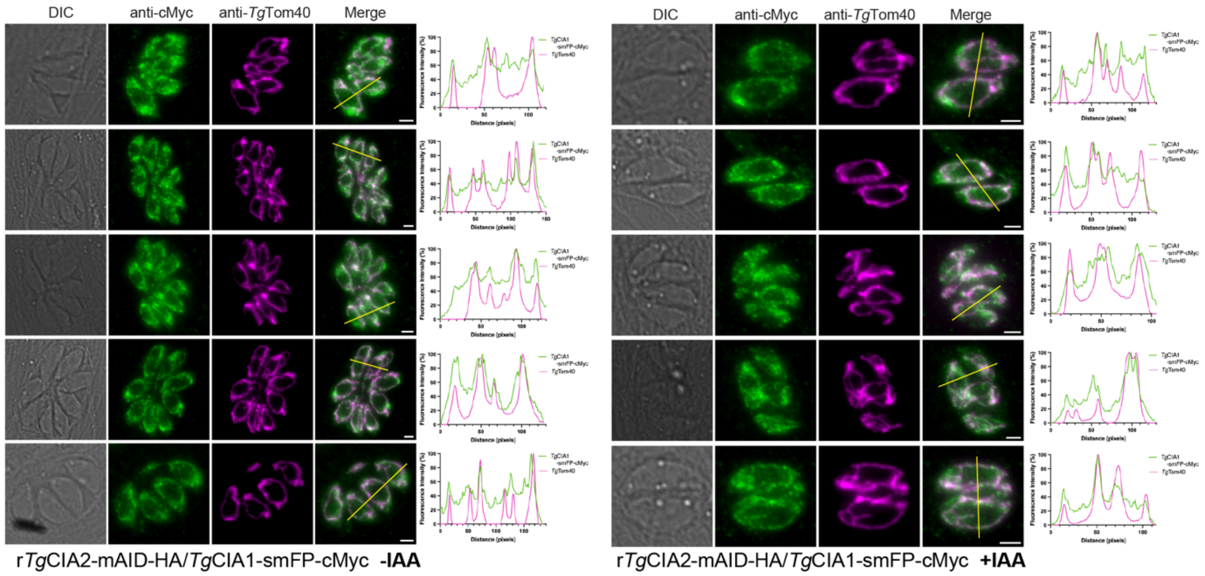

Figure 5C replicate data.

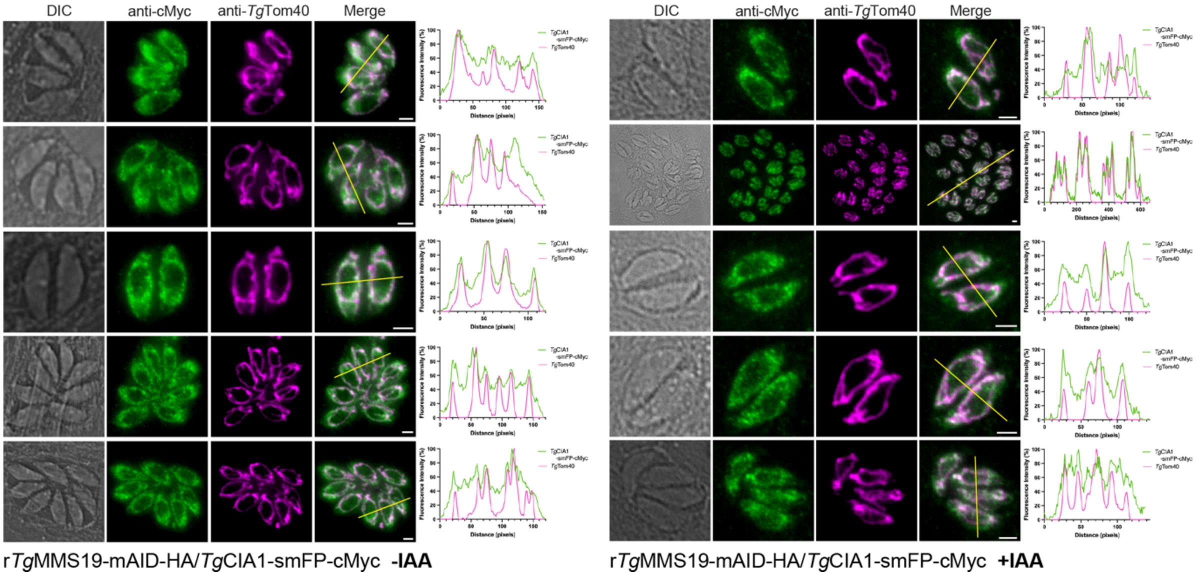

Figure 5D replicate data.

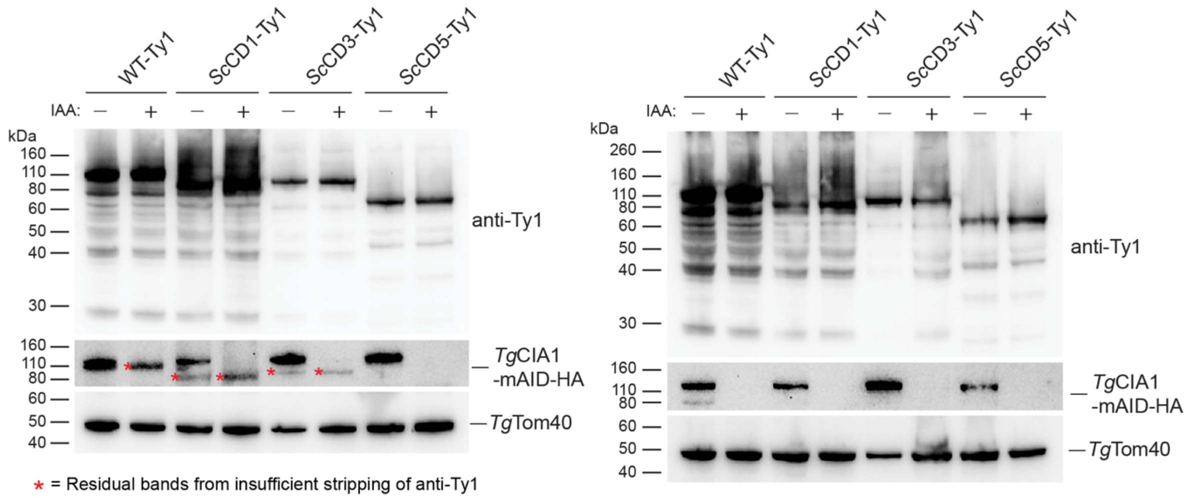

Figure 6B replicate data.

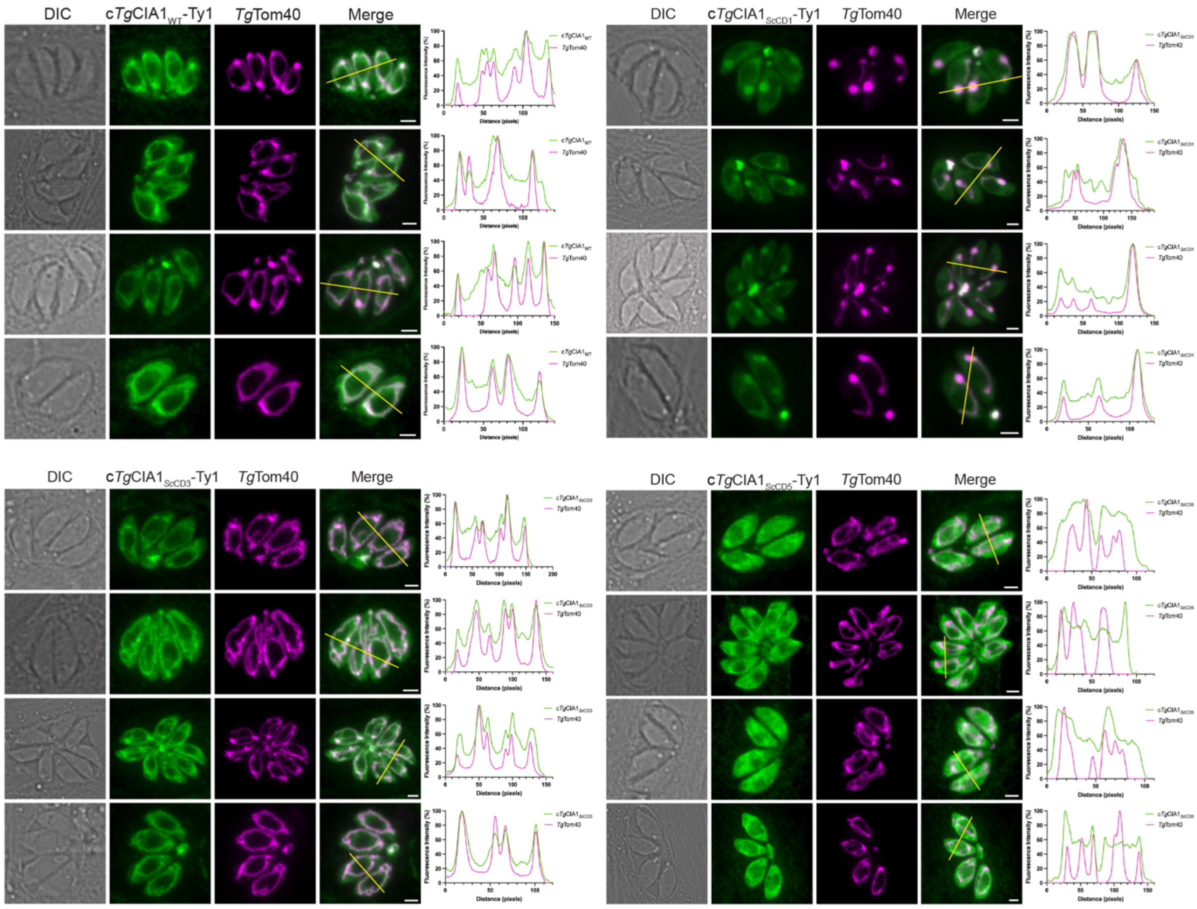

Figure 6C replicate data.

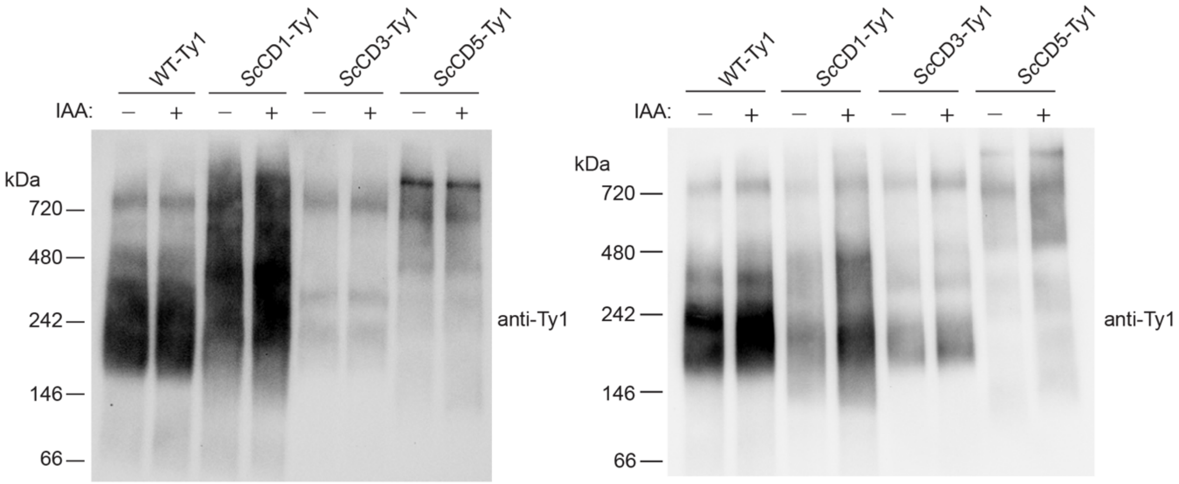

Figure 6E replicate data.

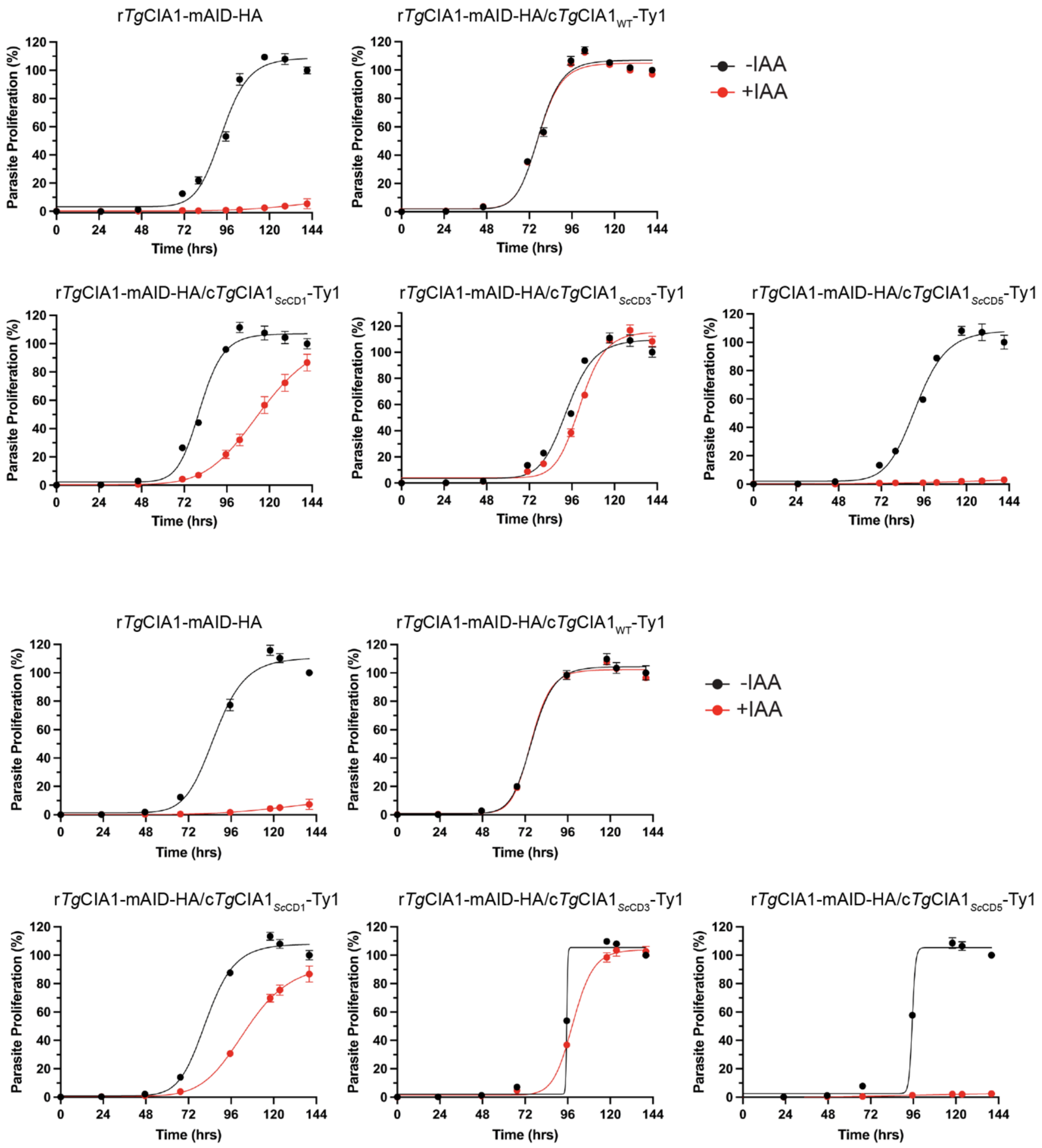

Figure 6F replicate data.

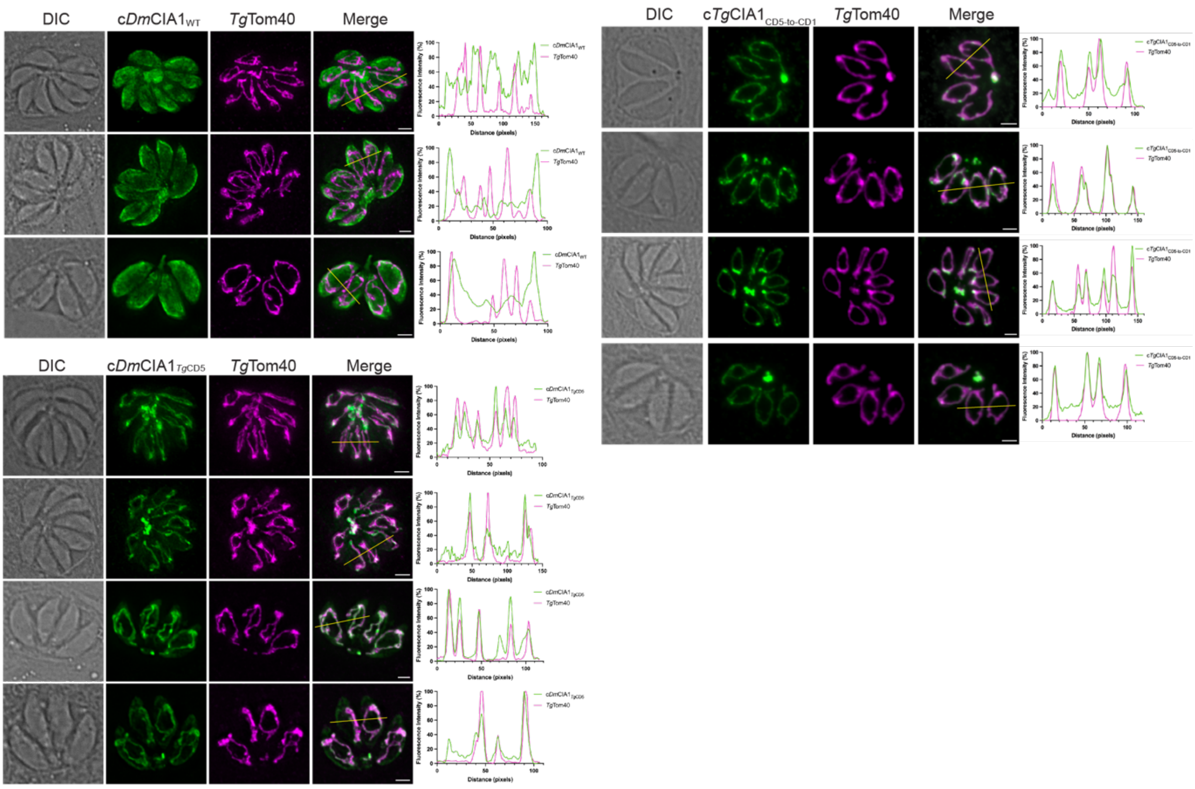

Figure 7A (left) and Figure 7B (right) replicate data.

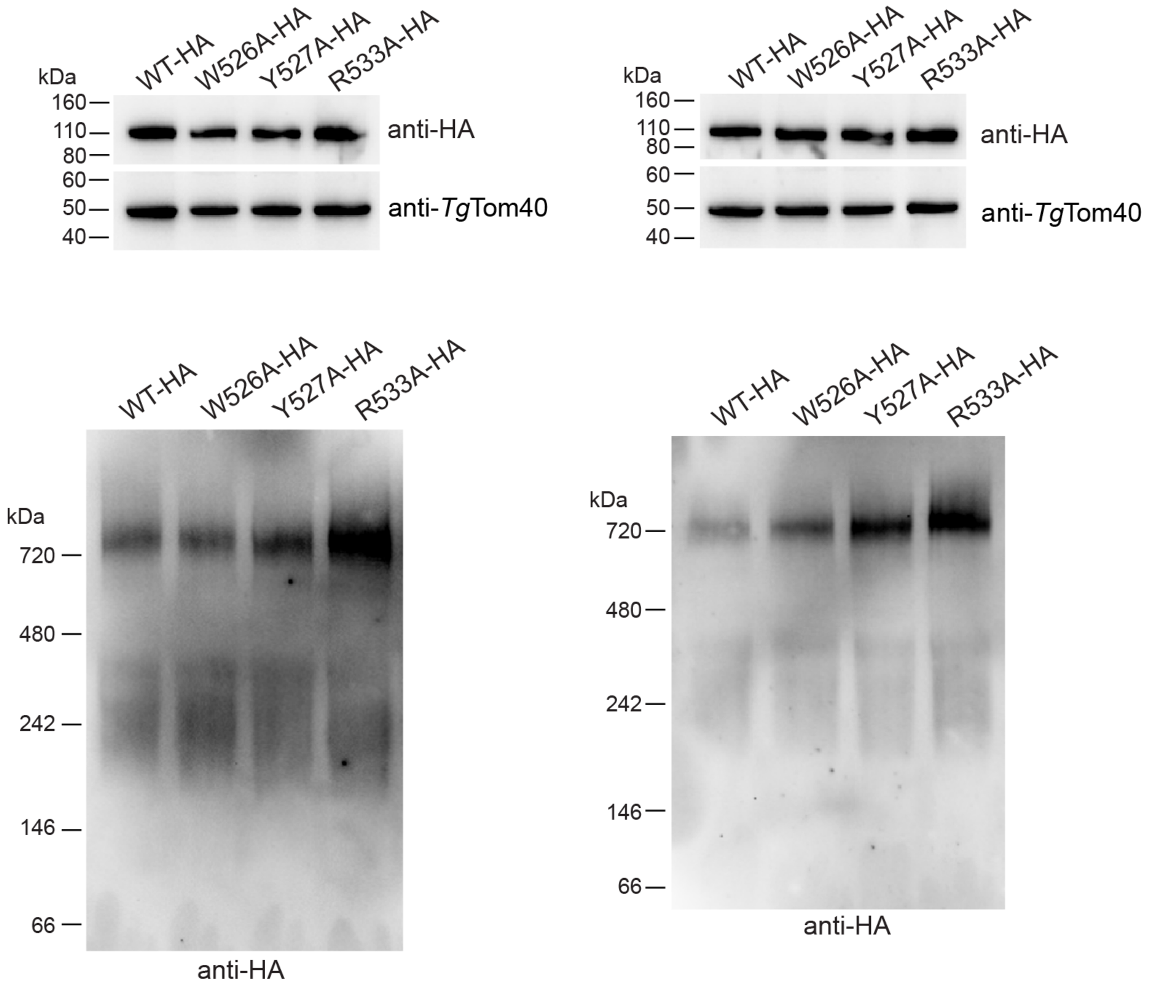

Figure 8B (top) and Figure 8C (bottom) replicate data.

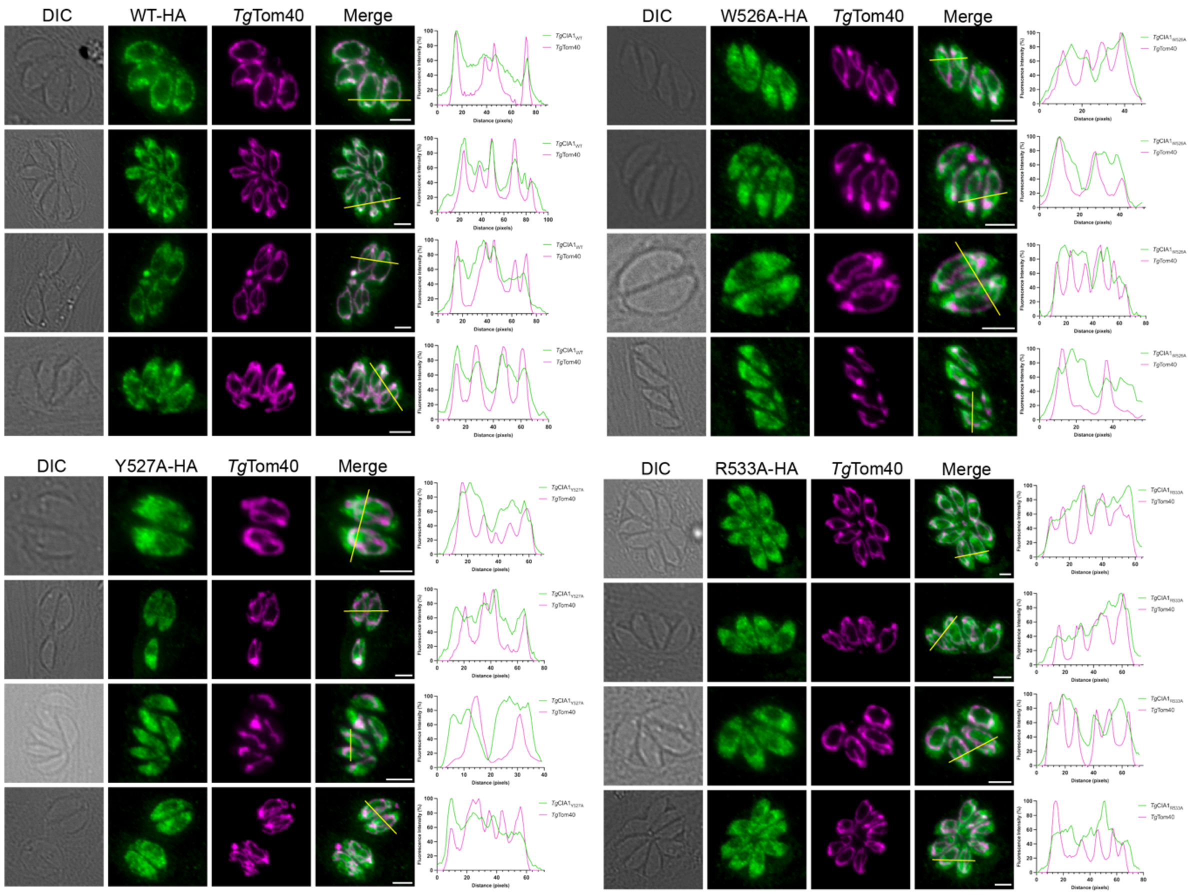

Figure 8D replicate data.

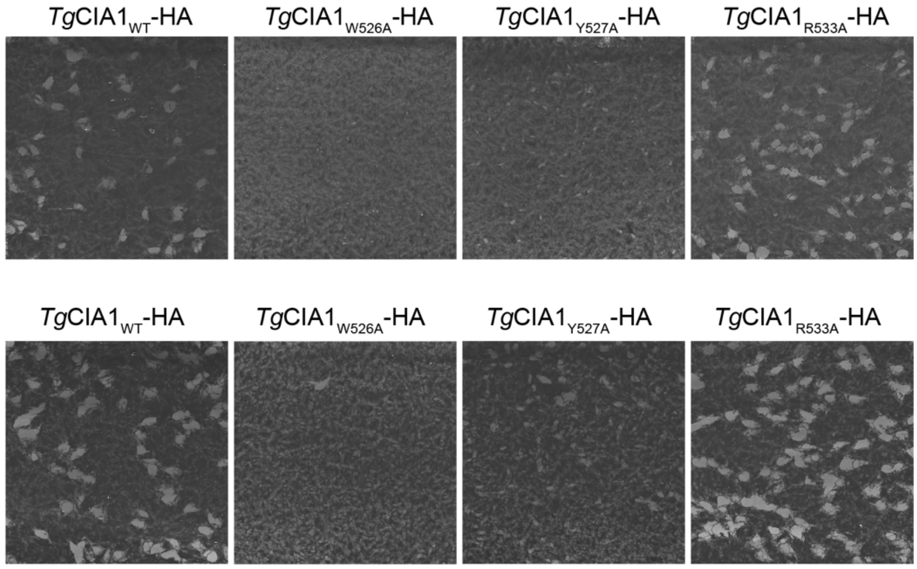

Figure 8F replicate data.

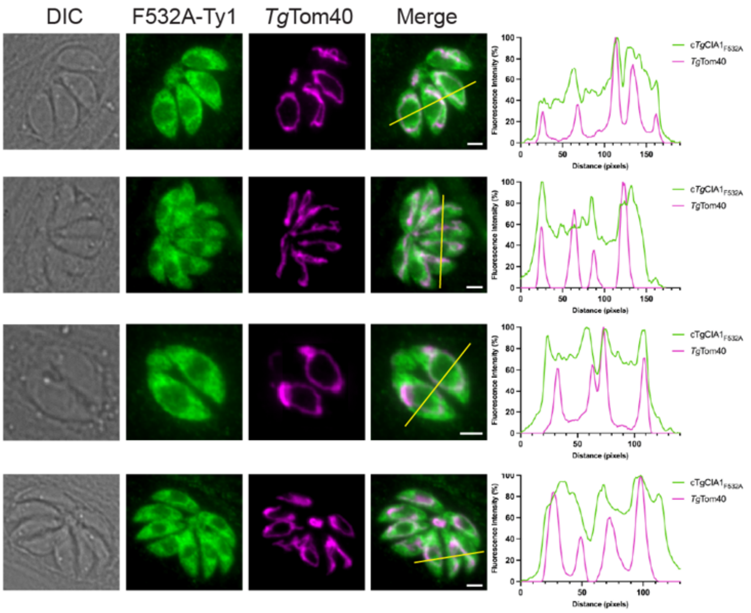

Figure 8G replicate data.

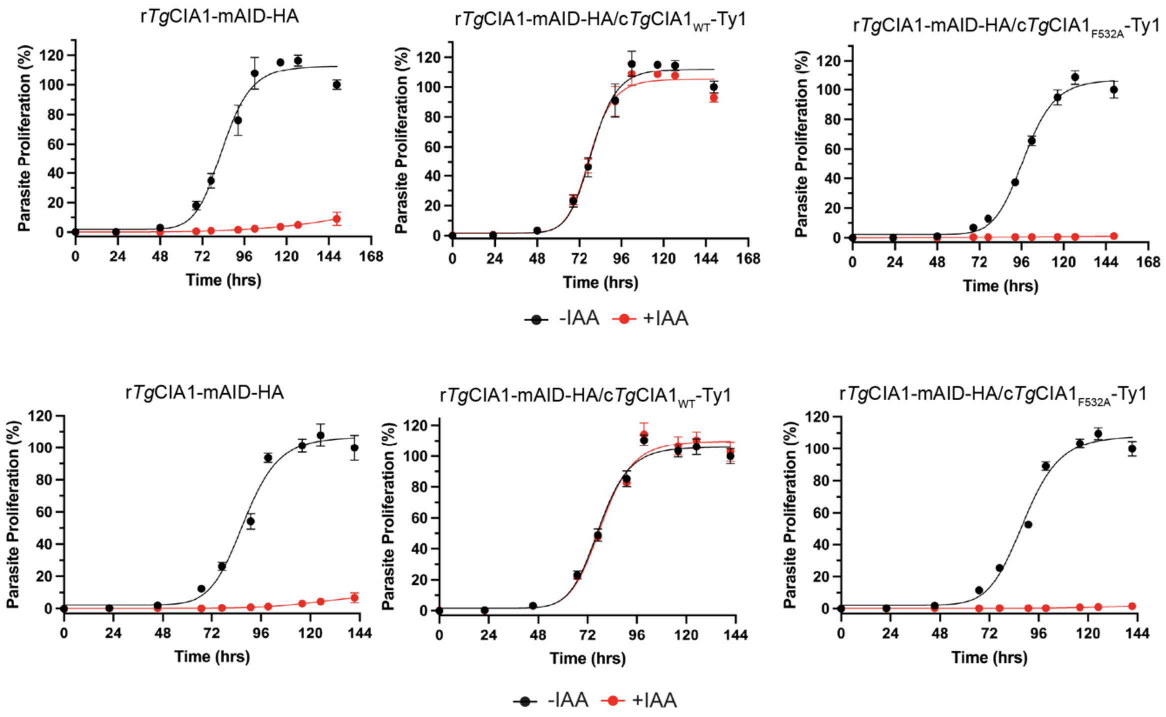

Figure 8I replicate data.

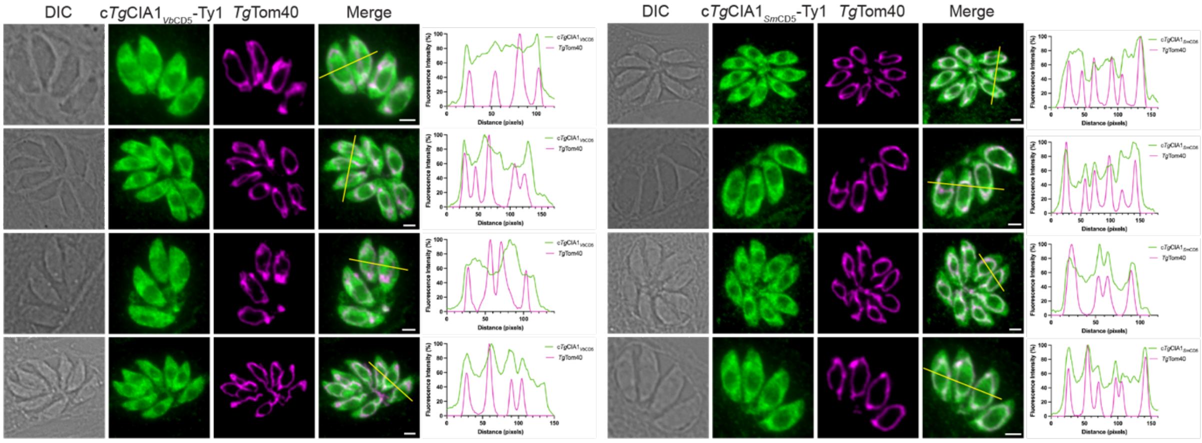

Figure 9A (left) and Figure 9B (right) replicate data.

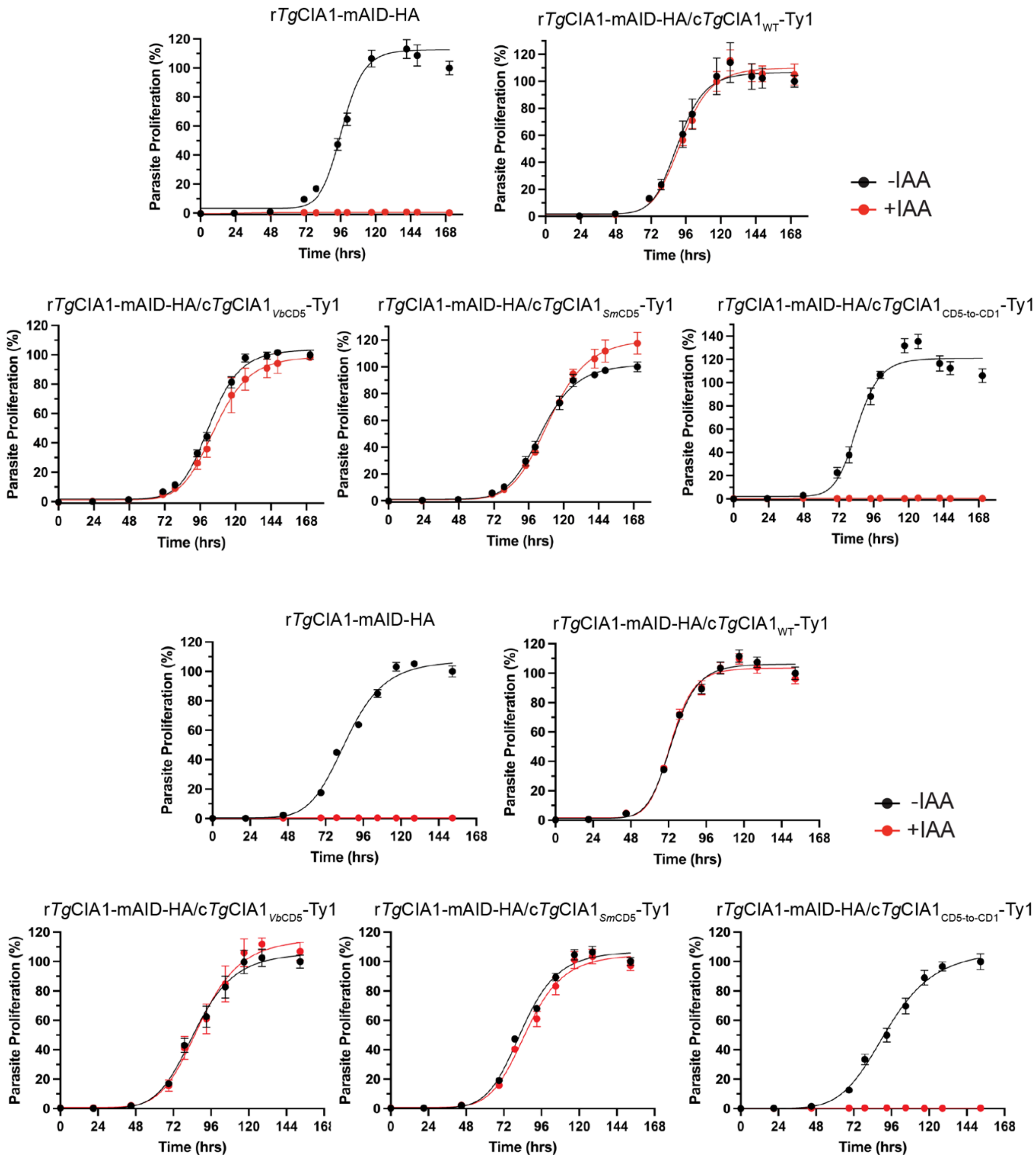

Figure 7C and Figure 9D replicate data.

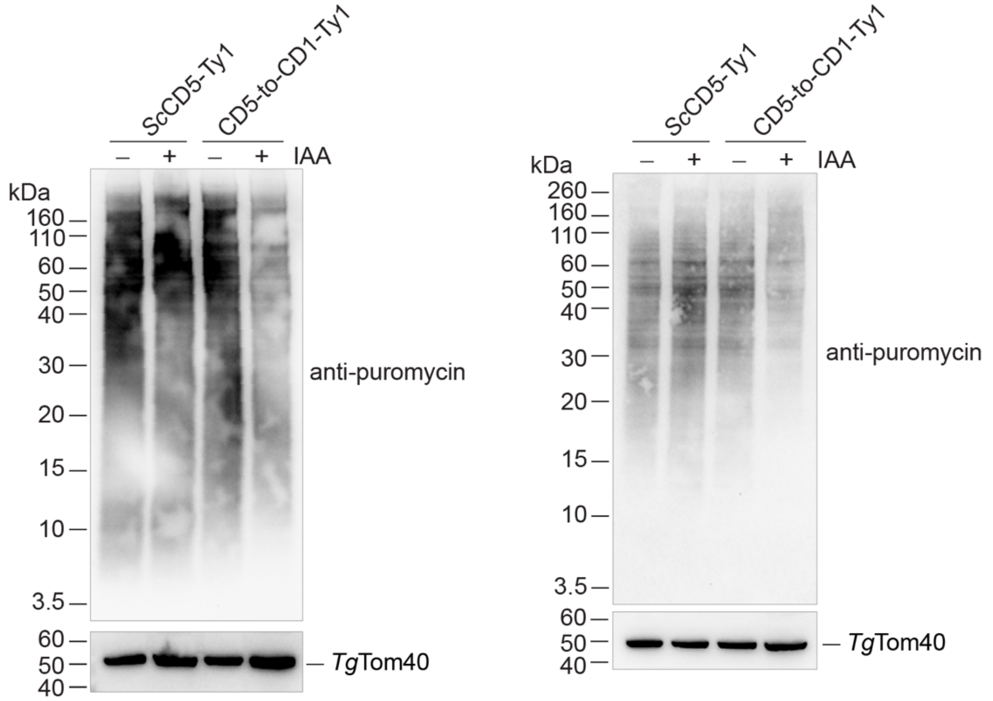

Figure 10B replicate data.

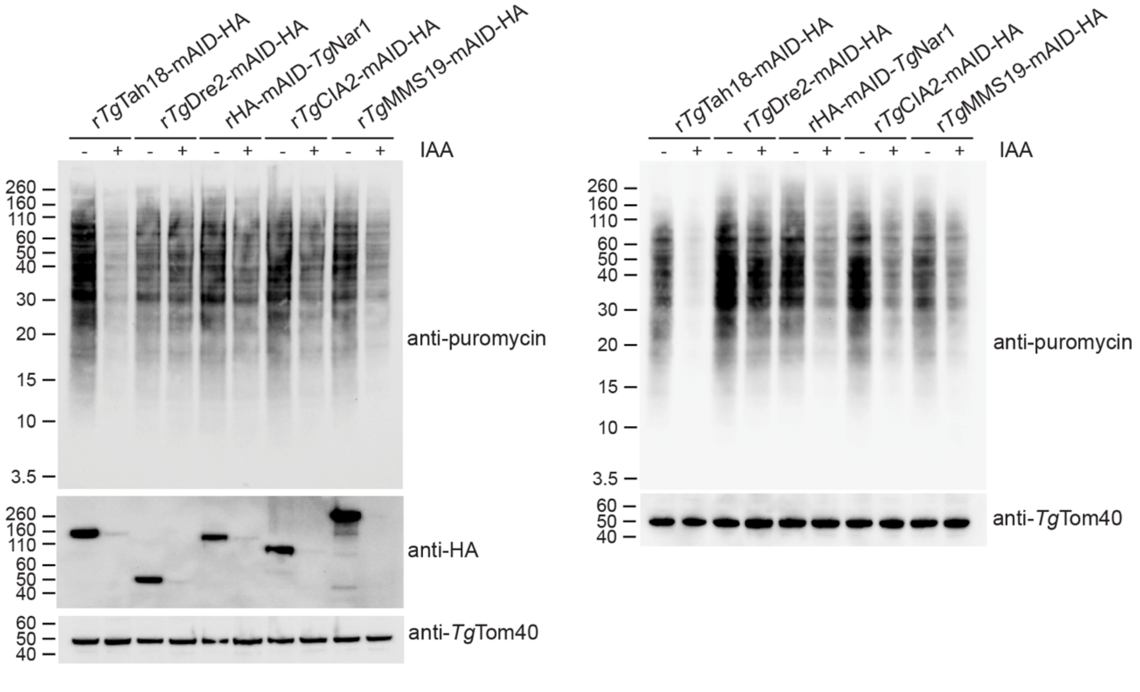

S4A Figure replicate data.

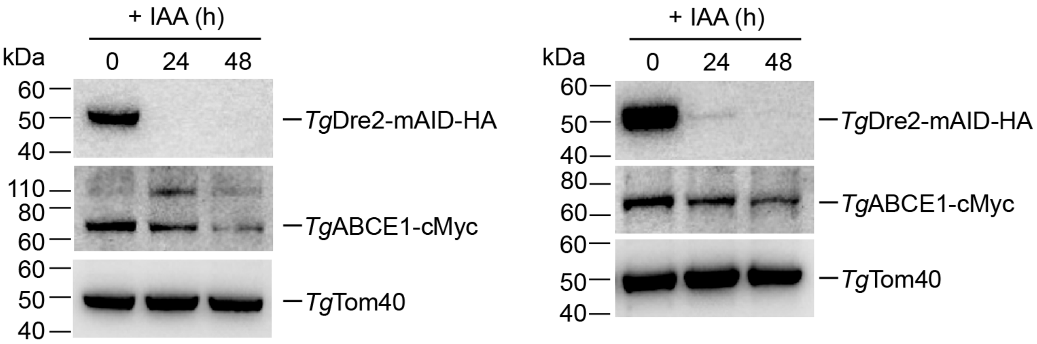

S4C Figure replicate data.

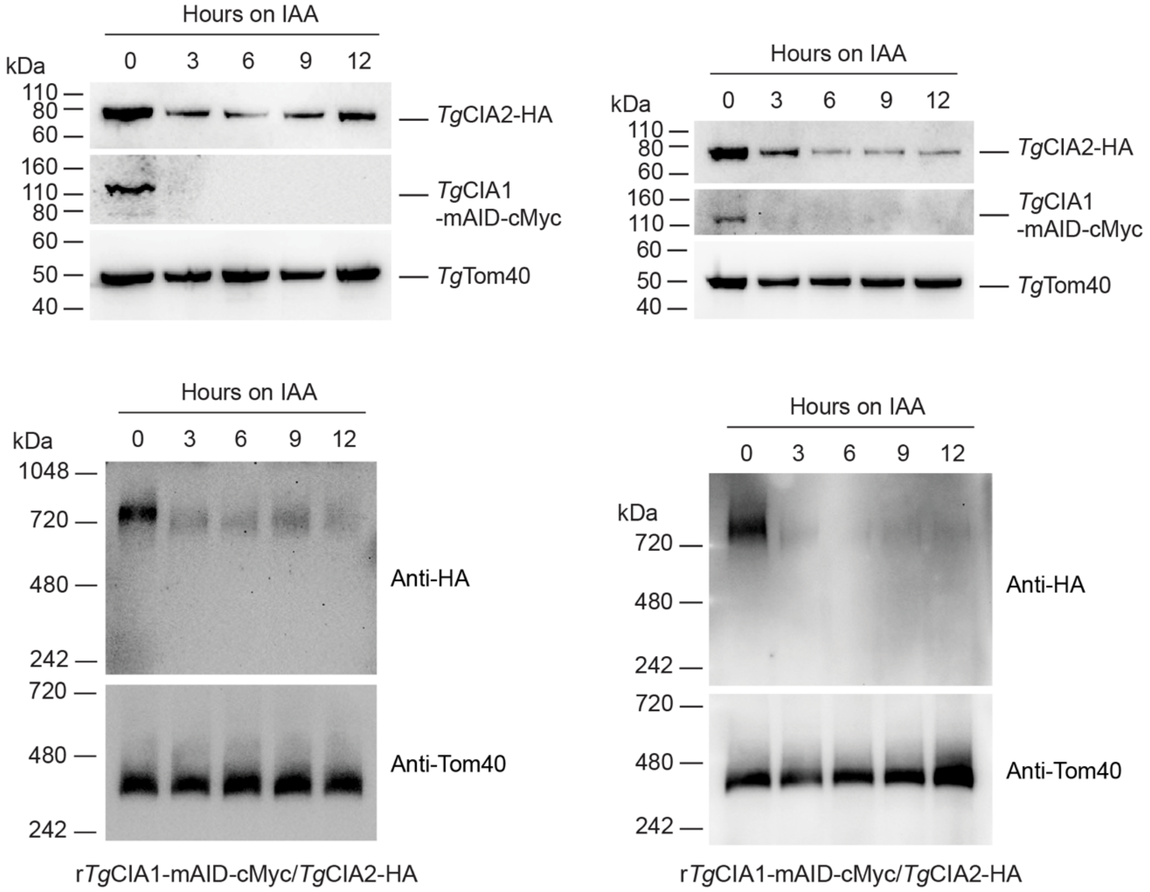

S6B Figure (top) and S6C Figure (bottom) replicate data.

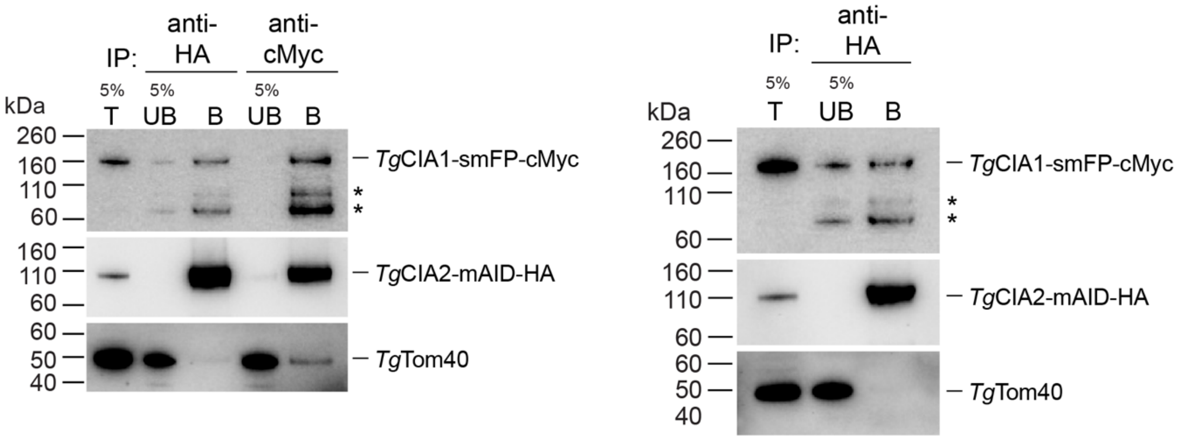

S6D Figure replicate data.

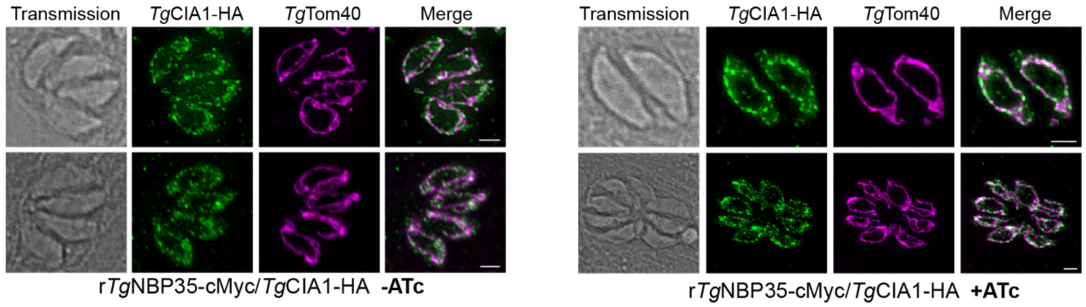

S8A Figure replicate data. Images were deconvolved using SoftWorx Suite 2.0 software.

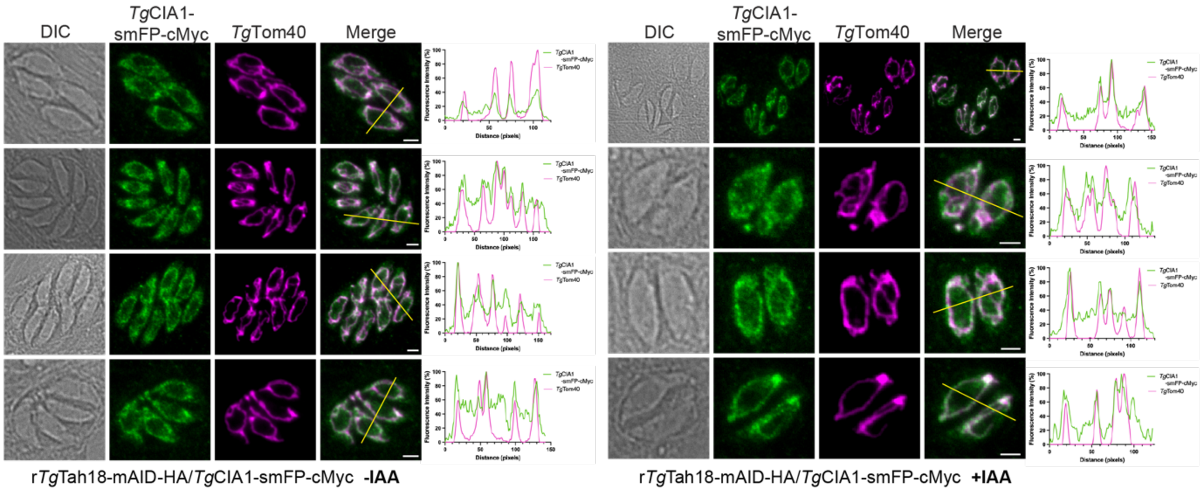

S8C Figure replicate data.

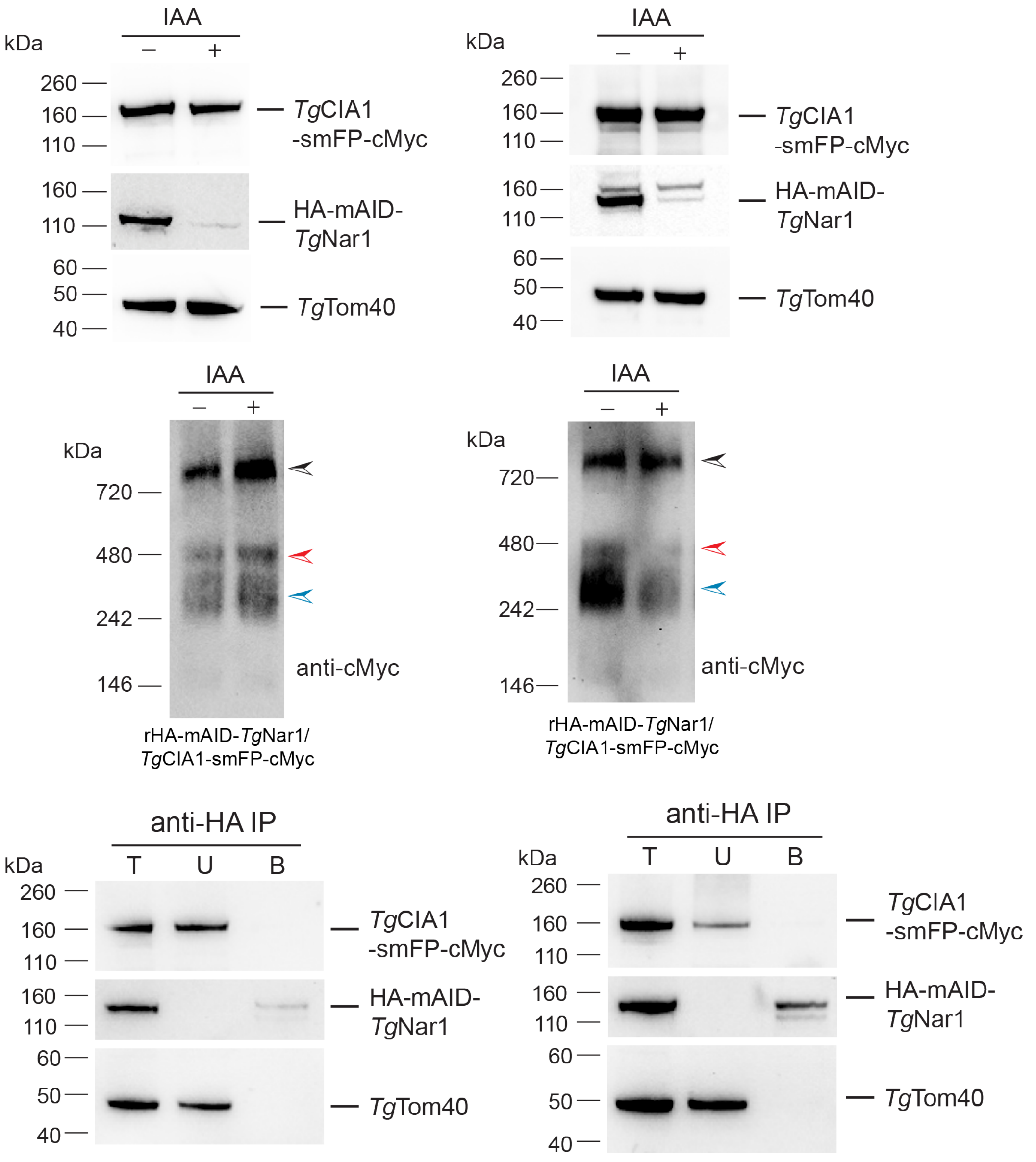

S8E Figure (top), S8F Figure (middle), and S8G Figure (bottom) replicate data.

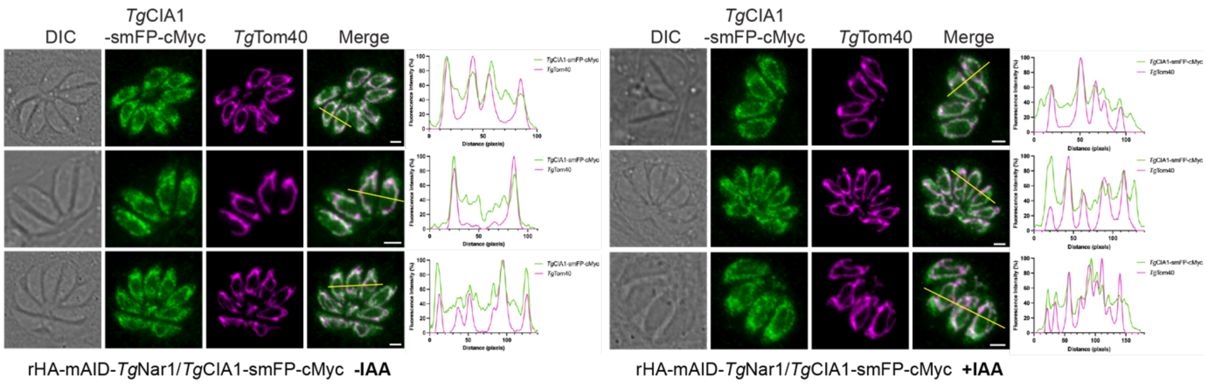

S8H Figure replicate data.

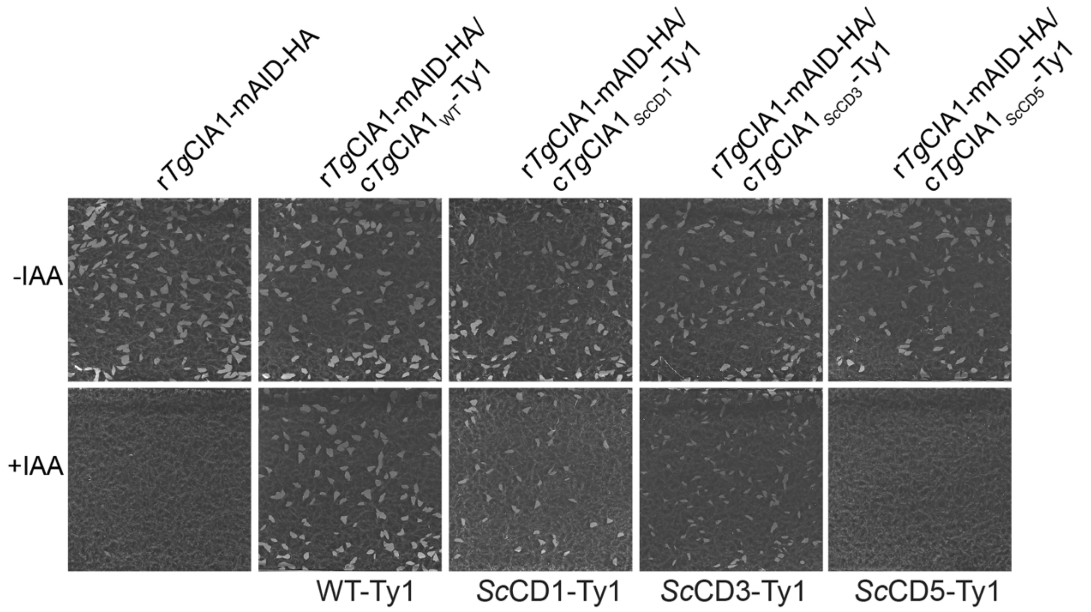

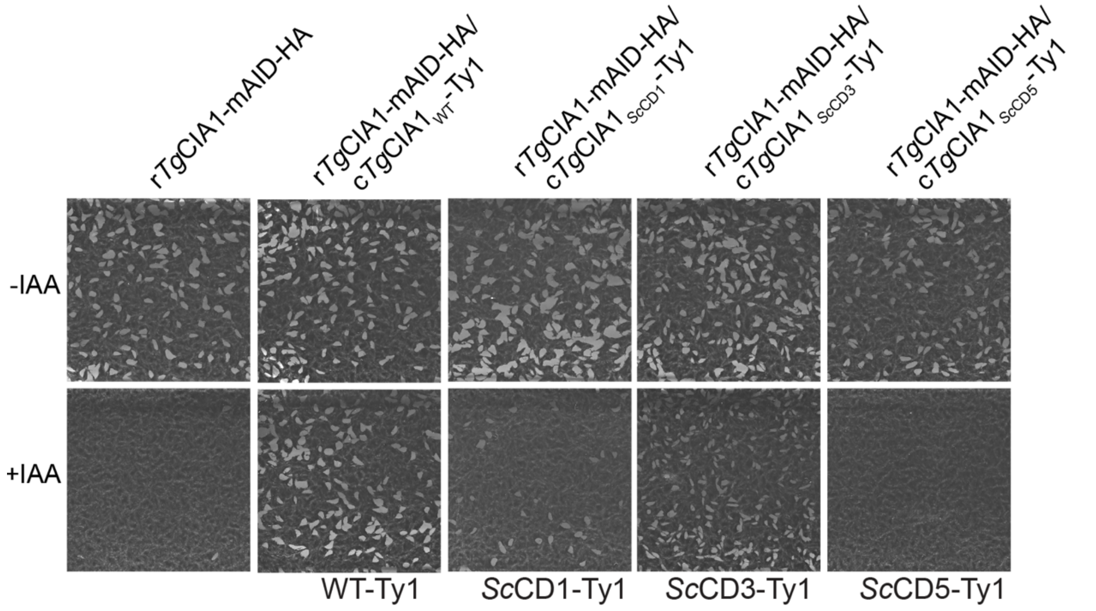

S10C Figure replicate data.

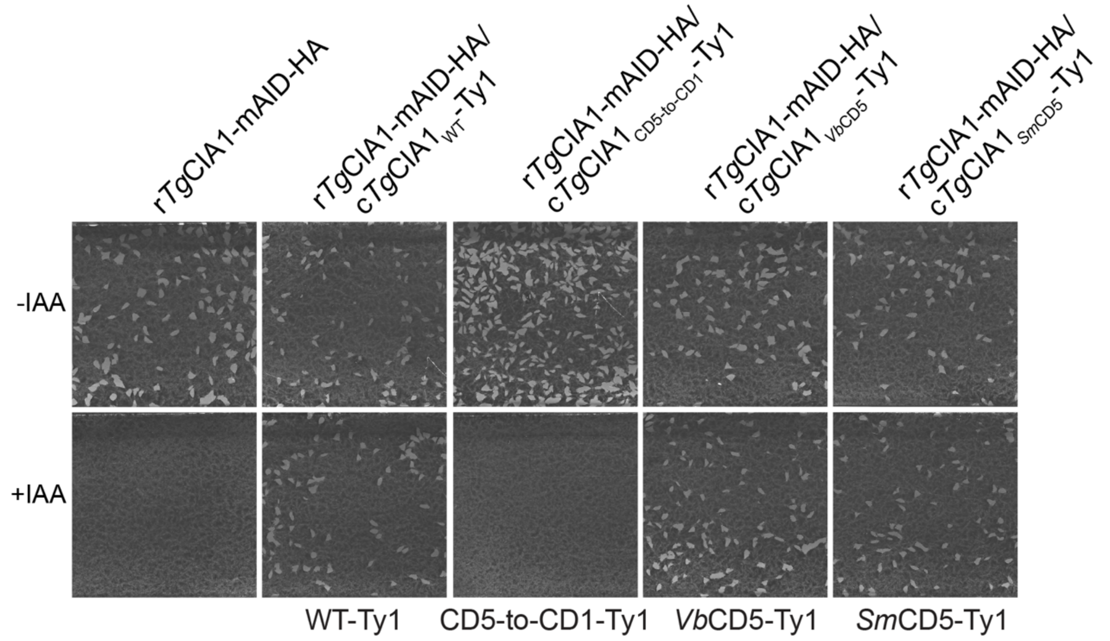

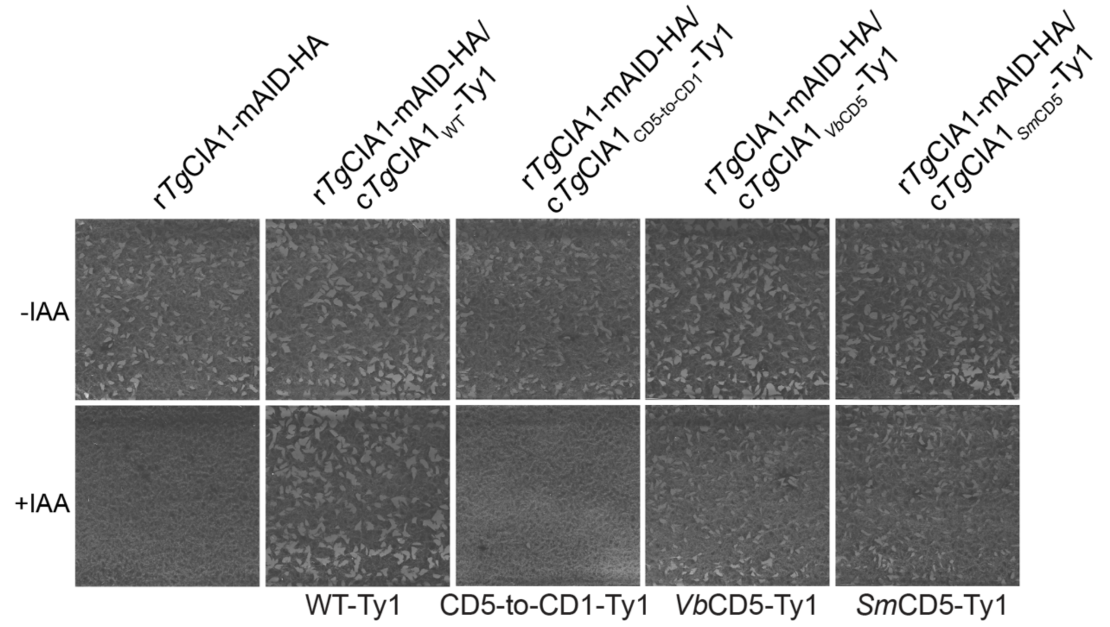

S10D Figure replicate data.

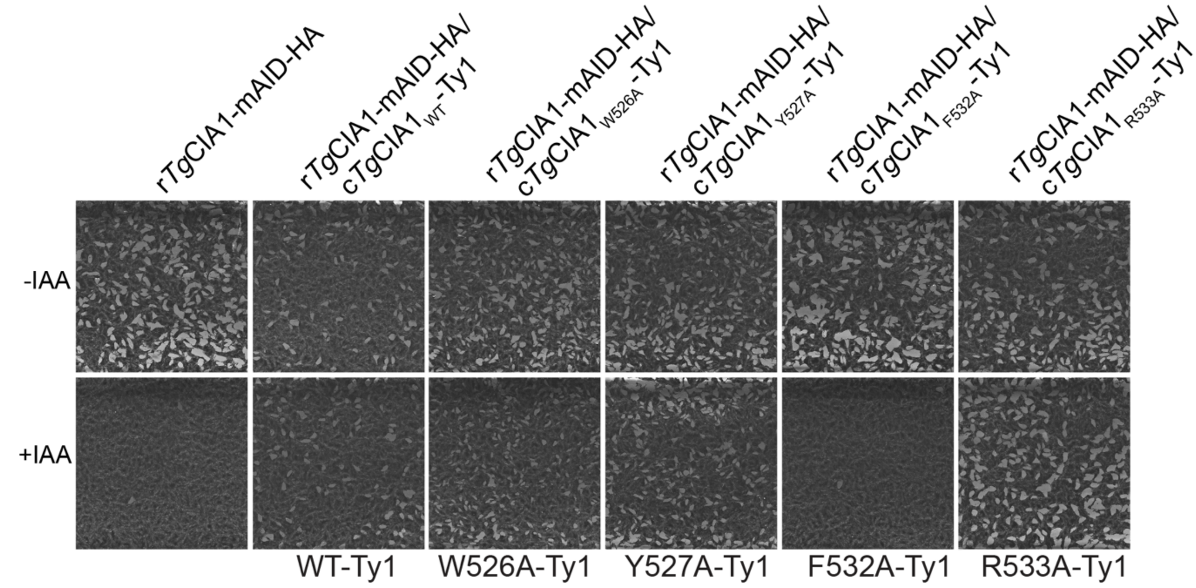

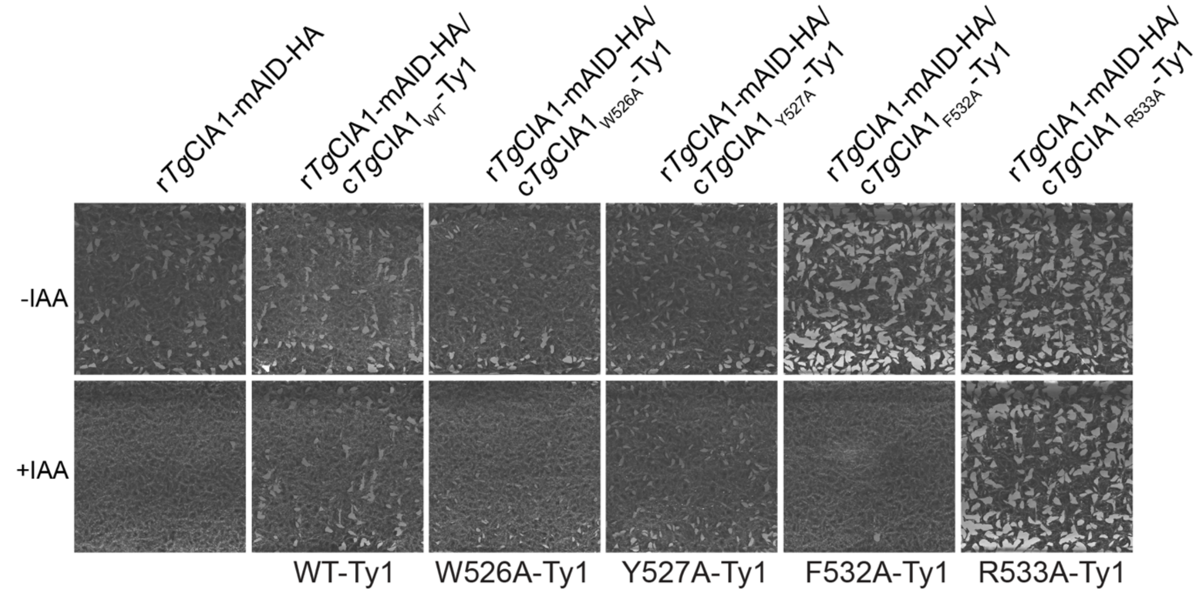

S10E Figure replicate data.

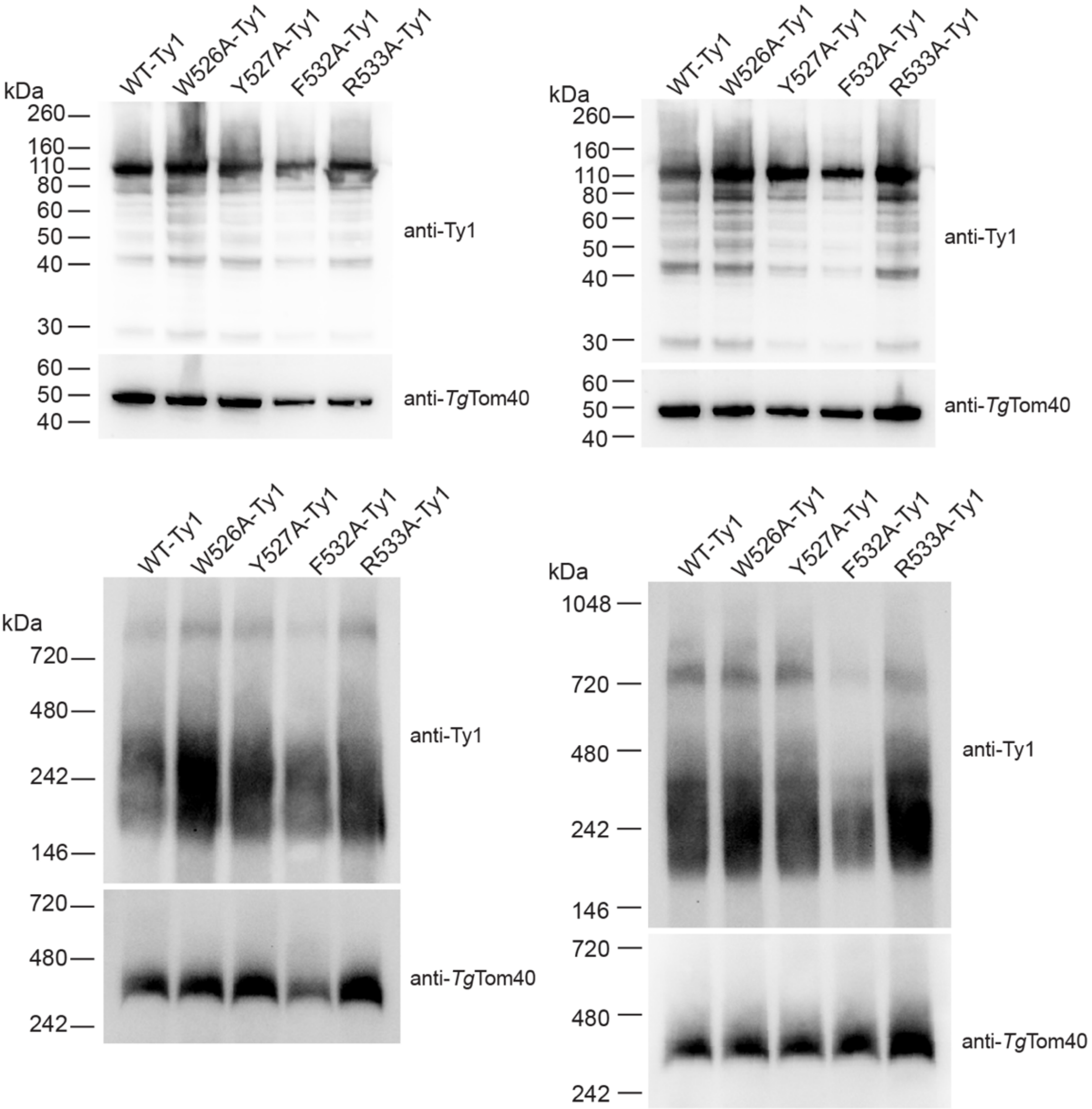

S12C Figure (top) and S12D Figure (bottom) replicate data.

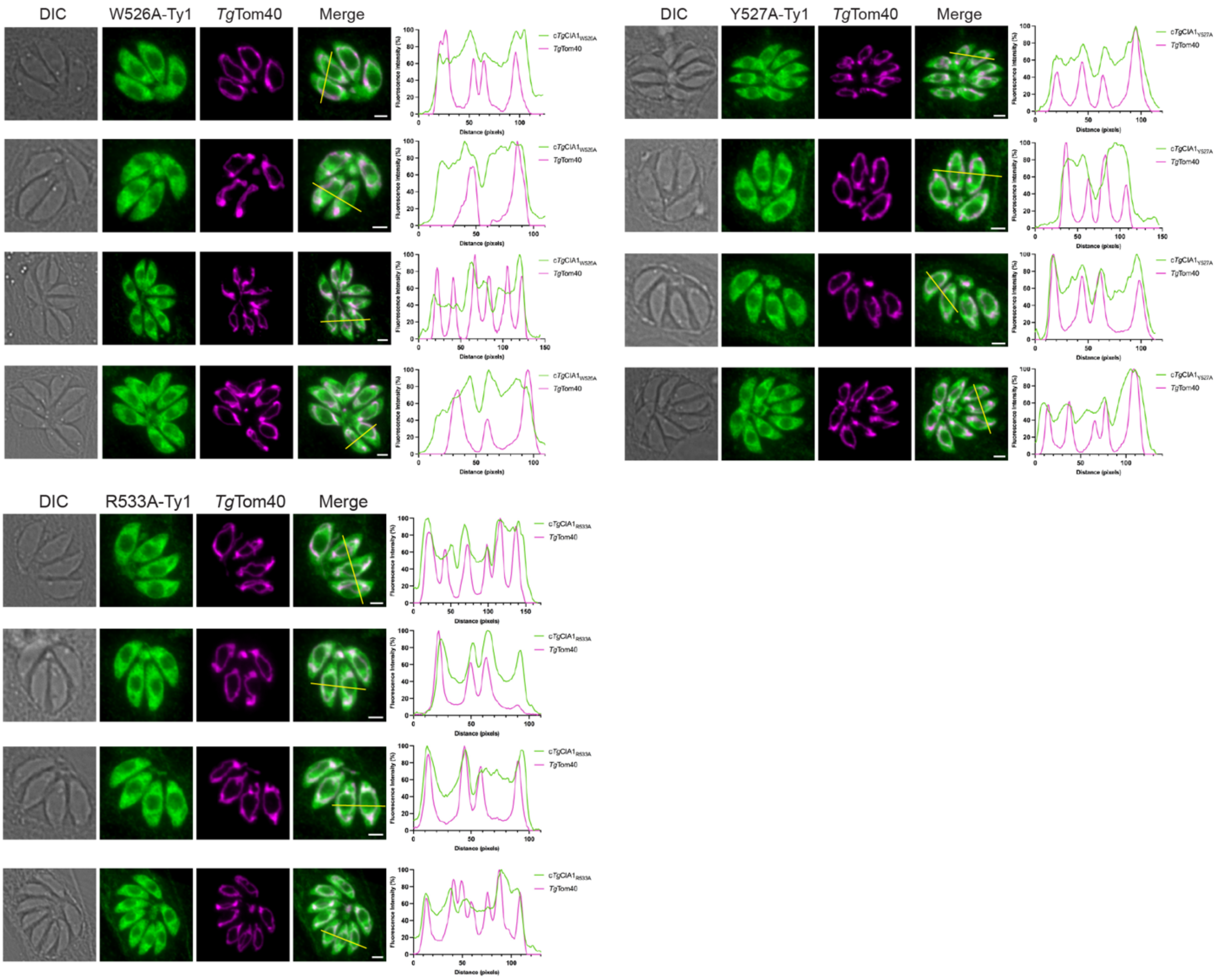

S12E Figure replicate data.

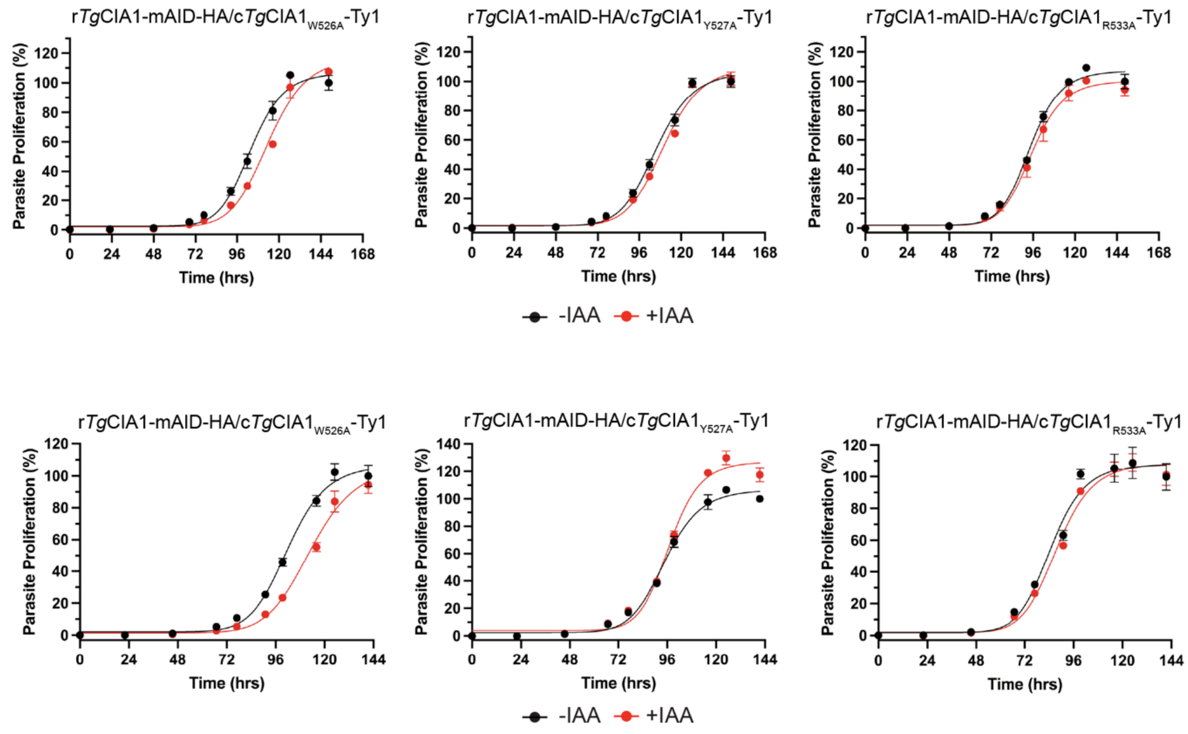

S12G Figure replicate data.
