# Supplementary material for: A novel targeting domain directs essential components of the cytosolic iron–sulfur cluster assembly pathway to the mitochondrion of Toxoplasma parasites
Source: PLoS Biol. 2025 Nov 25;23(11):e3003520. doi: 10.1371/journal.pbio.3003520 (PMC12674569; doi:10.1371/journal.pbio.3003520)
Supplement: Attachment — Submitted filename: Response to Reviewers.pdf [file pbio.3003520.s018.pdf]

Response to reviewers

We are grateful to the editor and reviewers for their thoughtful feedback and criticism on the original version of the manuscript. We have responded to each of the points raised in the reviewer feedback, with reviewer/editor comments in black text, our responses in blue text, and have noted key changes to the manuscript text in red italic text. We note the new or modified figures in bold text in our responses, and have included some additional figures in our responses that we have not included in the Figures or Supplementary Figures of the manuscript.

Responses to Editor requests

1. Reviewer 1 raised concerns about the support for certain conclusions and recommended additional methods, such as immunoblotting or calculating Pearson correlation coefficients, to conclusively determine the localization of specific components. They also requested that you assess the impact of translation on some of the mutants.

Response: We have calculated Pearson correlation coefficients (PCC) to quantify the extent of co-localisation between the parasite mitochondrion and numerous CIA pathway proteins (and variants thereof; **Figs 5, 6D, 8E, 8H, 9C and S12F**). We have also performed additional experiments to address the effects of *Tg*Dre2 depletion on the abundance of the cytosolic Fe-S protein *Tg*ABCE1 (**S4C Fig**). We describe all these additions in the detailed responses to the reviewer comments below.

2. Reviewer 2 provided minor suggestions pertaining to figure presentation.

Response: We have attempted to address all the reviewer suggestions, as outlined in our responses to this reviewer below.

3. Reviewer 3 mentioned that some controls might be missing and noted that the conclusion regarding mitochondrial localization being essential for survival is not fully supported by the current data. Like Reviewer 1, this reviewer also pointed out potential issues with your quantification methods, which may contribute to inconsistencies in the results.

Response: We have performed additional experiments to generate and analyse point mutants in the so-called aromatic motif of the CD5 loop in the native *Tg*CIA1 locus (so that this protein is expressed in a native context; **Fig 8B-F** and **S12A-B Fig**). As noted above, we have also calculated PCCs to better quantify the extent of co-localisation between the mitochondrion and various CIA pathway protein variants. In combination, these experiments show clearly that the CD5 loop aromatic motif of *Tg*CIA1 is important for both its function and for its mitochondrial localisation. However, one mutation in this motif (the *Tg*CIA1$_{R533A}$ mutation) that affects mitochondrial localisation (though to a lesser degree than other aromatic motif mutations, per the PCC measurements) does not lead to an impairment of parasite proliferation, suggesting that its impact on *Tg*CIA1 function is minimal. In our opinion, our data cannot fully rule in or out that mitochondrial localisation of *Tg*CIA1 is required for its function. As outlined in our detailed responses to the reviewer (below), we

have attempted to present these data in a nuanced way that leaves open the possibility that the aromatic motif contributes to *Tg*CIA1 function beyond its role in mitochondrial targeting.

Responses to Reviewer 1 comments

Major points

1. a) First, as a readout for mitochondrial, cytosolic or dual localization, the authors mainly use representative images and pixel intensity profiles. Yet, there are a number of experiments for which, as stated by the authors themselves several times in the manuscript, they « cannot rule out that some mitochondrial localization still occurs » and these images are indeed not very conclusive. Perhaps as a result, there are some discrepancies between their conclusion on the localization of some constructs and the impact (or lack of impact) on the parasites. Typical examples include the single amino acid mutants on Fig. 8D and Fig. 9A&B, in which the CIA1 signal still show some extensive overlap with the mitochondrion. I think that a less subjective (and easier to quantify) way of assessing the distribution of the proteins would be through an analysis by immunoblot of protein extracts after mechanical disruption of the parasites and quantifying the relative organelle-associated and cytosolic fractions (ie membrane-associated and soluble forms of the proteins).

Response: We attempted to establish a western blotting-based approach to determine the extent of mitochondrial localisation of *Tg*CIA1 variants that exhibited dual cytosolic/mitochondrial colocalization (wild type *Tg*CIA1), or appeared to localise exclusively to the mitochondrion (*Tg*CIA1$_{\text{CD5-to-CD1}}$) or cytosol (*Tg*CIA1$_{\textit{Sc}\text{CD5}}$). We treated parasites with concentrations of the detergent digitonin that are sufficient to permeabilise the parasite plasma membrane (0.01-0.05% w/v; PMIDs 9813002, 15280394 and 35118179), and separated parasites into soluble (~cytosolic) and pellet (~organellar) fractions before probing by western blotting (example data below). Although the *Tg*CIA1 variant that localised exclusively to the mitochondrion appeared predominantly in the pellet/organellar fraction, we were unable to robustly distinguish differences in the proportion of the cytosolic and dually-localised *Tg*CIA1 variants in the supernatant vs pellet fractions. We concluded that these assays lacked sufficient resolution to test the extent of mitochondrial localisation of CIA pathway proteins. This could be because of limitations of the assay (e.g. incomplete or inconsistent plasma membrane permeabilisation upon digitonin treatment) or because the localisation of *Tg*CIA1 to the mitochondrion is a dynamic (and therefore unstable) process.

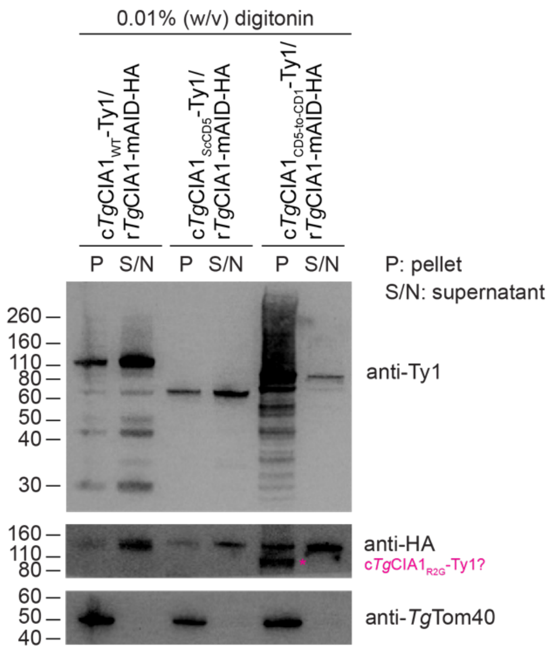

1. b) Alternatively, providing Pearson correlation coefficient values when comparing the signals for the proteins of interest and the mitochondrion on a large number of parasites might allow a more direct comparison and provide more compelling evidence. Again, this is particularly important for data interpretation for a number of fitness phenotypes. For the single amino acid mutants for example, Fig. 8 shows that some are not impacted for fitness, while their localization is currently presented in the manuscript as being largely altered. Similarly, it may be difficult to conclude on the localization of the SmCD5CIA1 and VbCD5CIA1 cell lines are viable in spite of a potential mislocalization (Fig. 9C), although images suggest that both proteins might still be present at the mictochondrion (Fig. 9A&B). Finally, the CIA1ScCD5 cell line is presumably « cytosolic » and severely impacted in growth (Fig. 6), yet translation seems to proceed normally in these parasites (Fig. 10B), which is very surprising. Overall, more clear-cut and quantified data on protein localization may help resolve some of these discrepancies.

Response: As the reviewer suggested, we determined Pearson correlation coefficients (PCCs) between several key CIA pathway proteins (and variants thereof) and parasite mitochondria. These quantifications reveal:

a) That depletion of *Tg*CIA1 leads to a significant reduction of mitochondrial localisation of *Tg*CIA2 and *Tg*MMS19 (**Fig 5A-B**);
b) That depletion of either *Tg*CIA2 or *Tg*MMS19 does not change the extent of mitochondrial localisation of *Tg*CIA1 (**Fig 5C-D**);
c) That replacement of CD5 loop of *Tg*CIA1 with the equivalent loop of *Sc*CIA1, but not exchange of the CD1 or CD3 loops, leads to a significant reduction of mitochondrial localisation in the *Tg*CIA1 protein (**Fig 6D**);
d) That mutating the W526, Y527, F532 and R533 residues of *Tg*CIA1 to alanine significantly reduces the extent of mitochondrial localisation of *Tg*CIA1 (in both

over-expressed and natively-expressed versions), although the R533A mutation exhibits significantly greater mitochondrial co-localisation than the W526A, Y527A or F532A mutations (**Fig 8E**, **Fig 8H** and **S12F Fig**).

e) That replacement of CD5 loop of *Tg*CIA1 with the equivalent loop of *Vb*CIA1 and *Sm*CIA1, leads to a significant reduction of mitochondrial localisation in the *Tg*CIA1 protein (**Fig 9C**).

We refer to these new data quantifications at relevant points throughout the revised manuscript.

2 a). Second, while there seems to be a marked impact of CIA1 depletion on protein translation (Fig. 3C), it seems to be less obvious when depleting the other members of the complex (S4 Fig.). First, there it seems unlikely that these analyses have been performed 24h after adding the IAA (as stated in the figure legends, l. 1174 and l. 1400), because at this timepoint the authors have clearly shown that the decrease in abundance of the ABCE1 protein (which is presumably responsible for the phenotype) only happens at 48 h post IAA addition (Fig. 3A).

As reported in the manuscript, we did use a 24 hr time point post-IAA addition for these experiments. The reviewer is right in pointing out that we see a severe depletion in protein synthesis upon *Tg*CIA1 knockdown (**Fig 3C**) at a time when we observed only a moderate depletion of *Tg*ABCE1 abundance (**Fig 3A-B**). These data could be explained in a variety of ways: a) a minor depletion in *Tg*ABCE1 abundance leads to a major defect in protein synthesis, b) knockdown of *Tg*CIA1 leads to the loss-in-function of other (uncharacterised) Fe-S proteins that are involved in protein synthesis, or c) knockdown of *Tg*CIA1 leads to phenotypic effects that secondarily effect protein synthesis. Given the discrepancy we see between protein synthesis and *Tg*ABCE1 depletion upon *Tg*Dre2 knockdown (see next response), we think point a) is unlikely.

2. b) One could imagine that not all CIA assembly proteins are impacting ABCE1 with exactly the same kinetics and it would have been informative to look for ABCE1 depletion in the mutant cell lines. While I realize it is quite tedious to do so for all the mutant cell lines (Tah18, Der2, Nar1, Cia2, MMS19), I think it would be a priority for the Dre2 mutant for which, quite unexpectedly, no impact on translation is observed. Assessing the impact of Der2 depletion on ABCE1 abundance, perhaps including longer IAA incubation times, would provide a more direct and clearer evidence that Dre2 depletion is not affecting the stability of ABCE1.

As suggested by the reviewer, we have measured the effects of *Tg*Dre2 knockdown on *Tg*ABCE1 abundance (**S4C Fig**). We found that *Tg*Dre2 knockdown does impact *Tg*ABCE1 abundance, although perhaps not to the same extent as *Tg*CIA1 depletion. We conclude in our description of these data in the results that "*Loss of TgDre2, therefore, does impact abundance of the cytosolic Fe-S protein TgABCE1, leaving open the possibility that TgDre2 has a role in cytosolic Fe-S assembly*" (Lines 183-184). These findings suggest a) that

*Tg*Dre2 may be impacting the cytosolic FeS assembly pathway in different ways to *Tg*CIA1 (and the other components of the pathway), and b) that our indirect assays for measuring cytosolic FeS cluster assembly (measuring ABCE1 abundance and protein synthesis) have their limitations. We raise these points in the Discussion section : "*By contrast, TgDre2 localises to the cytosol and is not required for cytosolic protein synthesis, although loss of TgDre2 does impact the abundance of the cytosolic FeS protein TgABCE1* (***S4 Fig***)*. This suggests that, although TgDre2 is critical for parasite proliferation, it may not function in the parasite CIA pathway in the same way as the other CIA pathway proteins* (***Fig 10A***)*. A limitation to this conclusion is that the assays that we have used in this study to measure CIA pathway function, and which have been published in other recent studies [refs], provide only indirect measures for CIA pathway activity (i.e. measure the stability of cytosolic FeS proteins or measure processes that rely on the incorporation of FeS clusters into cytosolic proteins, such as protein translation and lipid droplet biology). A priority for future research in this area will be to develop assays that directly measure the incorporation of FeS clusters into client cytosolic proteins via the CIA pathway in these parasites."* (Lines 599-610)

Minor points

3. Please provide scale bars for all series of microscopy pictures. Done.

4. The authors seem to have chosen the yeast nomenclature for naming the different components of the complex (although MMS19 is also called MET18 in yeast). As these proteins have different names in human and plant models (a nomenclature chosen by other Toxoplasma researchers on the basis that many Fe-S proteins are common with plants due to the presence of a plastid), perhaps mention the protein aliases somewhere in the manuscript so that readers could relate more easily with the existing literature? We have used the nomenclature that we introduced in our previous study examining the cytosolic FeS assembly protein *Tg*NBP35 (PMID 33222310). To address the reviewer's suggestion, we have listed the most commonly used alternative names for these proteins (in yeast, mammals and plants) when we first mention these proteins in the second paragraph of the Introduction. (Lines 53-62)

5. 137-140: the accessibility of the proteins to the degron system is another hint that they are exposed to the cytosolic face of the mitochondrion. That may be briefly mentioned. This is a good point. However, given that we don't include controls testing whether internal mitochondrial proteins can be regulated by proteasomal degradation, we believe this is beyond the scope of what we can conclude from our data.

6. Fig. 4B. The impact of CIA1 depletion on the abundance of other CTC proteins is variable. Perhaps the authors could very briefly discuss that it may be explained by a different stability of these proteins. We have added the following conclusion to the results paragraph where we describe these data: "*These data indicate that TgCIA1 knockdown impacts the stability of the TgCIA2 and, to a lesser extent, the TgMMS19 proteins.*" (Lines 240-241)

7. 308-309. The authors say that the absence of co-immunoprecipitation between CIA1 and NAR1 suggests that they are likely not interacting. The native gel data and the way CIA1 depletion affects CIA2 and MMS19 localization are solid and converging evidence that they

would interact, but as cell lines with tagged proteins of interest have been generated, have the authors performed co-immunoprecipitation experiments between the CTC proteins that would further support their interaction within the complex? As suggested by the reviewer, we have performed co-immunoprecipitation experiments to test whether the CTC proteins *Tg*CIA1 and *Tg*CIA2 interact, and found that, indeed, they do (**S6D Fig**).

8. More emphasis may be put in the discussion on the interesting finding that segregating CIA1 to the mitochondrion severely impacts translation, which implies the need for some shuttling and perhaps more local interaction with cytosolic components for Fe-S cluster transfer. On the other hand, as mentioned above, I am not completely convinced by the lack of impact of a "cytosol only"-localized version of the protein, for the simple reason that the microscopic imaging data currently presented does not allow to completely rule out that some protein is at the mitochondrion.

Our observation that a *Tg*CIA1 variant (*Tg*CIA1$_{ScCD5}$) that is impaired in mitochondrial localisation is still capable of protein synthesis was interesting, and points to some separation of functions between *Tg*CIA1 when it is localised to the cytosol vs the mitochondrion. However, as the reviewer points out, our localisation studies lack the resolution to definitively conclude that no mitochondrial localisation of *Tg*CIA1 is occurring in this mutant protein, although the quantifications we have performed for the revision do suggest that the extent of mitochondrial co-localisation correlates with the various phenotypes we observe (see our response to Comment 1b from Reviewer 1 above). We have modified the text in the discussion to account for the uncertainty around the extent of mitochondrial localisation of the *Tg*CIA1$_{ScCD5}$ protein in this experiment (changed text in red): "*we found that parasites expressing a TgCIA1 variant that lacked the CD5 loop (and is impaired in mitochondrial targeting) is still capable of cytosolic protein synthesis (**Fig 10B-C**), a process that relies on a functional CIA pathway [refs]. This indicates that [4Fe-4S] cluster transfer to cytosolic proteins can still occur in the absence of the CD5 loop of CIA1, suggesting that the CD5 loop of TgCIA1 (and, by extension, the mitochondrial localisation of the CTC) is not required for the maturation of all cytosolic FeS proteins. However, as noted above, our localisation studies lack the resolution to definitively conclude that no mitochondrial localisation occurs in the CD5 loop mutant.*" (Lines 650-657)

Responses to Reviewer 2 comments

Major points

1. Figure 3B, 3D, 4C, 10C: The data are presented as % control making it hard to appreciate the extent of variation In the control. There are two options to plot variation in the control group. First, generate an average value for the control and then plot each control replicate as a % of this average. Alternatively, show the non-normalized data on the supplement. We include the western blotting images from each replicate of these experiments in the supplementary **Replicate Data File**. Additionally, we have included a supplementary **Numerical Data** file in the revision that includes both the 'raw' values and normalised data from the quantifications of the experiments that the reviewer mentions.

2. Figure 8B: If making the claim that the protein abundance of the amino acid mutants are similar to WT, please include a graph with the protein abundance values below. In response to other reviewer comments (see Reviewer 3, comment 1), we generated point mutants in three of the four residues of the aromatic amino acid motif of the CD5 loop in the native *Tg*CIA1

locus using CRISPR/Cas9 genome editing. We performed western blotting that revealed all these proteins are present at similar abundances (**Fig 8B**), and, as suggested by Reviewer 2, quantified band intensities to support this conclusion (**S12B Fig**).

3. The authors speculate that association of the CIA pathway arose out of need to efficiently capture iron, during evolution of the myzozoan ancestor. However, it is not clear why localizing the CIA proteins to the outer member would offer much advantage over simple diffusion given the small cytosolic compartment of the parasite cell. Doesn't this imply that direct interaction between CIA proteins in the outer surface of the mitochondrion with FeS cluster clients is required and may be the reason for the importance of the association? Figure 10 alludes to this, but other more speculative alternatives are also offered. It would help to provide more clarity on what the authors think is the mostly likely explanation and why. The short answer is that we cannot know for certain why the CIA pathway of myzozoans evolved to localise at the mitochondrial outer membrane. As we attempt to highlight in **Fig 10D**, we find it striking that the evolution of myzozoans coincided with the evolution of the mitochondrial targeting transmembrane domain of NBP35 and the mitochondrial targeting CD5 loop of CIA1 (and by extension the mitochondrial targeting of the CTC), in addition to mitochondrial targeting transmembrane domains of normally cytosolic/nuclear FeS proteins such as Elp3 and RlmN, and the acquisition of a chloroplast. One thing that links all of these processes is a requirement for iron, which is a limiting nutrient in the marine environments where myzozoans evolved. We posit that the migration of the CIA pathway and cytosolic FeS proteins to the mitochondrion might be connected to more efficient iron usage, but have added caveats to this discussion paragraph to note that "*The selective forces that shaped this reorganisation of the cytosolic FeS cluster assembly pathway remain unclear*" (Lines 683-684) and that "*the extent to which the localisation of the CIA pathway to the mitochondrion increases the efficiency of iron usage is unclear.*" (Lines 698-699)

Minor points

4. Line 21: 'clusters' not 'cluster'. Changed

5. Line 103, 108, etc: Authors do not need to refer readers to PCR verification of strains utilized in every figure. Citation is enough referencing supplemental figures is enough. We have kept the reference to the PCR screening analysis in the first instance that we describe this (**S1A-E Fig**), and, as suggested by the reviewer, just reference the supplementary figures that validate the integrations thereafter. We have added additional clarification to the section of the methods where we describe these PCR verification approaches: "*performed PCR screening analyses to identify genetically modified parasites using gene specific "3' or 5' scrn fwd" and "3' or 5' scrn rvs" primers*". (Lines 761-763)

6. Figure 1F: Western blot of tagged MMS19-mAID-HA shows two major bands. Are both bands corresponding to the protein? (one cleaved and one un-cleaved). If not, which is

considered the protein of interest? We see a 'double' MMS19 band on some occasions (Fig 1F, S4A Fig) but not others (Fig 2F, Fig 4B, Fig 4E). This lower band possibly represents a degradation product, although we cannot be certain of this. We have added the following text to the legend of Fig 1: "*TgMMS19-HA appears as two separate bands, the lower of which may represent a degradation product.*" (Lines 1216-1217)

7. Figure 6E: Move the color-coded key for -IAA / + IAA to the bottom so it's clear it goes with all graphs in E. Done (see **Fig 6F**)

8. Figure 6E: There appears to be a slight fitness defect for parasites with the ScCD1 mutation- is there any speculation as to why this confers a fitness defect but not a localization difference of CIA1? The moderate fitness cost we observe in the r*Tg*CIA1-mAID-HA/*Tg*CIA1-$_{ScCD1}$-Ty1 line upon the addition of IAA could be the result of the aberrant mitochondria that are apparent in this line, although these aberrant mitochondria are also apparent in the -IAA condition (**S10B Fig**) when we don't observe a proliferation defect. Curiously, we observe fewer plaques in +IAA condition of the corresponding plaque assay (**S10C Fig**), indicative of an effect on parasite viability or invasion when parasites express only the *Tg*CIA1$_{ScCD1}$-Ty1 isoform. These data suggest a possible role for the CD1 loop in processes that affect the viability of extracellular parasites or parasite invasion. We have modified the text to point this out to readers: "*cTgCIA1$_{ScCD1}$-Ty1-expressing parasites exhibited a moderate proliferation defect upon TgCIA1-mAID-HA depletion, resulting in fewer (rather than smaller) plaques (**Fig 6F; S10C Fig**). This suggests a possible role for the CD1 loop in processes that affect the viability of extracellular parasites or the ability of parasites to invade host cells.*" (Lines 396-399)

9. All figures containing growth curves: Can statistical values be plotted on the graph? We plot these graphs relative to the time (in hours) after we set up the assays. Because we read the independent repeats at different hours, it is not possible to plot data points from multiple independent experiments on a single graph, and therefore it is not possible to undertake statistical analyses on these data for any particular timepoint. As we note in the figure legends, the error bars on these proliferation curves depict the variation in multiple replicate wells from an individual experiment (i.e. depict technical replicates). The curves we include in the manuscript are all representative of at least three independent repeats (which are all highly consistent). We include data from the independent experiments in the **Replicate Data File**.

10. Figure 8E: move color coded key to a more visible location. Statistical values plotted would also be great. We have reorganised this figure so that the F532A data are now in **Fig 8I**, and the remaining data are in **S12G Fig**. In both instances, we have placed the key below the graphs to make this more visible. Regarding plotting statistical values, see our response to the previous query.

Responses to Reviewer 3 comments

Major points

1. A major claim of the paper is that the mitochondrial localization of the CTC is required for parasite survival. However, the authors' data indicates otherwise. Fig. 8D-E show CIA1 mutants that have cytosolic localization, yet still result in viable parasites. Additionally, Fig. 10B-C demonstrates that a cytosolically-localized mutant of CIA1 is able to sustain translation, whereas a mitochondrially-localized mutant of CIA1 is not. The authors present this translation result as surprising, but it agrees with their earlier finding in Fig. 8 that cytosolic CIA mutants remain viable. Together, these results contradict the authors' statement that "the mitochondrial localization of the CTC is critical for parasite survival".

We thank the reviewer for raising this point. The data we present in the original manuscript indicate that a) the CD5 loop of is responsible for mitochondrial targeting of *Tg*CIA1, b) all the tested residues of the aromatic motif of the CD5 loop are important for mitochondrial targeting, c) the CD5 loop is important for at least some of the essential functions of *Tg*CIA1 but apparently not for protein translation, and d) some, but not all, residues of aromatic motif of the CD5 loop are important for *Tg*CIA1 function. The reviewer is right to point out that parasites expressing several *Tg*CIA1 variants that appear to localise exclusively to the cytosol can still proliferate normally (the Y527A and R533A mutants and, to a lesser extent, the W526A mutant). As Reviewer 1 points out (comment 1), and as we pointed out in our original submission, a limitation of our interpretation of these experiments is that we cannot be certain that there is no mitochondrial localisation upon mutating these residues, particularly given that we are overexpressing the *Tg*CIA1 variants from the constitutive α-tubulin promoter.

We reasoned in our original manuscript that overexpression of the *Tg*CIA1 variants may enable sufficient (perhaps non-specific) mitochondrial localisation to mask the proliferation phenotypes associated with the aberrant targeting. To test this further, we attempted to generate CD5 loop aromatic motif mutations in the native *Tg*CIA1 locus using genome editing approaches. We were unsuccessful in generating a mutant in the F532 residue, which wasn't surprising given that even an over-expressed version of the mutant protein is unable to complement the loss of wild type *Tg*CIA1 (**Fig 8I**). We were able to generate W526A and Y527A mutants. Consistent with the overexpression data, these proteins exhibited a defect in mitochondrial localisation (new **Fig 8D-E**). Notably, however, parasites expressing these proteins (which were present in similar abundance to the wild type *Tg*CIA1 protein and also present in the CTC; new **Fig 8B-C, S12B Fig**) exhibited severe proliferation defects (new **Fig 8F**). This is consistent with the hypothesis that affecting the mitochondrial localisation of these proteins does impact parasite fitness. We also generated an R533A mutant. Again, consistent with the over-expression data, this protein appeared aberrant in mitochondrial localisation, although (based on the Pearson correlation coefficient data we obtained in response to Reviewer 1, comment 1) the R533A protein appeared to exhibit greater mitochondrial co-localisation than the other aromatic motif mutants (new **Fig 8E**, which was

also observed in the over-expression experiments in **S12F Fig**). The R533A mutant exhibited no proliferation defect, suggesting that its impact on *Tg*CIA1 function is minimal.

From these new data, we believe two interpretations are possible:

a) That the mitochondrial localisation of *Tg*CIA1 (as conferred by the CD5 loop) is important for *Tg*CIA1 function. In this scenario, the slightly higher degree of R533A localisation to the mitochondrion compared to the other aromatic amino acid mutants is sufficient to enable sufficient *Tg*CIA1 function for normal parasite proliferation.
b) That the CD5 loop, and the aromatic amino acid motif therein, has two separate functions. It is responsible for mitochondrial targeting, a process that is not essential for *Tg*CIA1 function. It also has a second, essential function that it unrelated to its role in mitochondrial targeting (and also unrelated to the contribution of *Tg*CIA1 for protein translation).

In addition to the new data and the corresponding description of these in the Results section, we have modified the text to be clearer about the possible roles of the CD5 loop and the limitations of what we can conclude from our data. Specifically:

i) We have modified the abstract to be clear that the *Tg*CIA1 CD5 loop (rather than mitochondrial targeting of *Tg*CIA1 per se) is critical for parasite survival. "*We reveal that mitochondrial targeting of the CTC is mediated by a novel loop on the CIA1 protein of the complex, and that this loop is critical for parasite survival.*" (Lines 28-30)

ii) We have modified the final paragraph of the introduction to be clear that the CD5 loop, rather than mitochondrial targeting per se, is critical for parasite biology: "*Curiously, we found that the CTC proteins TgCIA1, TgCIA2, and TgMMS19 exhibit a dual localisation to the mitochondrion and cytosol, with mitochondrial localisation of this complex mediated by a loop on the TgCIA1 protein that is conserved throughout myzozoans, and which is critical for its role in parasite biology.*" (Lines 87-91)

iii) We have expanded the discussion paragraphs that cover these results: "*We found that deleting the CD5 loop or modifying key residues in the aromatic amino acid motif impairs both mitochondrial targeting and TgCIA1 function. This indicates that the CD5 loop, and the aromatic motif therein, plays a critical role in TgCIA1 (and by extension, CTC) function. One intepretation of these data is that the mitochondrial localisation of TgCIA1 is essential for its function. Curiously, however, mutating the arginine residue at position 533 in the aromatic amino acid motif to alanine impairs mitochondrial targeting, but does not ablate protein function (**Fig 8**; **S12 Fig**). It is possible that the aromatic amino acid motif in the CD5 loop plays two independent roles in TgCIA1 biology: a non-essential role in mitochondrial targeting and an essential role in protein function. A limitation of our localisation experiments is that they cannot rule out that some mitochondrial association remains in the TgCIA1$_{R533A}$ mutant protein, and indeed quantifications of the extent of overlap between the various point mutants we generated in this study consistently suggest a greater degree of overlap between the TgCIA1$_{R533A}$ protein and the mitochondrion than the other point mutants in the aromatic motif (**Fig 8**; **S12 Fig**).*

An important remaining question is therefore what role(s) the CD5 loop and aromatic amino acid motif play in TgCIA1 protein function. We considered the possibility that the mitochondrial

*association of TgCIA1 that is facilitated by the CD5 loop positions the CTC to receive FeS clusters from TgNar1 at the mitochondrial outer membrane (**Fig 10Ai-ii**). However, we found that parasites expressing a TgCIA1 variant that lacked the CD5 loop (and is impaired in mitochondrial targeting) is still capable of cytosolic protein synthesis (**Fig 10B-C**), a process that relies on a functional CIA pathway [refs]. This indicates that [4Fe-4S] cluster transfer to cytosolic proteins can still occur in the absence of the CD5 loop of CIA1, suggesting that the CD5 loop of TgCIA1 (and, by extension, the mitochondrial localisation of the CTC) is not required for the maturation of all cytosolic FeS proteins. However, as noted above, our localisation studies lack the resolution to definitively conclude that no mitochondrial localisation occurs in the CD5 loop mutant.*" (Lines 632-657)

2. The manuscript relies heavily on immunofluorescence assays. However, in no instance do the authors provide an image of an untagged control strain in their experiments. Background signal is a common occurrence in immunofluorescence imaging, and could be contributing signal to colocalization quantifications. For this reason, untagged controls must be included for all immunofluorescence assays.

We use the antibodies we described in the manuscript on a regular basis in our lab and we have a good feeling for what non-specific labelling looks like. To directly address the reviewer's concerns, we undertook immunofluorescence assays on an untagged parasite strain (RH TATiΔ*ku80*; PMID 22144892), alongside positive controls that expressed HA, Ty1 and cMyc fusions (TgCIA1-mAID-HA/TgCIA1-smFP-cMyc for the anti-HA and anti-cMyc IFAs, and cTgCIA1-Ty1/TgCIA1-mAID-HA for the anti-Ty1 IFAs). We probed with 1:200 diluted anti-HA, anti-cmyc or anti-Ty1 primary antibodies and 1:1500 anti-*Tg*Tom40 primary antibody to label the mitochondrion, and then probed with donkey anti-rat AlexaFluor 488 1:500 for the anti-HA IFA, goat anti-mouse AlexaFluor 488 1:200 for the anti-cMyc experiment, and goat anti-mouse AlexaFluor 488 1:500 for the anti-Ty1 experiment, as well as goat anti-rabbit AlexFluor 647 1:500 for the anti-*Tg*Tom40 labelling. We used the same antibody dilution on the same day for both the TATiΔ*ku80* line and the corresponding positive control cell lines for each antibody. For the imaging itself, we used the same exposure time and other image settings for both the TATiΔ*ku80* and control lines for each antibody. For the image processing, we adjusted contrast and brightness of one of the positive control images linearly, and then used the same adjustments (i.e. same minimum and maximum pixel intensities) for the TATiΔ*ku80* images and other positive control images, meaning that the untagged control images are directly comparable to the corresponding controls. We include representative images from these experiments below, and note the minimal amount of labelling observed in TATiΔ*ku80* parasites compared to the tagged line.

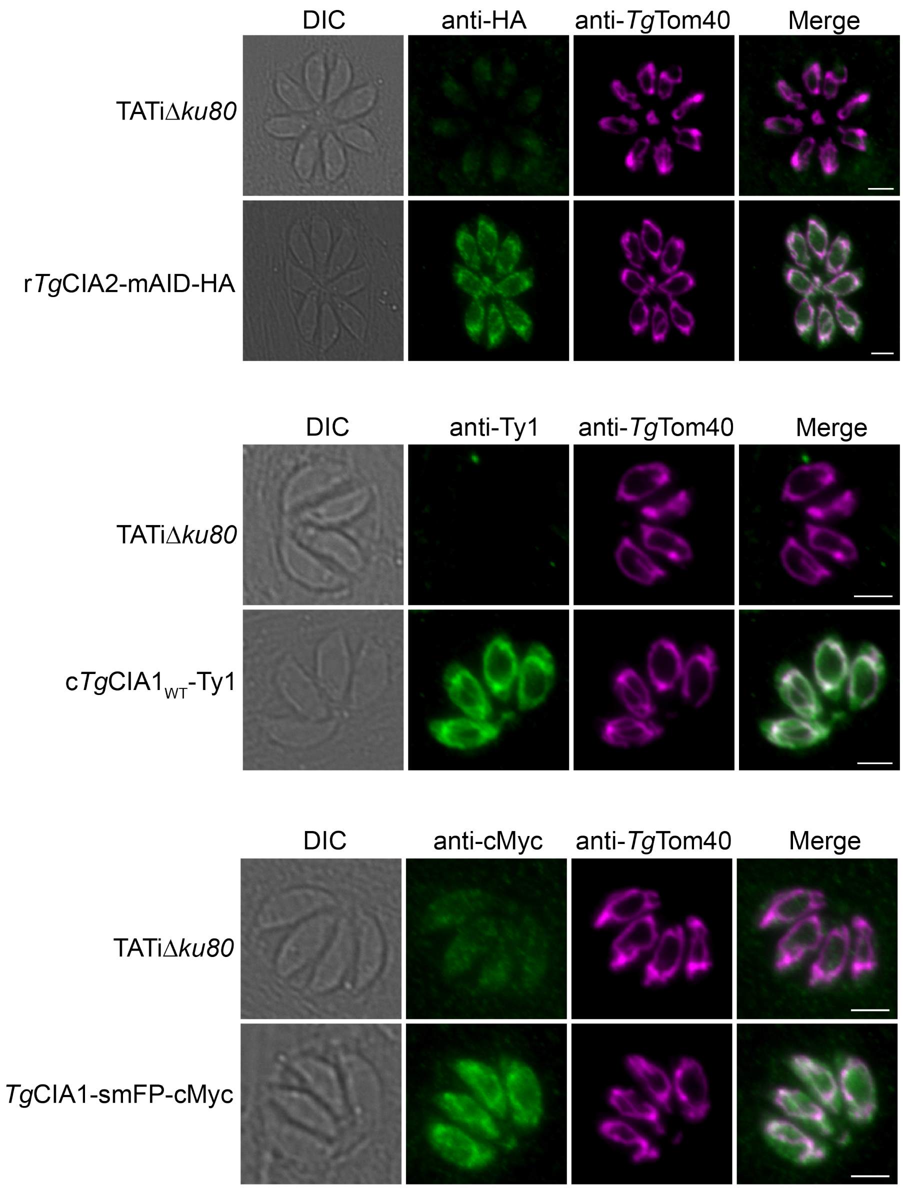

3. Additionally, the authors show and quantify a single vacuole for many of their immunofluorescence assays. It would benefit the manuscript if the authors could comment on the penetrance of the phenotypes they observed. This would be particularly needed in instances in which the authors claim a subtle mitochondrial colocalization despite a dominant cytosolic signal, such as in Fig. 9B.

We include additional representative images in the supplementary **Replicate Data File**. Additionally, we have calculated Pearson Correlation Coefficients for the degree of overlap between the tagged proteins and the mitochondrion in numerous experiments, including **Fig 9** (see our response to Reviewer 1, Comment 1).

4. The authors calculate colocalization signal along a straight line through the parasites. However, the parasite nucleus is relatively large, and will exclude signal from both the

cytosol and the mitochondrion. For this reason, straight lines that cross through the nucleus are likely to artificially inflate colocalization measures. The authors should consider other measures for calculating colocalization, such as simply determining the total overlapping area of two signals, or at a minimum comment on the impact of the nucleus on their colocalization metric.

We agree with the reviewer (and Reviewer 1) that quantifying the extent of co-localisation is a particular challenge for our manuscript. As noted in our response to Reviewer 1, Comment 1, we have calculated Pearson Correlation Coefficients to test the extent of overlap between the tagged proteins and the mitochondrion in numerous experiments. We believe that the fluorescence intensity plots are still a useful way to visually represent the data, and have kept these in the figures of the revised manuscript.

5. The authors claim that CIA1 depletion both results in the depletion of CIA2 and a lack of mitochondrial localization of CIA2. These results seem inconsistent with each other. Indeed, there appears to be no obvious decrease in CIA2-HA signal in Fig. 5A upon CIA1 knockdown, inconsistent with the western blot results in Fig. S6B showing CIA2-HA depletion. This discrepancy highlights the issues that arise from the above concerns that the authors show a single vacuole for most immunofluorescence assays, and fail to include untagged controls that would enable one to observe background signal.

As the western blot in **S6B Fig** shows, the *Tg*CIA2-HA protein is depleted but not completely lost upon *Tg*CIA1-mAID-cMyc knockdown. We are still able to detect the *Tg*CIA2-HA protein in immunofluorescence assay experiments (**Fig 5A**, though we note that the images presented in this figure were linearly adjusted to different extents, giving the appearance of similar brightness in the -IAA and +IAA conditions). See our response to Comment 2 from Reviewer 3 regarding the untagged controls – we note that the minimal background observed in the anti-HA labelling of the untagged control appears very different to the *Tg*CIA2-HA labelling in the +IAA condition in **Fig 5A**.

Minor points

6. The authors do not describe how they identified the parasite candidate homologs of the CIA proteins tagged in Fig. 1. Given the reported divergent localization of many of these CIA proteins, it would be beneficial to provide some detail on how these homologs were assigned. We described this in a previous publication (Aw et al, PMID 33222310 – see Figure 1b of that manuscript and associated text). We reference this in the introduction of this manuscript: "*The core proteins involved in the CIA pathway, including all three proteins of the CTC, are conserved in apicomplexans [Aw et al reference].*" (Line 73-74)

7. It may simply be the resolution of the provided figures, but in many cases the yellow line used for colocalization measurements is difficult to see on the immunofluorescence images. We have increased the stroke thickness from 0.25 point to 0.5 point on all the

immunofluorescence images, attempting to achieve a balance between visibility of the line and obstruction of the image itself.

8. It is customary to place figure panels in the order in which they appear in the text. As Fig. 2B-G are discussed in the text before Fig. 2A, the authors should consider moving panel 2A to the end of the figure. Done.
