# Supplementary material for: A novel targeting domain directs essential components of the cytosolic iron–sulfur cluster assembly pathway to the mitochondrion of Toxoplasma parasites
Source: PLoS Biol. 2025 Nov 25;23(11):e3003520. doi: 10.1371/journal.pbio.3003520 (PMC12674569; doi:10.1371/journal.pbio.3003520)
Supplement: Attachment — Submitted filename: Response_to_reviewers_auresp_3.pdf [file pbio.3003520.s019.pdf]

<u>Response to editor and reviewer comments and suggestions</u>

We again thank the reviewers and editors for taking the time to read through the manuscript and providing feedback. We have responded to each of the comments from the editors and reviewers, with the queries in black text and our responses in blue text.

<u>Editor comments</u>

The Academic Editor has requested that the immunofluorescence negative control data in R2R (reviewer 3, major comment 2) be provided as a new supplemental figure.

We have included these data as S13 Fig (figure legend lines 1702-1713) and refer to the data when we describe the immunofluorescence assays in the Methods section (Lines 879-882).

1) We routinely suggest changes to titles to ensure maximum accessibility for a broad, non-specialist readership, and to ensure they reflect the contents of the paper. In this case, we would suggest a minor edit to the title, as follows. Please ensure you change both the manuscript file and the online submission system, as they need to match for final acceptance:

"Mitochondrial targeting of the cytosolic iron–sulfur assembly machinery is necessary for parasite survival in Toxoplasma gondii"

As per our email discussion, we feel that the proposed title is not an entirely accurate representation of our study. We have instead modified the title to read:

"A novel targeting domain directs essential components of the cytosolic iron-sulfur cluster assembly pathway to the mitochondrion of *Toxoplasma* parasites"

2) Please add the link of the funding agencies in the Financial Disclosure statement in the manuscript details.

We have added relevant urls for each of the funders listed in the financial disclosure statement. This now reads as follows (with changes in red): "This research project was supported by grants from the Australian National Health and Medical Research Council (GNT1182369; https://www.nhmrc.gov.au/) and Australian Research Council (DP230100853; https://www.arc.gov.au/) to GGvD, an Australian Government Research Training Program scholarship to JAH (https://www.education.gov.au/research-block-grants/research-training-program), and a Gwendolyn Woodroofe PhD scholarship from the ANU to RAL (https://study.anu.edu.au/scholarships/find-scholarship/anu-gwendolyn-woodroofe-phd-scholarship-biological-sciences). The funders did not play any role in the study design, data collection, data analysis, decision to publish, or preparation of the manuscript."

3) Please note that per journal policy, the model system and species studied should be clearly stated in the abstract of your manuscript (Toxoplasma gondii).

We have modified the abstract text to read "Here, we undertake a comprehensive analysis of the CIA pathway in *T. gondii* parasites."

4) Thank you for providing all the raw values for the figures. We noticed that perhaps the values of 9C or 9D might be mislabeled in the tab for Fig 7.

Thank you for picking this up. We have changed the labelling from Fig 9C to Fig 9D in the tab for Fig 7.

5) Please cite the location of the data clearly in all relevant main and supplementary Figure legends, e.g. “The data underlying this Figure can be found in S1 Data” or “The data underlying this Figure can be found in

We have added the text: “The numerical data underlying this Figure can be found in **S1 Data**” to all relevant figure legends (Figs 1-10, S4 Fig, S6 Fig, S8 Fig, S10 Fig, S11 Fig and S12 Fig).

6) Supplementary files (e.g., excel). Please ensure that all data files are uploaded as 'Supporting Information' and are invariably referred to (in the manuscript, figure legends, and the Description field when uploading your files) using the following format verbatim: S1 Data, S2 Data, etc. Multiple panels of a single or even several figures can be included as multiple sheets in one excel file that is saved using exactly the following convention: S1_Data.xlsx (using an underscore).

We have changed the name of the document containing the numerical data from “**Numerical Data**” to “**S1_Data**” and now refer to this as “**S1 Data**” in the main text (Lines 997 and 1723). Additionally, we have renamed the ‘Replicate data’ file (which contains replicates of various ‘representative’ data we include in the manuscript) to **S2 Data** (Lines 995 and 1726).

7) Please make sure that all figures use a colorblind-friendly palette.

We believe we have done this. Fluorescence microscopy images and fluorescence profiles have been coloured green and magenta. Proliferation assays use black to denote the -IAA conditions and red to denote the +IAA conditions. In some diagrams where we use shades of green and red together (e.g. Fig 6A, Fig 7B) we provide additional labels to be clear what the coloured sections of the figure refer to.

8) Thank you for providing the original, uncropped and minimally adjusted images supporting all blot and gel results. Please rename the pdf file to ‘S1_raw_images’.

Done. We also now refer to this as “S1 Raw Images” in the manuscript text (Lines 996 and 1719)

9) Please ensure that your Data Statement in the submission system accurately describes where your data can be found and is in final format, as it will be published as written there

The Data Statement is correct (“All relevant data are within the paper and its Supporting Information files.”)

10) Per journal policy, if you have generated any custom code during the course of this investigation, please make it available without restrictions. Please ensure that the code is sufficiently well documented and reusable, and that your Data Statement in the Editorial Manager submission system accurately describes where your code can be found.

We have not generated any custom code in this study.

Reviewer 1

- l. 68. and Fig. 9D. Are Perkinsozoa considered 'dinoflagellates' per se, or are they more precisely a sister lineage to Dinoflagellata, occupying an early branching phylogenetic position within the Dinozoa? This distinction doesn't alter the fact that they are myzozoans and, therefore, has no impact on the message of the paper, but it appears to be presented sometimes in a confusing way in the literature.

Response: We agree that "Dinozoa" would be the more appropriate term in referring to perkinsids and dinoflagellates collectively. We have changed the text in the introduction to replace dinoflagellates to dinozoans (Line 67). We additionally refer to dinozoans (instead of dinoflagellates) elsewhere in the text (lines 70, 346, 516, 1366, 1615). We have also modified Figure 10D to change 'Dinoflagellates' to 'Dinozoans'

- l. 600-603. The impact of TgDre2 depletion on TgABCE1 abundance, but not on overall protein synthesis, presents contradictory phenotypes. This suggests that, although TgABCE1 is expressed at lower levels, it remains functional and likely retains its Fe-S clusters. However, as noted in the discussion section, further assays (such as 55Fe incorporation and specific immunoprecipitation/quantification) would be needed to confirm this. The question then arises: why is TgABCE1 abundance affected by TgDre2 depletion if the latter is not involved in the assembly of cytosolic Fe-S clusters? In the case of TgCIA1 depletion, the strong impact on translation (evaluated after 24h of IAA treatment, Fig. 3C) appears before there is a strong decrease in TgABCE1 abundance (mostly seen at 48h of IAA treatment, Fig. 3A) : this suggests that the impact on TgABCE1 Fe-S cluster maturation is rapid and precedes the potential lack of stability and the degradation of the apo form that subsequently would lead to a decrease in protein abundance. The effect of TgDre2 depletion on TgABCE1 abundance appears to be delayed and less pronounced compared to those observed TgCIA1 depletion, but then it is intriguing that there would be a decrease in TgABCE1 abundance at 24h of IAA treatment while there is no impact on translation (Fig. S4C). This doesn't really fit with TgABCE1's Fe-S cluster assembly being affected prior to an impact on protein stability (if relying on the TgCIA1 data). This is just a comment here, there is no need to necessarily modify the discussion but, if anything, it shows that it is still a very open question.

Response: Yes, we agree. As we point out in the discussion (line 603-610), the assays currently available for monitoring cytosolic Fe-S cluster assembly are indirect, which limits the conclusions that we can draw from them. Important questions to address in this area for the future include: a) determining the identity of client proteins of the CIA pathway, b) identifying which client proteins contribute to cytosolic protein translation, and c) further characterising the role of the *Tg*ABCE1 protein in these parasites (and its contribution to protein translation).
